# Epidemiological analysis reveals a surge in inflammatory bowel disease among children and adolescents: A global, regional, and national perspective from 1990 to 2019 – insights from the China study

**DOI:** 10.7189/jogh.13.04174

**Published:** 2023-12-01

**Authors:** Zhong-mian Zhang, Zi-li Lin, Bai-xiang He, Wei-tian Yan, Xi-yan Zhang, Zhong-han Zhang, Lan Wang, Jia-qi Wang, Da-ming Liu, Wen Zhang, Zhi-hong Li

**Affiliations:** 1 Department of Gastroenterology, Dongzhimen Hospital, Beijing University of Chinese Medicine, Beijing, China; 2 Rheumatology Department, Yunnan Provincial Hospital of Traditional Chinese Medicine, Yunnan University of Chinese Medicine, Yunnan, China; 3 College of Psychology and Mental Health, North China University of Science and Technology, Hebei, China

## Abstract

**Background:**

The burden of inflammatory bowel disease (IBD) among children and adolescents is rising globally, with substantial variation in levels and trends of disease in different countries and regions, while data on the burden and trends were sparse in children and adolescents. We aimed to assess the trends and geographical differences in children and adolescents aged zero to 19 in 204 countries and territories over the past 30 years.

**Methods:**

Data on IBD among children and adolescents was collected from the Global Burden of Disease (GBD) 2019 database from 1990 to 2019. We used the GBD data and methodologies to describe the change in the burden of IBD among children and adolescents involving prevalence, incidence, disability-adjusted life years (DALYs), and mortality.

**Results:**

Globally, the IBD prevalence cases increased between 1990 and 2019. Annual percentage changes (AAPC) = 0.15; 95% confidence interval (CI) = 0.11-0.19, and incidence cases of IBD increased from 20 897.4 (95% CI = 17 008.6-25 520.2 in 1990 to 25 658.6 (95% CI = 21 268.5-31 075.6) in 2019, representing a 22.78% increase, DALYs cases decreased between 1990 and 2019 (AAPC = -3.02; 95% CI = -3.15 to -2.89), and mortality cases of IBD decreased from 2756.5 (95% CI = 1162.6-4484.9) in 1990 to 1208.0 (95% CI = 802.4-1651.4) in 2019, representing a 56.17% decrease. Decomposition analysis showed that IBD prevalence and incidence increased significantly, and a trend exhibited a decrease in underlying age and population-adjusted IBD DALYs and mortality rates. Correlation analysis showed that countries with high health care quality and access (HAQ) had relatively higher IBD age-standardised prevalence rate (ASPR) and age-standardised incidence rate (ASIR), but lower age-standardised DALYs rate (ASDR) and age-standardised mortality rate (ASMR).

**Conclusions:**

Global prevalence and incidence rate of IBD among children and adolescents have been increasing from 1990 to 2019, while the DALYs and mortality have been decreasing. Rising prevalence and rising incidence in areas with historically low rates will have crucial health and economic implications.

Inflammatory bowel disease (IBD) is a non-infectious, chronic inflammatory, multifactorial condition encompassing Crohn’s disease (CD), ulcerative colitis (UC), and indeterminate colitis. It is estimated that 25% of IBD develops in childhood or adolescence [1,2], and the incidence has tripled in the past 20 years [3,4]. This chronic disease places a heavy burden on children and adolescents, affecting their physical, social, and mental health and education [5-9]. Multiple studies [10-14] have investigated the impact of IBD on health-related quality of life, showing that symptoms and complications can harm the daily lives of children and adolescents and their families, affecting all areas of health. Studies specialising in IBD [15-17] have also shown that these IBD patients miss more school than their healthy peers, although this is not necessarily associated with poorer educational outcomes. And a recent study [18] indicates that IBD hurts school/university attendance, with hospitalisation rates, disease burden, and learning difficulties being major barriers.

The forces of global population growth and epidemiological shifts have shaped the epidemiology of non-communicable diseases, including hypertension and diabetes, and most likely the epidemiology of IBD. However, studies have demonstrated that the global burden of the total IBD population increased significantly with geographic variations [19,20]. However, a detailed quantitative analysis of IBD among children and adolescents at the global, regional, and national levels over the past 30 years is not available.

We examined the Global Burden of Disease (GBD) data from 1990 to 2019 to describe the state of IBD in children and adolescents epidemiology at the global, regional, and national levels, examine how demographic and epidemiology drivers shaped the change in the burden of IBD in children and adolescents over this period, and clarify the relationship between the burden of IBD in children and adolescents and indicators of health and economic prosperity in any given country.

## METHODS

### Data sources

GBD 2019 provides the most up-to-date estimation of the epidemiological data of 369 diseases and injuries in 21 GBD regions and 204 countries and territories from 1990 to 2019. We obtained repeated cross-sectional data from the Global Health Data Exchange (GHDx) query tool [21], which includes annual prevalence cases and rate (per 100 000 population), incidence cases and rate (per 100 000 population), disability-adjusted life years (DALYs) count and rate (per 100 000 person-year), and mortality cases and rate (per 100 000 population) by sex, age, region, and country. Details of the methodology used in the GBD 2019 can be found in previous studies [19,22].

International Classification of Disease version ten (ICD-10) codes were K50 for Crohn’s disease, K51 for ulcerative colitis, and K52 for indeterminate colitis. In GBD 2019, prevalence cases of IBD were extracted if a person had at least one inpatient or two outpatients with appropriate ICD codes and valid case definitions from the United States (US) data set and literature reviews, which differed from the case extraction method prevalent in GBD 2017, wherein a person was extracted if he has only at least one clinic visit.

The Institute for Health Metrics and Evaluation (IHME) provides only aggregated and deidentified data, which is responsible for administering the Global Burden of Disease Study. The institutional review board of the Dongzhimen Hospital Affiliated Beijing University of Chinese Medicine determined that the study did not need approval because it used publicly available data. This study adheres to the Guidelines for Accurate and Transparent Health Estimates Reporting (GATHER) [23].

### Prevalence and incidence data

We used the epidemiologic state transition disease modeling software DisMod-MR 2.1, a Bayesian compartmental model widely used in GBD non-fatal modeling, to generate initial estimates of IBD prevalence and incidence. We adjusted for differences in study levels in measurement methods and case definitions. We estimated exposure to risk factors using a population-representative survey and monitoring data and regression models with geospatial Gaussian processes [20].

### DALYs and mortality data

We estimated the mortality rates using vital registration data encoded by the ICD system or household mortality surveys known as autopsy. We used statistical methods to increase the comparability of mortality data sources, including reclassification of non-specific or unspecified codes, noise reduction algorithms, and Bayesian geospatial regression software (CODEm, Institute for Health Metrics and Evaluation, Seattle, Washington, USA), which uses site-specific covariates to create smooth time trends for 204 countries and territories by borrowing the intensity of age, space, and time. GBD 2019 allows the uncertainty intervals (UIs) to be used to estimate all locations from year to year, even when data are sparse or missing.

DALYs for a disease or health condition are an index of overall disease burden and are defined as the sum of years of life lost due to premature mortality (YLLs) and the years lived with a disability (YLDs) due to the prevalence cases of the disease or health condition in the population [24]. Comorbidities were adjusted for the independent probability of their prevalence exposure to acquired conditions by simulating 40 000 individuals per age, sex, country, and year. In GBD 2019, bias adjustment methods were designed to facilitate direct comparisons between different case definitions and study designs. The 95% UIs reported by each estimate are derived from the model’s posterior distribution using 1000, reported as the 25th and 975th distribution values. Age standardisation uses a direct approach, using the global age structure from 2019.

### Children and adolescents’ definition

According to the World Health Organization (WHO) definition, children and adolescents are individuals aged zero to 19. We divided the whole data into four age groups: young children (<5 years), older children (5-9 years), younger adolescents (10-14 years), and older adolescents (15-19 years) [25-27].

### Sociodemographic index

According to the results of the GBD 2015, the Institute for Health Metrics and Evaluation (IHME) proposed a new developmental classification indicator. It is the sociodemographic index (SDI) (ranging from zero to one), which is composed of a total fertility rate under 25 (TFU 25), lag distributed income per capita (LDI) and mean education for those aged 15 and older (EDU 15+), which was closely related to population health outcomes and social development status. Meanwhile, the GBD 2019 categorised 204 countries and territories into 21 geographic regions. SDI divides countries and territories into five categories (high SDI, high-middle SDI, middle SDI, low-middle SDI, and low SDI levels) [22]. The numeric value is 1, indicating that the highest TFU was 25, the highest LDI, and the highest EDU was 15+, meaning that the region had the highest theoretical level of development related to health outcomes. The SDI value of 0 was the opposite.

### Healthcare Quality and Access (HAQ) index

The HAQ index [28] provides a new summary measurement from 0 (worst) to 100 (best) to generate an interpretable single measure that facilitates a comparable assessment of access to and quality of personal health care in 195 countries and territories over time and at different stages of development. It is based on risk-standardised mortality rates for 32 causes of GBD that are deemed to require personal care.

### Data processing and disease model

First, we assessed the global trends in IBD’s prevalence, incidence, DALYs, and mortality rate. We used the Joinpoint regression model with logarithm-transformed rates to calculate the average annual percent changes (AAPCs), as the dependent variable and year as the independent variable. The AAPC is a pre-specified aggregate measure of trends over fixed intervals and is calculated as a weighted average of annual percentage change (APC), allowing a single number to be used to describe the average APCs over multiple years. We calculated AAPCs using the geometrically weighted average of the various APC values in the regression analysis [29].

The AAPCs indicate the number of APC values that change annually (e.g. an increase, decrease, or no change). The trends of the investigated rates were thereby reflected in AAPCs and their associated 95% confidence interval (CI). We calculated the AAPCs between 1990 and 2019.

Second, we used the decomposition methodology of Das Gupta [30-33] to decompose IBD prevalence, incidence, DALYs, and mortality by ageing structure, population growth, and epidemiological changes. The formula was as follows (e.g. prevalence):



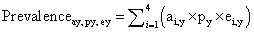


Ay, py, ey presented prevalence based on the factors of ageing structure, population growth, and prevalence rate for a specific year, respectively. Ai,y represents the population proportion for one of the four age categories in the year y. Py represents the total population in a given year y, and Ei,y represents the prevalence rate given age category one in year y. From 1990 to 2016, each factor’s contribution to the change in prevalence was defined by the effect of one factor changing while the others remained constant. To specify the effects of gender, we decompose gender into subgroups.

Further, we examined the relationship between age-standardised prevalence, incidence, DALYs, mortality rates, and HAQ to develop an understanding of the distribution of IBD burden on countries’ health system performance. Next, we applied frontier analysis [33] as a quantitative method to determine the lowest potentially achievable age-standardised prevalence, incidence, DALYs, and mortality rates based on development as measured by the SDI to assess the relationship between IBD burden and sociodemographic development.

All analyses were performed using R software, version 4.2.2 (the R Foundation for Statistical Computing, Vienna, Austria) and the Joinpoint Regression Program (version 4.9.1.0, New York, USA) [34]. A two-sided *P*-value <0.05 was considered statistically significant.

## RESULTS

### Prevalence rate of IBD (1990-2019)

**Figure 1:**
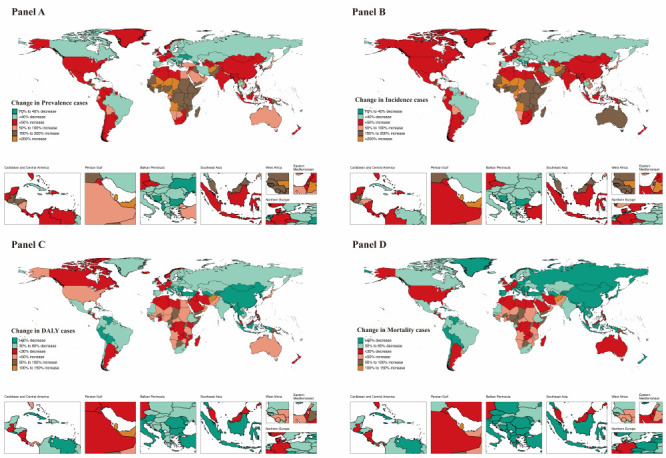
Geographical distribution of IBD prevalence, incidence, DALYs and mortality in children and adolescents in 204 countries and territories. **Panel A**. Worldwide prevalence of inflammatory bowel disease (IBD). **Panel B**. Worldwide incidence of IBD. **Panel C**. Worldwide DALYs of IBD. **Panel D**. Worldwide mortality of IBD.

Globally, the IBD prevalence increased between 1990 and 2019 (AAPC = 0.15 (95% CI = 0.11-0.19), and the prevalence of cases of IBD in children and adolescents increased from 74 976 in 1990 to 88 829 in 2019. In 2019, the global mean ASR estimates of IBD prevalence for all locations was 3.28 for a population per 100 000 (**Table 1**). Of the 21 regions, the highest prevalence was in high-income North America; the estimate was 26 per population of 100 000. The age-standardised prevalence rate (ASPR) of IBD increased with increasing age and increasing SDI values in 2019 (**Table 1**). At the national level, in 2019, the highest prevalence rate and most cases of IBD were in Canada (63.89%; 95% CI = 56.17, 72.39) and the USA (n = 20 884; 95% CI = 17 622, 24 674) (Table S1 in the **Online Supplementary Document**). In the past 30 years, the most pronounced increase in the prevalence rate of IBD was in Japan (AAPC = 3.23; 95% CI = 3.04, 3.42, *P* < 0.001), Republic of Korea (AAPC = 2.80; 95% CI = 2.35, 3.25, *P* < 0.001), Taiwan (Province of China) (AAPC = 2.79; 95% CI = 2.48, 3.10, *P* < 0.001) between 1990 and 2019. The most noteworthy decrease in the prevalence of IBD is in the Netherlands (AAPC = -4.50; 95% CI = -4.87, -4.13, *P* < 0.001) (Table S1 in the **Online Supplementary Document**). From 1990 to 2019, the number of prevalence cases increased by more than 200% in 11 countries, Equatorial Guinea (453.92%), Qatar (317.25%), Angola (249.62%), Afghanistan (240%), Jordan (238.01%), Cameroon (227.74%), Niger (224.47%), United Arab Emirates (222.35%), Chad (220.81%), Mali (209.02%), and Benin (208.09%). (**Figure 1**, Panel A, Table S2 in the **Online Supplementary Document**).

**Table 1:** Cases and age-standardised for IBD prevalence and their AAPCs from 1990 to 2019 at the global and regional levels

	1990		2019		1990-2019	
	**N (95% CI)**	ASPR (95% CI)*	**N (95% CI)**	ASPR (95% CI)*	**AAPCs (95% CI)**	***P*-value**
**Global**	74 975.52 (60 678.52, 92 219.2)	3.30 (2.66, 4.10)	88 828.9 (72 606.82, 108 301.34)	3.28 (2.66, 4.05)	0.15 (0.11, 0.19)	<0.001
**Sex**						
Male	36 174.71 (29 225.87, 44 415.14)	3.13 (2.51, 3.90)	43 931.61 (35 769.79, 53 651.22)	3.16 (2.56, 3.92)	0.23 (0.18, 0.28)	<0.001
Female	38 800.81 (31 524.56, 47 869.5)	3.47 (2.8, 4.32)	44 897.29 (36 811.36, 54 456.81)	3.41 (2.78, 4.18)	0.09 (0.04, 0.14)	0.001
**Age group in years**						
<5	135.76 (87.92, 194.56)	0.02 (0.01, 0.03)	149.36 (97.48, 214.13)	0.02 (0.01, 0.03)	0.16 (0.08, 0.24)	<0.001
5,9	3220.71 (2471.04, 4212.29)	0.55 (0.42, 0.72)	3639.31 (2777.4, 4796.2)	0.56 (0.42, 0.73)	0.05 (0.01, 0.1)	0.024
10,14	15 523.07 (12 408.44, 19 451.16)	2.89 (2.31, 3.62)	18 692.65 (14 979.24, 23 428.37)	2.91 (2.33, 3.65)	0.01 (-0.05, 0.07)	0.785
15,19	56 095.97 (45 464.7, 69 481.63)	10.80 (8.75, 13.37)	66 347.58 (54 242, 81 001.84)	10.71 (8.76, 13.07)	-0.04 (-0.12, 0.04)	0.024
**Sociodemographic index**						
High	37 811.69 (31 983.63, 45 212.93)	14.44 (12.15, 17.35)	45 514.8 (38 874.66, 53 318.49)	18.36 (15.51, 21.69)	0.84 (0.8, 0.88)	<0.001
High-middle	18 859.9 (14 963.91, 23 447.14)	4.32 (3.43, 5.41)	17 785.53 (14 403.58, 21 876.49)	5.00 (4.01, 6.22)	0.51 (0.43, 0.59)	<0.001
Middle	10 461.21 (7781.05, 13 980.57)	1.31 (0.95, 1.77)	12 757.24 (9511.44, 16 804.57)	1.62 (1.19, 2.16)	0.77 (0.59, 0.94)	<0.001
Low-middle	5830.01 (4231.33, 8038.19)	1.10 (0.79, 1.52)	8238.56 (5951.49, 11270.02)	1.11 (0.79, 1.52)	0.47 (0.35, 0.6)	<0.001
Low	1984.55 (1400.24, 2757.74)	0.79 (0.54, 1.13)	4499.68 (3171.91, 6268.95)	0.8 (0.55, 1.13)	0.40 (0.33, 0.47)	<0.001
**Region**						
Andean Latin America	178.94 (127.55, 244.33)	0.97 (0.68, 1.35)	257.70 (189.71, 349.92)	1.06 (0.76, 1.44)	0.51 (0.46, 0.56)	<0.001
Australasia	636.74 (495.4, 797.93)	8.91 (6.88, 11.25)	1127.67 (913.25, 1378.45)	14.9 (11.87, 18.48)	1.53 (1.44, 1.62)	<0.001
Caribbean	269.04 (199.37, 354.04)	1.71 (1.26, 2.28)	257.54 (188.06, 343.4)	1.56 (1.13, 2.1)	-0.22 (-0.31, -0.12)	<0.001
Central Asia	1137.48 (853.57, 1480.22)	3.87 (2.89, 5.08)	1271.79 (956.53, 1657.06)	3.99 (2.99, 5.27)	0.14 (-0.02, -0.3)	0.095
Central Europe	5549.48 (4475.4, 6869.89)	13.12 (10.54, 16.39)	4314.59 (3572.38, 5221.33)	17.09 (14.05, 20.86)	0.82 (0.72, 0.92)	<0.001
Central Latin America	1317.82 (976.32, 1746.68)	1.63 (1.19, 2.17)	1561.16 (1163.12, 2055.29)	1.65 (1.21, 2.2)	0.41 (0.34, 0.48)	<0.001
Central Sub-Saharan Africa	175.56 (116.41, 247.73)	0.66 (0.44, 0.95)	440.71 (301.79, 634.26)	0.67 (0.44, 0.98)	0.37 (0.35, 0.39)	<0.001
East Asia	6957.11 (5080.25, 9411.39)	1.31 (0.93, 1.78)	8510.98 (6410.74, 10 914.36)	2.56 (1.93, 3.33)	2.11 (1.89, 2.33)	<0.001
Eastern Europe	2726.01 (2071.56, 3484.74)	3.92 (2.97, 5.1)	1871.21 (1421.68, 2390.33)	4.03 (3.06, 5.22)	-0.08 (-0.35, -0.18)	0.532
Eastern Sub-Saharan Africa	543.83 (364.8, 769.45)	0.57 (0.38, 0.82)	1298.61 (867.87, 1844.43)	0.61 (0.4, 0.88)	0.58 (0.56, 0.59)	<0.001
High-income Asia Pacific	3889.16 (2995.37, 4969.33)	6.14 (4.72, 7.91)	6310.66 (5202.79, 7656.76)	16.55 (13.55, 20.28)	3.18 (2.95, 3.41)	<0.001
High-income North America	21632.66 (18227.26, 25833.71)	25.06 (20.99, 30.1)	26506.68 (22750.02, 30655.21)	25.99 (22.1, 30.41)	0.39 (0.31, 0.47)	<0.001
North Africa and Middle East	4041.79 (3081.45, 5196.44)	2.44 (1.85, 3.18)	6409.85 (4890.29, 8280.08)	2.75 (2.07, 3.62)	0.80 (0.69, 0.91)	<0.001
Oceania	14.59 (9.32,20.95)	0.47 (0.29, 0.69)	26.8 (17.79, 38.37)	0.47 (0.3, 0.68)	-0.01 (-0.07, -0.04)	0.625
South Asia	5173.08 (3662.34, 7387.12)	1.04 (0.72, 1.48)	7624.78 (5401.85, 10 797.63)	1.00 (0.69, 1.42)	0.55 (0.39, 0.7)	<0.001
Southeast Asia	1256.54 (837.39, 1746.79)	0.57 (0.37, 0.81)	1803.91 (1271.18, 2447.19)	0.74 (0.51, 1.02)	1.16 (1.05, 1.27)	<0.001
Southern Latin America	328.26 (245.24, 434.04)	1.67 (1.23, 2.25)	375.41 (280.33, 494.67)	1.73 (1.27, 2.33)	0.37 (0.24, 0.49)	<0.001
Southern Sub-Saharan Africa	194.86 (133.52, 274.43)	0.75 (0.5, 1.07)	245.87 (168.32, 348.63)	0.80 (0.54, 1.13)	0.27 (0.22, 0.32)	<0.001
Tropical Latin America	2642.7 (2067.82, 3362.95)	3.76 (2.91, 4.83)	2408.88 (1845.43, 3115.38)	3.28 (2.49, 4.26)	-0.12 (-0.21, -0.04)	0.006
Western Europe	15 732.64 (13 589.73, 18 495.67)	13.65 (11.68, 16.23)	14 597.96 (12 342.8, 17 461.53)	14.36 (12.02, 17.29)	-0.02 (-0.11, 0.07)	0.688
Western Sub-Saharan Africa	577.23 (389.05, 813.02)	0.63 (0.42, 0.91)	1606.14 (1103.88, 2256.27)	0.70 (0.47, 1)	0.64 (0.59, 0.69)	<0.001

AAPCs – average annual percent changes, ASPR – age-standardised prevalence rate, CI – confidence interval

*Per 100 000 population.

### Incidence rate of IBD (1990-2019)

Globally, incidence cases of IBD increased from 20 897.4 (95% CI = 17 008.6, 25 520.2) in 1990 to 25 658.6 (95% CI = 21 268.5, 31 075.6) in 2019, representing a 22.78% increase in incidence due to IBD over the last 30 years, and ASIR increased slightly from 0.92 per 100 000 population in 1990 to 0.95 per 100 000 population in 2019. Overall, the incidence of IBD increased (AAPC = 0.26; 95% CI = 0.22, 0.31) (Table S3 in the **Online Supplementary Document**). Considering SDI quintiles, with increasing SDI values, the ASIR of IBD increased in 2019, and high SDI increased notably (AAPC = 1.02; 95% CI = 0.97, 1.08) (Table S3 in the **Online Supplementary Document**).

As for geographical regions, the ASIR of IBD per 100 000 population was the highest in high-income North America (7.62; 95% CI = 6.47, 8.93), followed by high-income Asia Pacific (5.15; 95% CI = 4.14, 6.38), and Western Europe (5.01; 95% CI = 4.19, 5.97) (Table S3 in the **Online Supplementary Document**). On examining countries and territories, the USA, China, and Canada had the highest cases of incidence at 6042.1 (95% CI = 5148.4, 7114.5), 2140.5 (95% CI = 1633.9, 2740.2), 1616.49 (95% CI = 1505.8, 1719.0) (Table S4 in the **Online Supplementary Document**). Canada and Denmark had the highest ASIR at 18.56 (95% CI = 16.91, 20.21) and 11.14 (95% CI = 9.42, 13.06) in 2019 (Table S4 in the **Online Supplementary Document**). From 1990 until 2019, a primarily increased trend of the change for IBD incidence rate presents in Japan (AAPC = 3.82; 95% CI = 3.57, 4.07), the Republic of Korea (AAPC = 3.23; 95% CI = 2.85,3.61), and Taiwan (Province of China) (AAPC = 3.13; 95% CI = 2.82, 3.44). However, the IBD incidence trend decreased significantly in the Netherlands (AAPC = -4.41; 95% CI = -4.79, -4.04) (Table S4 in the **Online Supplementary Document**). It was noteworthy that compared to 1990, Equatorial Guinea (449.37%) and Qatar (329.29%) were the countries with the highest increase. However, the incidence cases decreased in only 26.47% of countries; the top three countries were the Netherlands (-71.75%), Georgia (-52.06%), and Bosnia and Herzegovina (-43.67%). (**Figure 1**, Panel B, Table S2 in the **Online Supplementary Document**).

### DALYs rate of IBD (1990-2019)

The IBD DALYs decreased between 1990 and 2019 (AAPC = -3.02; 95% CI = -3.15, -2.89), and the DALYs of cases of IBD in children and adolescents decreased from 24 3081 in 1990 to 11 3120 in 2019. In 2019, the global mean ASR estimates of IBD DALYs for all locations was 4.45 for a population of 100 000 (**Table 2**). Of the 21 regions, the highest DALYs were in the Caribbean; the estimate was 9.12 per population of 100 000 in 2019 (**Table 2**).

**Table 2:** Cases and age-standardised for IBD DALYs and their AAPCs from 1990 to 2019 at the global and regional levels

	1990		2019		1990-2019	
	**N (95% CI)**	ASDR (95% CI)*	**N (95% CI)**	ASDR (95% CI)*	**AAPCs (95% CI)**	***P*-value**
**Global**	243 081.06 (108 233.77, 388 783.07)	10.48 (4.52, 17.1)	113 119.86 (77 897.11, 150 499.13)	4.45 (2.9, 6.13)	-3.02 (-3.15, -2.89)	<0.001
**Sex**						
Male	99 500.5 (41 331.13, 186 870.36)	8.37 (3.37, 16)	53 517.94 (36 777.75, 72 745.97)	4.08 (2.64, 5.92)	-2.57 (-2.8, -2.35)	<0.001
Female	143 580.56 (49 892.18, 216 222.71)	12.71 (4.33, 19.58)	59 601.92 (34 932.52, 93 667.43)	4.83 (2.71, 7.87)	-3.37 (-3.48, -3.27)	<0.001
**Age group (years)**						
<5	180 191.31 (63 659.3, 313 186.52)	28.51 (10.07, 49.55)	61 670.73 (34 713.33, 90 780.17)	9.30 (5.24, 13.7)	-3.78 (-4.01, -3.55)	<0.001
5-9	21 610.9 (11 821.12, 31 572.89)	3.69 (2.02, 5.4)	12 126.73 (8717.56, 16 052.45)	1.85 (1.33, 2.45)	-2.39 (-2.6, -2.17)	<0.001
10-14	14 200.92 (9729.18, 18 012.14)	2.65 (1.81, 3.36)	12 142.77 (9426.53, 14 939.19)	1.89 (1.47, 2.33)	-1.13 (-1.31, -0.94)	<0.001
15-19	27 077.93 (19 123.35, 34 568.05)	5.21 (3.68, 6.65)	27 179.63 (21 373.86, 33 755.37)	4.39 (3.45, 5.45)	-0.60 (-0.69, -0.5)	<0.001
**Sociodemographic index**						
High	16 237.73 (13 655.35, 19 224.4)	6.84 (5.47, 8.33)	13 884.20 (11 068.85, 17 332.7)	6.06 (4.53, 7.69)	-0.32 (-0.44, -0.19)	<0.001
High-middle	36 394.31 (22 269.48, 50 209.97)	9.18 (5.39, 13.46)	10 772.62 (8626.89, 13 056.56)	3.28 (2.48, 4.22)	-3.40 (-3.58, -3.22)	<0.001
Middle	83 283.96 (40 873.99, 12 2053.8)	10.94 (5.09, 16.48)	20 473.36 (16 850.3, 24 199.47)	2.84 (2.22, 3.47)	-4.66 (-5.09, -4.23)	<0.001
Low-middle	66 462.32 (18 915.53, 121 875.63)	11.02 (3.07, 20.34)	27 170.82 (16 776.78, 38 601.05)	4.00 (2.35, 5.91)	-3.74 (-3.86, -3.63)	<0.001
Low	40 564.93 (6421.78, 90 744.58)	11.95 (2.03, 26.74)	40 716.69 (17 367.47, 67 289.41)	6.60 (2.7, 11.33)	-2.40 (-2.6, -2.2)	<0.001
**Region**						
Andean Latin America	7638.91 (2927.23, 13 933.81)	38.21 (13.46, 72.1)	1075.31 (704.24, 1618.28)	4.56 (2.59, 7.56)	-7.37 (-7.74, -6.99)	<0.001
Australasia	262.3 (198.17, 335.74)	4.08 (2.64, 5.79)	306.09 (207.02, 420.78)	4.17 (2.36, 6.39)	0.09 (-0.08, 0.27)	0.293
Caribbean	2277.7 (596.01, 5956.84)	14.91 (3.62, 40.42)	1364.42 (437.08, 3266.29)	9.12 (2.48, 23.02)	-1.89 (-2.11, -1.67)	<0.001
Central Asia	4275.66 (2607.5, 6598.99)	12.59 (7.17, 19.69)	2045.31 (1392.03, 2762.03)	5.92 (3.82, 8.61)	-2.55 (-2.83, -2.26)	<0.001
Central Europe	6519.94 (3786.91, 8350.97)	18.56 (9.64, 25.03)	1566.89 (1206.01, 1959.36)	6.66 (4.57, 9.03)	-3.11 (-3.48, -2.74)	<0.001
Central Latin America	4880.60 (3803.52, 6005.94)	5.83 (4.37, 7.45)	3129.39 (2344.15, 4013.01)	3.64 (2.6, 4.82)	-1.58 (-1.81, -1.34)	<0.001
Central Sub-Saharan Africa	4468.73 (953.77, 12131.76)	12.01 (2.51, 33.38)	4431.01 (1736.93, 9497.95)	6.03 (2.11, 13.43)	-2.78 (-2.92, -2.65)	<0.001
East Asia	94 770.7 (40 182.1, 14 6382.61)	21.23 (8.71, 33.72)	12 734.81 (9226.31, 16 247.23)	4.08 (2.84, 5.47)	-5.61 (-6.02, -5.20)	<0.001
Eastern Europe	2416.68 (1974.53, 3237.18)	3.63 (2.77, 5.3)	1153.46 (926.26, 1521.21)	2.48 (1.82, 3.63)	-1.28 (-1.4, -1.17)	<0.001
Eastern Sub-Saharan Africa	20 213.15 (3238.55, 50 686.4)	15.75 (2.68, 39.71)	18 154.07 (8579.00, 32 002.6)	7.83 (3.46, 13.99)	-2.78 (-2.95, -2.62)	<0.001
High-income Asia Pacific	1939.23 (1437.61, 2423.32)	3.68 (2.47, 5.23)	1138.10 (757.31, 1607.45)	3.05 (1.97, 4.53)	-0.36 (-0.45, -0.27)	<0.001
High-income North America	7794.70 (6392.28, 9518.25)	9.39 (7.34, 12.03)	8184.51 (6562.36, 10 189.88)	8.78 (6.65, 11.19)	-0.12 (-0.35, 0.11)	0.296
North Africa and Middle East	7272.85 (3324.73, 13 044.45)	3.94 (1.64, 8)	4945.44 (3784.57, 6451.98)	2.17 (1.5, 3.1)	-1.98 (-2.11, -1.85)	<0.001
Oceania	343.95 (105.41, 815.98)	9.62 (2.79, 23.19)	540.07 (212.62, 1146.92)	8.05 (2.98, 17.76)	-0.58 (-1.38, 0.23)	0.16
South Asia	38 436.87 (6980.67, 78 933.81)	6.75 (1.24, 14.04)	19 901.14 (9918.24, 30 443.36)	2.91 (1.35, 4.77)	-3.13(-3.38, -2.88)	<0.001
Southeast Asia	12 335.74 (4839.75, 23 211.5)	5.59 (2.08, 10.87)	4777.88 (3465.47, 6092.1)	2.16 (1.45, 2.99)	-3.29(-3.38, -3.19)	<0.001
Southern Latin America	380.89 (298.88, 446.82)	1.96 (1.43, 2.56)	270.09 (222.14, 324.82)	1.30 (0.97, 1.71)	-1.13(-1.28, -0.97)	<0.001
Southern Sub-Saharan Africa	2638.46 (900.86, 5550.51)	9.96 (3.09, 21.58)	1734.45 (1006.24, 2945.31)	5.74 (2.99, 10.21)	-1.98(-2.55, -1.4)	<0.001
Tropical Latin America	3480.49 (2863.05, 4341.64)	5.17 (3.96, 6.91)	2242.55 (1853.28, 2667.6)	3.35 (2.6, 4.23)	-1.30(-1.55, -1.05)	<0.001
Western Europe	7394.63 (6142.84, 8795.46)	7.53 (5.69, 9.51)	4873.71 (3839.27, 6052.6)	5.17 (3.61, 6.65)	-1.26(-1.44, -1.07)	<0.001
Western Sub-Saharan Africa	13 338.88 (1921.26, 27 461.25)	10.74 (1.7, 22.46)	18 551.17 (5986.05, 32 336.35)	7.15 (2.28, 12.89)	-1.81(-2.33, -1.3)	<0.001

AAPCs – average annual percent changes, ASDR – age-standardised DALYs rate, CI – confidence interval

*Per 100 000 population.

At the national level, the highest DALY rate and most cases of IBD were in Albania (38.3%; 95% CI = 16.5-71.5) and China (n = 12 328.5; 95% CI = 8911.5, 15 755.0) in 2019 (Table S5 in the **Online Supplementary Document**). In the past 30 years, the most pronounced decrease in the DALYs rate of IBD was in Peru (AAPC = -8.89; 95% CI = -9.49, -8.29, *P* < 0.001), El Salvador (AAPC = -7.53; 95% CI = -8.07, -6.99, *P* < 0.001), Mongolia (AAPC = -6.03; 95% CI = -6.43, -5.64, *P* < 0.001) between 1990 and 2019. The most noteworthy increase in the DALYs of IBD is Mauritius (AAPC = 3.09; 95% CI = 2.11, 4.09, *P* < 0.001) (Table S5 in the **Online Supplementary Document**). From 1990 to 2019, the number of DALYs cases increased by more than 100% in three countries: Qatar (131.53%), Afghanistan (131.12%), United Arab Emirates (102.29%) (**Figure 1**, Panel C, Table S2 in the **Online Supplementary Document**).

### The mortality rate of IBD (1990-2019)

Mortality cases of IBD decreased from 2756.5 (95% CI = 1162.6, 4484.9) in 1990 to 1208.0 (95% CI = 802.4, 1651.4) in 2019, representing a 56.17% decrease in mortality due to IBD over the last 30 years, and ASMR decreased from 0.12 per 100 000 population in 1990 to 0.05 per 100 000 population in 2019. Overall, the IBD mortality decreased (AAPC = -3.23; 95% CI = -3.32, -3.14) (Table S6 in the **Online Supplementary Document**). Considering the age group, <5 years old had the highest ASMR (0.11; 95% CI = 0.06, 0.16), with a decreased notably (AAPC = -3.78; 95% CI = -4.01, -3.56) (Table S6 in the **Online Supplementary Document**).

Of the 21 regions, a largely decreased trend of the change for IBD mortality rate presents in Andean Latin America (AAPC = -7.37; 95% CI = -7.75, -6.99), followed by high-income Asia Pacific (AAPC = -6.01; 95% CI = -6.38, -5.64), East Asia (AAPC = -5.97; 95% CI = -6.43, -5.50) (Table S6 in the **Online Supplementary Document**). On examining countries and territories, India, China, and Nigeria had the highest cases of mortality at 141.6 (95% CI = 61.3, 224.9), 130.8 (95% CI = 91.5, 171.1), and 116.0 (95% CI = 38.6, 214.3) (Table S7 in the **Online Supplementary Document**).

From 1990 until 2019, a largely decreased trend of the change for IBD mortality rate presents in Peru (AAPC = -8.88; 95% CI = -9.49, -8.26), El Salvador (AAPC = -8.22; 95% CI = -8.65, -7.79), Republic of Korea (AAPC = -7.94; 95% CI = -8.26, -7.61). However, the IBD mortality trend increased significantly in Mauritius (AAPC = 3.55; 95% CI = 2.4, 4.72) (Table S7 in the **Online Supplementary Document**). It was noteworthy that compared to 1990, the countries with the highest increase were Afghanistan (123.89%) and Qatar (111.75%). Encouragingly, the mortality cases decreased in 80.39% of countries; the top three countries were the Republic of Korea (-95.57%), El Salvador (-92.81%), and Albania (-92.62%) (**Figure 1**, Panel D, Table S2 in the **Online Supplementary Document**).

### Drivers of IBD epidemiology – ageing, population growth, and epidemiological change

**Figure 2:**
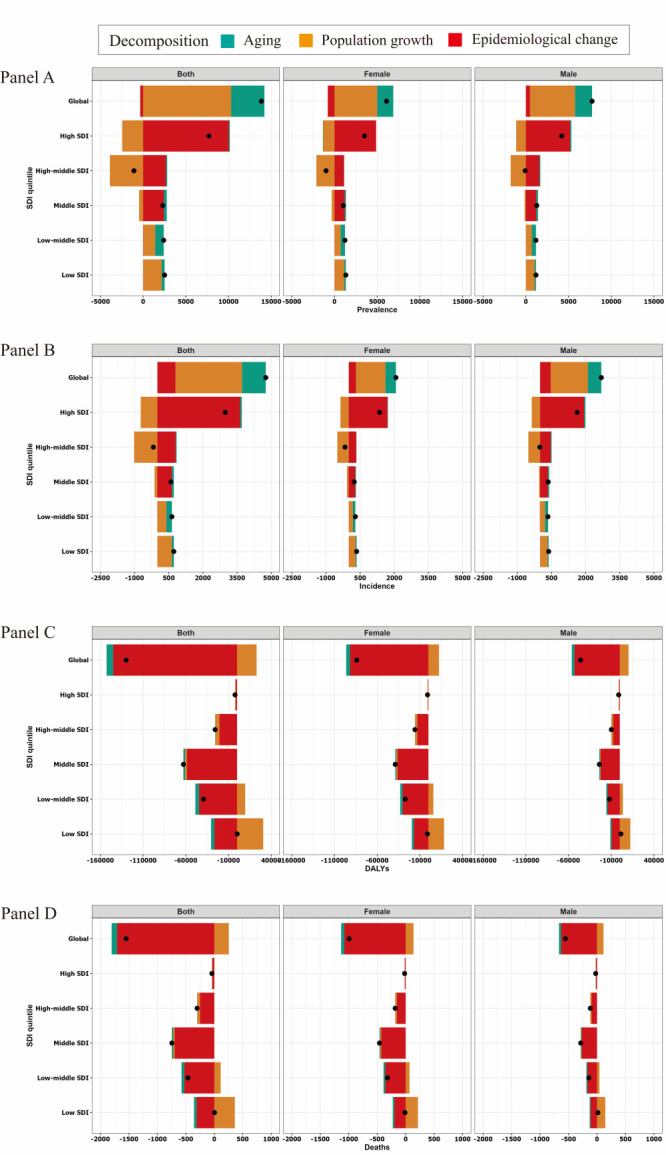
Changes in IBD prevalence, incidence, DALYs and mortality according to population-level determinants of ageing, population growth, and epidemiological change from 1990 to 2019 at the global level and by SDI quintile. **Panel A**. Prevalence. **Panel B**. Incidence. **Panel C**. Disability-adjusted life-years (DALYs). **Panel D**. Mortality. The black dot represents the overall value of change contributed by all 3 components. For each component, the magnitude of a positive value indicates a corresponding increase in IBD attributed to the component; the magnitude of a negative value indicates a corresponding decrease in IBD attributed to the related component.

We developed a decomposition analysis of raw prevalence, incidence, DALYs, and mortality age-standardised rates to evaluate to what extent the forces of ageing, population growth, and epidemiological changes have shaped IBD epidemiology over the past 30 years. Globally, IBD prevalence and incidence increased significantly, and the largest increased group was the high SDI quintile in the past 30 years. However, it was most pronounced in the high-middle SDI, where the prevalence and incidence decreased (**Figure 2**, Panel A-B). IBD prevalence burden decreased by 16.44% following population growth and increased by 6.09% with the ageing of the world population between 1990 and 2019 (**Table 3**). Most of the increase in IBD prevalence was driven by population growth in the low SDI quintile (8518.85%); the relative contribution of population growth to the increase in IBD prevalence was smaller (3.17%) in middle SDI countries (**Table 3**).

**Table 3:** Age-standardised rate for IBD prevalence, incidence, DALYs and mortality, and percentage change from 1990 globally and by SDI quintile

	Prevalence changes due to population-level determinants*			Incidence changes due to population-level determinants*			DALYs changes due to population-level determinants*			Mortality changes due to population-level determinants*		
	**Ageing**	**Population**	**EC**	**Ageing**	**Population**	**EC**	**Ageing**	**Population**	**EC**	**Ageing**	**Population**	**EC**
**Global**	-94.35 (6.09%)	254.6 (-16.44%)	-1708.67 (110.35%)	1043.61 (21.92%)	2924.67 (61.43%)	792.85 (16.65%)	-7720.96 (5.94%)	22 846.59 (-17.58%)	-145 086.83 (111.64%)	-94.35 (6.09%)	254.6 (-16.44%)	-1708.67 (110.35%)
**High SDI**	-1.64 (3.93%)	-6.02 (14.42%)	-34.08 (81.65%)	71.51 (2.4%)	-731.41 (-24.56%)	3637.82 (122.16%)	-126.75 (5.39%)	-880.84 (37.43%)	-1345.94 (57.19%)	-1.64 (3.93%)	-6.02 (14.42%)	-34.08 (81.65%)
**High-middle SDI**	-3.39 (1.12%)	-49.28 (16.33%)	-249.09 (82.55%)	37.83 (-21.58%)	-1020.67 (582.24%)	807.54 (-460.66%)	-274.5 (1.07%)	-4725.94 (18.45%)	-20 621.24 (80.48%)	-3.39 (1.12%)	-49.28 (16.33%)	-249.09 (82.55%)
**Middle SDI**	-24.31 (3.26%)	-23.66 (3.17%)	-697.39 (93.56%)	87.64 (14.68%)	-124.9 (-20.92%)	634.35 (106.24%)	-2075.87 (3.3%)	-2050.07 (3.26%)	-58 684.66 (93.43%)	-24.31 (3.26%)	-23.66 (3.17%)	-697.39 (93.56%)
**Low-middle SDI**	-49.05 (10.58%)	112.07 (-24.19%)	-526.4 (113.6%)	245.78 (38.58%)	393.62 (61.79%)	-2.41 (-0.38%)	-4294.91 (10.93%)	9499.73 (-24.18%)	-44 496.33 (113.25%)	-49.05 (10.58%)	112.07 (-24.19%)	-526.4 (113.6%)
**Low SDI**	-45.51 (-1074.88%)	360.68 (8518.85%)	-310.94 (-7343.96%)	87.68 (12.14%)	632.08 (87.5%)	2.64 (0.37%)	-3969.74 (-2615.77%)	30 637.89 (20 188.17%)	-26 516.39 (-17 472.39%)	-45.51 (-10 74.88%)	360.68 (8518.85%)	-310.94 (-7343.96%)

DALY – disability-adjusted life years, EC – Epidemiological change, SDI – sociodemographic index

*Percentages contribute to the total changes.

Overall, there was a significant increase in IBD incidence globally and in high SDI, middle SDI, low-middle SDI, and low SDI quintiles in the past 30 years. (**Figure 2**, Panel B). Globally, population growth followed by the ageing of the world population contributed 61.43% and 21.92%, respectively, to the increased burden of IBD incidence between 1990 and 2019. Most of the increase in IBD incidence was driven by population growth in the high-middle SDI quintile (582.24%) (**Table 3**). The contribution of ageing to overall incidence was most pronounced in the low-middle SDI quintile (38.58%), followed by middle SDI (14.68%), low SDI (12.14%), high SDI (2.40%), and decreased in the high-middle SDI (-21.58%) (**Table 3**).

Notably, a trend exhibited a decrease in underlying age and population-adjusted IBD DALYs and mortality rates, seen at the global and SDI levels. The contribution of population growth to overall DALYs was most pronounced in the low SDI quintile (20 188.17%). In terms of mortality, the situation was the same; the low SDI quintile (8518.85%) contributed the most to overall mortality (**Table 3**). The epidemiologic change that captures the underlying change in age and population-adjusted IBD DALYs and mortality rates over the past 30 years has decreased globally, and the decrease was more evident in middle SDI, low-middle SDI, low SDI, and high-middle quintiles, and was least pronounced in high SDI quintile (**Figure 2**, Panel C-D, **Table 3**).

### IBD and sociodemographic development

**Figure 3:**
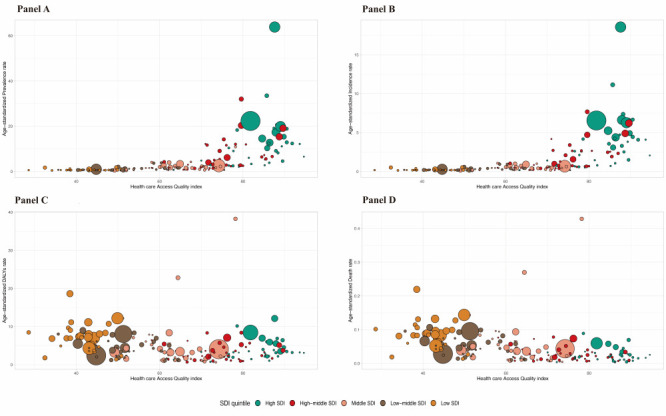
Association between age-standardised IBD prevalence, incidence, DALYs and mortality rate and HAQ index. Each circle represents a country. Circles are colored according to SDI quintile. Circle size corresponds to population number.

Countries with high HAQ had relatively higher IBD ASPR and ASIR, whereas countries with low HAQ had much lower ASPR and ASIR (**Figure 3**, Panel A-B). On the contrary, countries with high HAQ had relatively lower ASDR and ASMR. (**Figure 3**, Panel C-D). These findings suggest that the burden of IBD is disproportionately higher and is borne by countries least equipped to cope.

### Frontier analysis for the relationship between IBD DALYs and the status of the countries’ development

**Figure 4:**
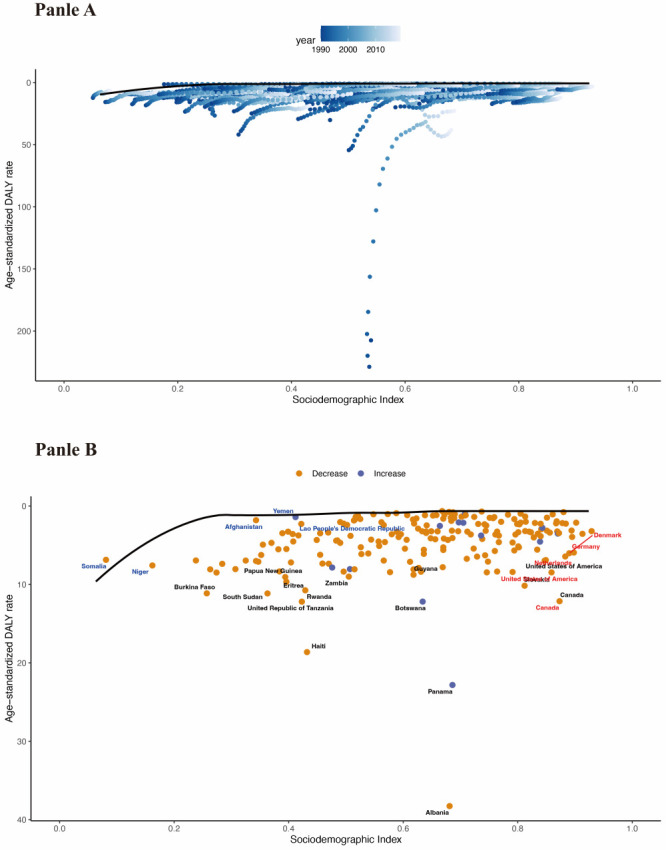
Frontier analysis based on SDI and DALY of IBD in children and adolescents in 204 countries and territories. **Panel A**. Frontier analysis based on SDI and age-standardised IBD DALY rate from 1990 to 2019. Color scale represents the years from 1990 depicted in blue to 2019 depicted in gray. Solid black color to delineate the frontier. **Panel B**. Frontier analysis based on SDI and age-standardised IBD DALY rate in 2019. Dots represent countries and territories. The frontier is delineated in solid black color. Black fonts are used to label the top 15 countries with the largest effective difference (largest IBD DALY gap from the frontier). Examples of countries and territories with high SDI (>0.85) and relatively high effective difference for their level of development are labeled in red (e.g. USA, Norway, Japan, Denmark, and Canada), and blue fonts are used to label examples of frontier countries with low SDI (<0.5) and low effective difference (e.g. Somalia, Burundi, Vanuatu, Papua New Guinea, Solomon Islands). Red dots indicate a decrease in age-standardised IBD DALY rate from 1990 to 2019; blue dots indicate an increase in age-standardised IBD DALY rate from 1990 to 2019.

We used the 2019 DALYs and SDI to calculate the effective difference from the frontier for each country and territory (**Figure 4**, Panel B). Overall, as SDI increases, the effective difference in SDI tends to be smaller and the variance smaller. The top 15 countries with the highest effective difference from the frontier included Albania, Panama, Haiti, the United Republic of Tanzania, Botswana, Canada, Rwanda, South Sudan, Burkina Faso, Eritrea, Slovakia, Papua New Guinea, Zambia, Guyana, United States of America. These countries have much higher IBD DALYs rates than other countries with comparable sociodemographic resources (**Figure 4**, Panel B).

## DISCUSSION

We developed a comprehensive and detailed description of the prevalence, incidence, DALYs, and mortality of IBD among children and adolescents aged 0-19 from 1990 to 2019 according to age group and SDI quintiles. We showed that the burden of IBD measured in prevalence and incidence increased substantially, and in DALYs due to IBD, mortality due to IBD decreased substantially. Globally, in 2019 there were 88 829 prevalent cases of IBD per year (an increase of 18.48%), 25 659 incidence cases (an increase of 22.78%), 113 120 DALYs due to IBD (a decrease of 53.46%), and 1208 deaths due to IBD (a decrease of 56.17%). Geography and level of development play a crucial role in the changes in IBD burden. For instance, the highest prevalence rate of IBD was in high-income North America, while the lowest incidence rate was observed in Southern Sub-Saharan Africa. The wide geographical differences may be correlated with several factors, including race, environmental exposures in society, urbanisation, and diets low in dietary fiber and high in meat [35-39]. These findings can inform intervention targets in different regions in the future. We report an increase in ASPR and ASIR of IBD in regions that previously had low prevalence and incidence, including East and South Asia and sub-Saharan Africa, similar to the findings of a previous whole age group GBD study (1990-2019) [20].

Factors such as improved socioeconomic conditions, changes in diet and other lifestyles, improved hygiene, changes in microbiota, and environmental factors in newly industrialised countries may increase the risk of developing IBD [40-42]. Indeed, in such Asian and African countries with large population bases where ASPR and ASIR of IBD are increasing, new biologic agents are also widely used. This poses significant practical challenges to global public health policy. Policies are needed in stable IBD prevalence regions with high SDI to provide the best platform to support biologics research and development. In areas with rapidly rising prevalence and poor health care resources, proper diagnosis and treatment are needed to access health care services and adequate health care facilities. Improving the health care system, making diagnostic tools more widely available, and raising awareness among patients and doctors may also help increase the diagnosis rate.

The prevalence and incidence of IBD among children and adolescents continue to rise in areas where it was previously low, with important implications for both health care providers and those responsible for health policy planning. Children and adolescents with IBD are associated with reduced participation in extracurricular activities, days missed from school, subjective poor school performance, and decreased quality of life [43,44][REMOVED REF FIELD]; and the meantime, school accommodations conditions should be considered early, which are different from adults with IBD.

The decomposition showed that the IBD prevalence and incidence largest increased group was the High SDI quintile region in the past 30 years. It also makes sense that higher SDI regions have a higher prevalence due to access to better access to diagnostic testing tools [39]. However, the low SDI quintile region was the largest contribution to overall DALYs and mortality. Globally, IBD prevalence and incidence were increased (AAPC>0), but DALYs and mortality were decreased (AAPC<0). While mortality rates for chronic diseases such as IBD are declining, the slight increase in prevalence in low SDI regions is having a devastating impact on developing countries, which are home to almost three-quarters of the world’s population [45]. It is important to note that the impact of IBD on public health is not limited to its burden on the health care systems. As the population ages, the mortality rate of IBD is gradually decreasing, and the survival rate is improving, so is the cost of paying for children and adolescents to receive rehabilitation treatment. Our findings can motivate health planners to develop cost-effective and simple community-based interventions for primary care professionals to implement.

We found that the burden of IBD is more heavily skewed toward less economically developed countries. There was also a negative association between ASDR and ASMR rates and measures of HAQ. Our frontier analysis develops a more optimistic assessment despite the results highlighting a significant challenge, as there are few countries at all levels of the development spectrum with IBD DALYs and mortality that are distant from the frontier delineation line. While there are frontier countries at all SDI levels, those with lower SDI are most notable for showing leading performance despite limited resources. These countries can serve as exemplary countries for optimising health outcomes in low-resource settings.

Several limitations should be mentioned. First, this study is a macro assessment of global, regional, and national epidemiological trends [19,20] in IBD among children and adolescents, so it may not capture micro-level trends that may be particularly relevant in large regions [46] (e.g. India, China, Canada, and the US). Second, we did not distinguish IBD categories, CD or UC, which has considerable implications for assessing DALYs and mortality risk. Third, while GBD 2019 has used all available data to the extent possible, many countries in low- and middle-income countries do not yet have access to these data. In addition, differences and inconsistencies in data collection tools and methods across time periods and countries may affect temporal trends and geographic differences. Furthermore, GBD estimates were derived by mathematical modeling for those countries with limited data [47].

Our study identified several key advantages, including utilising the availability of GBD data from 1990 to 2019, as the GBD database is the most comprehensive compilation and analysis of available global health information. We developed a decomposition analysis to illuminate the impact of demographic trends on the burden of IBD among children and adolescents over the past 30 years, and finally, we developed a frontier analysis for comparative assessment between countries with similar SDI.

## CONCLUSIONS

In conclusion, the global prevalence and incidence rate of IBD among children and adolescents has been increasing from 1990 to 2019, while the DALYs and mortality have been decreasing. Rising prevalence and rising incidence in areas with historically low rates will have crucial health and economic implications. Our findings could raise awareness among doctors and patients and improve the health care system to increase the rate of diagnosis for responding to the growing number of children with IBD. We emphasise that understanding the common and different environmental determinants of IBD in different regions is critical to implementing interventions to mitigate the growing global burden of IBD.

## Additional material


Online Supplementary Document
