# Supplementary material for: Epidemiological analysis reveals a surge in inflammatory bowel disease among children and adolescents: A global, regional, and national perspective from 1990 to 2019 – insights from the China study
Source: J Glob Health. 2023 Dec 1;13:04174. doi: 10.7189/jogh.13.04174 (PMC10690112; doi:10.7189/jogh.13.04174)
Supplement: Online Supplementary Document [file jogh-13-04174-s001.pdf]

Supplementary table

Supplementary table 1. Cases and age-standardized for IBD prevalence and their average annual percentage changes (AAPCs) from 1990 to 2019 at the national levels.

	1990		2019		AAPCs, 1990-2019 (95% CI)	P value
	cases (n)	ASPR (per 100,000 population, 95% CI)	cases (n)	ASPR (per 100,000 population, 95% CI)		
Afghanistan	98.54 (71.8-135.06)	1.62 (1.15-2.23)	335.27 (245.3-460.21)	1.71 (1.22-2.38)	-0.03 (-0.25 to 0.19)	0.774
Albania	152.43 (118.36-196.82)	10.42 (8.02-13.56)	87.1 (67.1-111.33)	10.91 (8.38-14.01)	0.83 (0.69 to 0.97)	<0.001
Algeria	317.12 (239.3-407.13)	2.5 (1.86-3.25)	340.65 (252.55-460.63)	2.48 (1.82-3.37)	-0.16 (-0.4 to 0.07)	0.173
American Samoa	0.14 (0.09-0.2)	0.65 (0.42-0.94)	0.18 (0.12-0.25)	0.69 (0.45-0.99)	0.93 (0.87 to 0.99)	<0.001
Andorra	1.14 (0.86-1.46)	6.81 (5.1-8.87)	1.19 (0.91-1.54)	6.56 (4.99-8.55)	-0.21 (-0.31 to -0.11)	<0.001
Angola	30.16 (20.22-43.12)	0.62 (0.4-0.89)	105.45 (72.29-147.08)	0.72 (0.47-1.02)	0.59 (0.5 to 0.67)	<0.001
Antigua and Barbuda	0.45 (0.33-0.6)	1.8 (1.31-2.43)	0.52 (0.38-0.71)	1.82 (1.32-2.5)	0.61 (0.55 to 0.68)	<0.001
Argentina	170.15 (122.4-231.26)	1.31 (0.93-1.8)	208.7 (150.8-279.15)	1.36 (0.97-1.86)	0.38 (0.33 to 0.44)	<0.001
Armenia	49.96 (37.61-64.42)	3.95 (2.95-5.14)	31.7 (24.1-41.49)	4.24 (3.17-5.58)	0.28 (0.01 to 0.55)	0.04
Australia	508.82 (392.04-637.17)	8.67 (6.65-10.94)	967.87 (776.81-1190.27)	15.38 (12.15-19.12)	1.74 (1.61 to 1.87)	<0.001
Austria	521.87 (428.19-631.53)	23.78 (19.22-29.02)	398.88 (333.6-483.13)	20.55 (16.84-25.16)	-0.53 (-0.88 to -0.18)	0.003
Azerbaijan	119.9 (90.54-156.84)	3.9 (2.91-5.16)	122.24 (92.82-159.09)	4.09 (3.08-5.35)	0.26 (0.07 to 0.44)	0.006
Bahamas	2.26 (1.65-3.02)	1.93 (1.4-2.61)	2.4 (1.73-3.26)	1.82 (1.3-2.51)	0.08 (-0.11 to 0.26)	0.421
Bahrain	4.9 (3.72-6.27)	2.85 (2.15-3.7)	10.27 (7.67-13.53)	2.8 (2.06-3.76)	1.01 (0.68 to 1.34)	<0.001
Bangladesh	515.48 (362.71-729.83)	0.97 (0.67-1.39)	757.31 (537.8-1090.95)	1.15 (0.8-1.67)	1.36 (1.33 to 1.4)	<0.001

Barbados	3.01 (2.22-3.91)	3.12 (2.29-4.09)	2.51 (1.87-3.27)	2.99 (2.21-3.97)	0.11 (0.05 to 0.17)	0.001
Belarus	161.37 (123.85-206.11)	5 (3.82-6.5)	106.95 (81.52-137.59)	5.36 (4.07-6.99)	-0.01 (-0.18 to 0.17)	0.925
Belgium	297.79 (251.69-348.89)	10.61 (8.83-12.73)	249.72 (192.98-320.64)	9.13 (7.06-11.79)	-0.69 (-0.88 to -0.51)	<0.001
Belize	1.32 (0.96-1.79)	1.4 (0.99-1.92)	2.87 (2.09-3.89)	1.5 (1.08-2.06)	0.96 (0.91 to 1.02)	<0.001
Benin	13.83 (9.47-19.78)	0.62 (0.41-0.9)	42.61 (28.7-60.01)	0.68 (0.45-0.98)	0.73 (0.62 to 0.85)	<0.001
Bermuda	0.33 (0.25-0.44)	1.97 (1.44-2.64)	0.27 (0.2-0.36)	2 (1.45-2.7)	0.23 (0.12 to 0.33)	<0.001
Bhutan	3.19 (2.2-4.59)	1 (0.67-1.46)	3.61 (2.51-5.13)	1.21 (0.83-1.76)	1.16 (1.09 to 1.23)	<0.001
Bolivia (Plurinational State of)	26.19 (18.2-36.71)	0.86 (0.59-1.22)	41.09 (29.06-55.96)	0.87 (0.6-1.21)	0.21 (0.19 to 0.24)	<0.001
Bosnia and Herzegovina	171.65 (136.74-215.64)	10.23 (8.07-12.98)	95.38 (76.91-117.66)	12.09 (9.7-15.04)	0.76 (0.55 to 0.97)	<0.001
Botswana	5.18 (3.48-7.37)	0.75 (0.49-1.08)	8.34 (5.71-11.72)	0.89 (0.59-1.25)	0.8 (0.69 to 0.92)	<0.001
Brazil	2597.49 (2033.62-3300.71)	3.79 (2.94-4.87)	2351.71 (1806.86-3043.02)	3.33 (2.54-4.32)	-0.11 (-0.2 to -0.01)	0.028
Brunei Darussalam	4.83 (3.6-6.4)	4.47 (3.29-6)	7.36 (5.52-9.84)	4.83 (3.59-6.48)	1.02 (0.94 to 1.1)	<0.001
Bulgaria	304.65 (236.23-389.11)	11.25 (8.7-14.42)	162.69 (125.62-208.8)	11.68 (8.93-14.93)	-0.12 (-0.18 to -0.07)	<0.001
Burkina Faso	26.36 (17.12-38.24)	0.57 (0.36-0.84)	69.3 (45.65-98.95)	0.62 (0.4-0.91)	0.54 (0.49 to 0.58)	<0.001
Burundi	14.09 (9.21-20.43)	0.55 (0.35-0.81)	30.8 (20.43-44.14)	0.52 (0.33-0.77)	0.16 (0.08 to 0.24)	<0.001
Cabo Verde	1.34 (0.9-1.87)	0.78 (0.52-1.11)	1.97 (1.37-2.76)	0.9 (0.61-1.28)	1.00 (0.9 to 1.1)	<0.001
Cambodia	22.67 (14.42-32.2)	0.47 (0.29-0.68)	37.06 (24.5-53.46)	0.57 (0.36-0.83)	1.27 (1.15 to 1.38)	<0.001
Cameroon	34.59 (23.37-49.18)	0.7 (0.47-1)	113.38 (76.47-159.04)	0.77 (0.51-1.1)	0.81 (0.74 to 0.88)	<0.001
Canada	6017.97 (5419.05-6656.01)	73.42 (64.9-82.27)	5620.43 (5011.86-6319.31)	63.89 (56.17-72.39)	-0.4 (-0.49 to -0.3)	<0.001

Central African Republic	7.75 (5.19-11.1)	0.61 (0.39-0.88)	15.67 (10.33-22.27)	0.59 (0.38-0.86)	0.28 (0.22 to 0.34)	<0.001
Chad	16.27 (10.57-23.5)	0.57 (0.37-0.84)	52.18 (34.78-75.24)	0.62 (0.4-0.91)	0.37 (0.3 to 0.44)	<0.001
Chile	142.04 (106.95-185.29)	2.57 (1.91-3.41)	151.46 (113.46-197.12)	2.85 (2.11-3.76)	0.60 (0.39 to 0.8)	<0.001
China	6757.45 (4930.57-9142.18)	1.31 (0.93-1.79)	8284.84 (6242.36-10625.62)	2.59 (1.96-3.37)	2.12 (1.9 to 2.34)	<0.001
Colombia	229.69 (166.29-308.25)	1.54 (1.11-2.1)	262.22 (190.05-354.31)	1.57 (1.13-2.15)	0.50 (0.43 to 0.58)	<0.001
Comoros	1.49 (1-2.09)	0.63 (0.41-0.9)	2.11 (1.42-2.96)	0.67 (0.44-0.95)	0.70 (0.64 to 0.76)	<0.001
Congo	9.77 (6.73-13.79)	0.79 (0.53-1.12)	19.81 (13.54-27.88)	0.84 (0.56-1.2)	0.35 (0.24 to 0.46)	<0.001
Cook Islands	0.06 (0.04-0.08)	0.67 (0.44-0.97)	0.04 (0.03-0.06)	0.72 (0.48-1.03)	0.48 (0.38 to 0.58)	<0.001
Costa Rica	22.41 (16.45-29.97)	1.68 (1.21-2.27)	26.56 (19.42-35.02)	1.71 (1.25-2.29)	0.61 (0.53 to 0.68)	<0.001
Croatia	345.72 (298.87-398.45)	23.43 (20.12-27.17)	226.12 (187.9-268.02)	24.53 (20.17-29.4)	0.17 (0.04 to 0.31)	0.01
Cuba	78.55 (56.53-107.68)	1.75 (1.26-2.41)	48.3 (35.24-65.41)	1.7 (1.22-2.32)	-0.3 (-0.37 to -0.24)	<0.001
Cyprus	9.49 (7.22-12.34)	3.53 (2.65-4.63)	15.22 (12.32-18.95)	5.32 (4.26-6.72)	1.34 (1.22 to 1.45)	<0.001
Czechia	303.71 (243.87-379.18)	8.37 (6.67-10.5)	328.27 (270.73-402.13)	15.28 (12.47-18.76)	1.38 (1.18 to 1.58)	<0.001
C 么 te d'Ivoire	38.67 (25.88-54.38)	0.68 (0.44-0.98)	85.92 (58.27-121.99)	0.71 (0.47-1.02)	0.51 (0.45 to 0.57)	<0.001
Democratic People's Republic of Korea	103.09 (74.01-140.04)	1.31 (0.93-1.79)	99.47 (70.71-134.08)	1.32 (0.92-1.79)	0.63 (0.58 to 0.68)	<0.001
Democratic Republic of the Congo	122.98 (80.78-173.51)	0.67 (0.43-0.96)	285.92 (191.3-416.29)	0.65 (0.42-0.96)	0.31 (0.26 to 0.36)	<0.001
Denmark	494.77 (432.59-565.96)	32.1 (27.62-37.3)	494.77 (423.8-581.5)	33.52 (28.45-39.43)	-0.22 (-0.44 to 0)	0.054
Djibouti	1.57 (1.05-2.26)	0.63 (0.41-0.92)	3.48 (2.35-4.9)	0.73 (0.48-1.04)	0.53 (0.46 to 0.61)	<0.001

Dominica	0.58<br>(0.42-0.78)	1.69<br>(1.22-2.29)	0.4<br>(0.29-0.53)	1.65<br>(1.18-2.21)	0.38<br>(0.32 to 0.43)	<0.001
Dominican Republic	51.94<br>(37.53-71.22)	1.47<br>(1.04-2.05)	62.71<br>(45.01-85.67)	1.51<br>(1.06-2.07)	0.24<br>(0.15 to 0.32)	<0.001
Ecuador	50.08<br>(36.71-66.94)	1.02<br>(0.73-1.38)	95.42<br>(71.4-123.36)	1.34<br>(0.99-1.77)	1.21<br>(1.11 to 1.31)	<0.001
Egypt	743.5<br>(560.7-950.91)	2.89<br>(2.16-3.76)	1137.36<br>(838.37-1520.07)	2.74<br>(1.99-3.76)	0.05<br>(0 to 0.11)	0.071
El Salvador	36.15<br>(26.27-49.13)	1.35<br>(0.96-1.84)	35.41<br>(25.15-48.4)	1.43<br>(1.01-1.95)	0.51<br>(0.35 to 0.68)	<0.001
Equatorial Guinea	1.22<br>(0.8-1.72)	0.61<br>(0.39-0.89)	6.74<br>(4.73-9.32)	0.85<br>(0.58-1.2)	2.15<br>(2.08 to 2.22)	<0.001
Eritrea	8.89<br>(5.92-12.61)	0.59<br>(0.38-0.84)	21.46<br>(14.41-30.41)	0.64<br>(0.42-0.91)	0.72<br>(0.61 to 0.83)	<0.001
Estonia	25.74<br>(20.35-32.1)	5.38<br>(4.2-6.74)	16.77<br>(12.78-21.49)	6.05<br>(4.55-7.85)	0.28<br>(0.02 to 0.55)	0.038
Eswatini	3.03<br>(2.06-4.32)	0.71<br>(0.47-1.02)	4.21<br>(2.87-5.81)	0.78<br>(0.52-1.1)	0.74<br>(0.66 to 0.83)	<0.001
Ethiopia	127.98<br>(85.19-183.72)	0.51<br>(0.33-0.74)	314.91<br>(207.04-453.69)	0.56<br>(0.36-0.82)	0.79<br>(0.76 to 0.82)	<0.001
Fiji	1.88<br>(1.23-2.73)	0.54<br>(0.34-0.77)	1.97<br>(1.3-2.83)	0.57<br>(0.37-0.84)	0.32<br>(0.21 to 0.43)	<0.001
Finland	334.14<br>(289.13-381.69)	24.99<br>(21.17-29.05)	241.47<br>(194.92-303.38)	18.63<br>(14.87-23.63)	-0.74<br>(-1.08 to -0.4)	<0.001
France	3077.96<br>(2774.4-3440.97)	16.81<br>(14.88-19.05)	3106.59<br>(2585.03-3649.03)	17.38<br>(14.26-20.61)	0.20<br>(0.02 to 0.39)	0.028
Gabon	3.69<br>(2.51-5.17)	0.81<br>(0.54-1.14)	7.12<br>(4.86-10.04)	0.92<br>(0.61-1.3)	0.96<br>(0.92 to 0.99)	<0.001
Gambia	3.2<br>(2.08-4.46)	0.68<br>(0.43-0.97)	8.15<br>(5.53-11.7)	0.70<br>(0.46-1.02)	0.69<br>(0.64 to 0.74)	<0.001
Georgia	80.01<br>(60.87-104.29)	4.28<br>(3.22-5.62)	37.93<br>(28.67-49.19)	4.30<br>(3.2-5.66)	-0.27<br>(-0.42 to -0.13)	<0.001
Germany	1720.36<br>(1347.7-2235.06)	9.14<br>(7.1-11.94)	2243.62<br>(1803.18-2819.63)	12.76<br>(10.01-16.21)	1.27<br>(1.01 to 1.53)	<0.001
Ghana	56.11<br>(37.44-78.44)	0.78<br>(0.51-1.11)	126.26<br>(86.1-178.3)	0.87<br>(0.59-1.23)	0.88<br>(0.82 to 0.93)	<0.001
Greece	213.35<br>(164.86-274)	6.32<br>(4.87-8.2)	146.97<br>(116.83-183.73)	6.62<br>(5.15-8.36)	-0.15<br>(-0.31 to 0.01)	0.059
Greenland	1.78<br>(1.34-2.33)	10.95<br>(8.21-14.56)	1.92<br>(1.46-2.51)	12.21<br>(9.16-16.1)	0.83<br>(0.67 to 1)	<0.001

Grenada	0.6 (0.43-0.82)	1.51 (1.05-2.06)	0.57 (0.41-0.77)	1.57 (1.11-2.16)	0.77 (0.73 to 0.81)	<0.001
Guam	0.36 (0.24-0.52)	0.68 (0.45-0.99)	0.42 (0.29-0.59)	0.72 (0.48-1.02)	0.22 (0.14 to 0.29)	<0.001
Guatemala	46.56 (33.29-63.07)	1.22 (0.86-1.68)	108.31 (77.11-145.34)	1.33 (0.93-1.81)	1.00 (0.9 to 1.09)	<0.001
Guinea	16.41 (10.62-23.55)	0.61 (0.39-0.89)	40.26 (27.02-56.06)	0.65 (0.42-0.93)	0.58 (0.54 to 0.63)	<0.001
Guinea-Bissau	3.05 (2.04-4.34)	0.61 (0.4-0.88)	6.25 (4.21-8.98)	0.66 (0.44-0.97)	0.63 (0.54 to 0.72)	<0.001
Guyana	5.6 (4.05-7.52)	1.47 (1.04-2)	4.62 (3.37-6.27)	1.50 (1.06-2.06)	0.28 (0.09 to 0.48)	0.004
Haiti	35.73 (25.71-48.05)	1.22 (0.85-1.67)	67.82 (48.13-93.08)	1.25 (0.87-1.75)	0.47 (0.44 to 0.51)	<0.001
Honduras	30.6 (22.06-41.92)	1.29 (0.91-1.77)	61.44 (43.77-83.91)	1.34 (0.93-1.85)	0.75 (0.7 to 0.81)	<0.001
Hungary	769.67 (648.94-908.95)	22.65 (18.92-26.77)	674.78 (577.24-796.61)	32.03 (27.02-38.15)	1.02 (0.83 to 1.21)	<0.001
Iceland	8.46 (6.59-10.84)	9.29 (7.16-11.89)	11.48 (9.05-14.69)	12.17 (9.5-15.85)	0.86 (0.49 to 1.23)	<0.001
India	4113.85 (2910-5857.59)	1.08 (0.76-1.54)	5682.38 (4025.66-8029.36)	0.99 (0.68-1.41)	0.43 (0.24 to 0.63)	<0.001
Indonesia	402.73 (259.72-581.09)	0.46 (0.28-0.67)	531.22 (351.01-751.56)	0.54 (0.35-0.78)	0.94 (0.84 to 1.04)	<0.001
Iran (Islamic Republic of)	511.77 (369.8-708.9)	1.79 (1.28-2.51)	453.61 (329.67-627.78)	1.83 (1.31-2.56)	0.28 (-0.02 to 0.58)	0.07
Iraq	144.98 (107.27-193.18)	1.63 (1.18-2.21)	379.57 (273.77-515.37)	1.98 (1.41-2.74)	1.3 (1.19 to 1.41)	<0.001
Ireland	118.76 (93.87-151.05)	7.9 (6.2-10.12)	115.85 (89.65-149.11)	8.23 (6.31-10.72)	-0.13 (-0.34 to 0.08)	0.224
Israel	123.7 (97.78-156.41)	5.96 (4.66-7.62)	178.67 (142.72-224.59)	5.57 (4.35-7.11)	-0.44 (-0.61 to -0.26)	<0.001
Italy	2807.68 (2250.85-3540.01)	15.5 (12.33-19.57)	1915.14 (1534.52-2374.93)	15.35 (12.23-19.24)	-0.53 (-0.61 to -0.45)	<0.001
Jamaica	19.56 (14.05-26.44)	1.73 (1.23-2.35)	17.98 (13.14-24.21)	1.75 (1.26-2.38)	0.55 (0.49 to 0.6)	<0.001
Japan	3056.55 (2304.2-3958.54)	7.12 (5.4-9.27)	5116.67 (4101.92-6339.4)	19.93 (15.81-24.84)	3.23 (3.04 to 3.42)	<0.001

Jordan	94.19 (73.47-119.04)	4.56 (3.52-5.83)	318.38 (244.92-409.85)	6.1 (4.66-7.9)	1.33 (1.18 to 1.49)	<0.001
Kazakhstan	265.02 (197.86-347.9)	4.11 (3.03-5.46)	234.44 (176.82-305.82)	4.27 (3.18-5.62)	-0.22 (-0.42 to -0.02)	0.031
Kenya	74.93 (49.99-106.06)	0.61 (0.4-0.88)	168.55 (114.98-238.68)	0.65 (0.43-0.93)	0.74 (0.7 to 0.78)	<0.001
Kiribati	0.16 (0.1-0.23)	0.48 (0.29-0.72)	0.24 (0.16-0.34)	0.48 (0.3-0.68)	0.25 (0.14 to 0.37)	<0.001
Kuwait	32.36 (27.44-38.02)	5.25 (4.41-6.22)	35.3 (26.06-46.24)	3.34 (2.44-4.44)	-1.11 (-1.82 to -0.39)	0.003
Kyrgyzstan	74.97 (57.05-97.73)	3.87 (2.9-5.1)	91.71 (69.02-120.97)	3.86 (2.92-5.11)	-0.13 (-0.41 to 0.15)	0.376
Lao People's Democratic Republic	9.18 (5.88-13.53)	0.46 (0.28-0.69)	16.93 (11.29-24.05)	0.56 (0.35-0.81)	1.16 (1.1 to 1.21)	<0.001
Latvia	54.14 (42.59-68.12)	6.88 (5.35-8.74)	35.72 (28.21-45.67)	9.07 (7.1-11.74)	0.81 (0.51 to 1.12)	<0.001
Lebanon	30.15 (22.21-41.14)	2.12 (1.53-2.92)	39.66 (28.99-53.4)	2.53 (1.83-3.46)	0.61 (0.34 to 0.87)	<0.001
Lesotho	6.07 (4.07-8.67)	0.67 (0.44-0.96)	7.00 (4.8-9.93)	0.74 (0.49-1.06)	0.79 (0.73 to 0.85)	<0.001
Liberia	6.09 (4.11-8.63)	0.64 (0.42-0.93)	15.37 (10.23-21.88)	0.64 (0.42-0.93)	0.47 (0.3 to 0.64)	<0.001
Libya	35.57 (26.1-48.27)	1.63 (1.18-2.26)	47.9 (35.01-65.11)	1.93 (1.38-2.64)	1.52 (1.36 to 1.67)	<0.001
Lithuania	88 (70.04-110.07)	7.41 (5.84-9.41)	61.87 (51.29-74.97)	10.41 (8.56-12.72)	1.21 (1.07 to 1.35)	<0.001
Luxembourg	9.93 (7.76-12.57)	10.42 (8.1-13.28)	16.1 (12.72-20.6)	11.13 (8.67-14.31)	0.29 (0.07 to 0.5)	0.01
Madagascar	35.45 (24.01-50.04)	0.60 (0.4-0.86)	83.16 (56.21-118.48)	0.63 (0.41-0.9)	0.47 (0.4 to 0.54)	<0.001
Malawi	26.19 (16.94-38.16)	0.57 (0.36-0.83)	61.53 (40.75-87.78)	0.60 (0.38-0.86)	0.82 (0.75 to 0.88)	<0.001
Malaysia	54.52 (38.6-74.18)	0.68 (0.47-0.94)	120 (89.05-157.03)	1.05 (0.77-1.39)	1.97 (1.87 to 2.06)	<0.001
Maldives	0.60 (0.39-0.87)	0.55 (0.34-0.8)	0.89 (0.6-1.25)	0.67 (0.44-0.95)	1.01 (0.82 to 1.21)	<0.001
Mali	22.47 (14.58-32.19)	0.57 (0.36-0.84)	69.43 (46.98-98.78)	0.62 (0.4-0.89)	0.61 (0.57 to 0.65)	<0.001
Malta	8.11 (6.17-10.59)	6.64 (4.95-8.76)	6.43 (5.03-8.19)	7.26 (5.6-9.41)	0.29 (0.2 to 0.38)	<0.001

Marshall Islands	0.12<br>(0.08-0.17)	0.51<br>(0.31-0.74)	0.13<br>(0.09-0.19)	0.54<br>(0.34-0.78)	0.67<br>(0.6 to 0.73)	<0.001
Mauritania	6.82<br>(4.63-9.67)	0.7<br>(0.46-1.01)	16.91<br>(11.67-23.32)	0.83<br>(0.55-1.17)	1.09<br>(1.02 to 1.16)	<0.001
Mauritius	3.04<br>(2.01-4.28)	0.67<br>(0.42-0.96)	3.06<br>(2.12-4.32)	0.81<br>(0.54-1.15)	1.21<br>(1.15 to 1.27)	<0.001
Mexico	755.69<br>(563.54-991.66)	1.72<br>(1.26-2.29)	844.06<br>(635.52-1100.82)	1.79<br>(1.33-2.37)	0.40<br>(0.32 to 0.49)	<0.001
Micronesia (Federated States of)	0.29<br>(0.18-0.41)	0.53<br>(0.33-0.77)	0.26<br>(0.17-0.37)	0.54<br>(0.34-0.79)	0.74<br>(0.7 to 0.78)	<0.001
Monaco	0.38<br>(0.29-0.5)	6.84<br>(5.17-8.98)	0.51<br>(0.39-0.66)	6.61<br>(4.96-8.72)	-0.13<br>(-0.28 to 0.01)	0.074
Mongolia	38.55<br>(28.86-50.77)	3.66<br>(2.71-4.87)	39.01<br>(29.2-50.33)	3.85<br>(2.84-5.07)	-0.24<br>(-0.37 to -0.1)	<0.001
Montenegro	27.51<br>(21.35-35.31)	11.95<br>(9.26-15.39)	20.72<br>(16.11-26.05)	11.97<br>(9.28-15.2)	0.30<br>(0.24 to 0.35)	<0.001
Morocco	169.64<br>(124.19-225.75)	1.41<br>(0.99-1.89)	209.25<br>(154.46-278.1)	1.55<br>(1.12-2.1)	0.71<br>(0.68 to 0.75)	<0.001
Mozambique	34.3<br>(22.64-49.17)	0.53<br>(0.34-0.77)	87.33<br>(56.48-127.24)	0.58<br>(0.37-0.84)	0.38<br>(0.28 to 0.48)	<0.001
Myanmar	90.73<br>(57.94-131.33)	0.48<br>(0.29-0.7)	123.8<br>(80.98-176.39)	0.58<br>(0.37-0.84)	1.06<br>(0.98 to 1.14)	<0.001
Namibia	5.51<br>(3.71-8)	0.76<br>(0.5-1.11)	9.36<br>(6.36-13.11)	0.88<br>(0.57-1.24)	0.59<br>(0.54 to 0.65)	<0.001
Nauru	0.03<br>(0.02-0.04)	0.58<br>(0.38-0.84)	0.03<br>(0.02-0.04)	0.6<br>(0.38-0.86)	0.68<br>(0.4 to 0.95)	<0.001
Nepal	87.34<br>(60.68-124.97)	0.98<br>(0.67-1.41)	154.31<br>(108.07-215.66)	1.1<br>(0.75-1.56)	1.34<br>(1.32 to 1.35)	<0.001
Netherlands	726.81<br>(624.88-847.99)	15.99<br>(13.62-18.93)	199.63<br>(154.09-255.54)	4.58<br>(3.51-5.93)	-4.50<br>(-4.87 to -4.13)	<0.001
New Zealand	127.92<br>(101.67-164.3)	10.05<br>(7.86-12.88)	159.81<br>(128.86-200.28)	12.52<br>(10-15.74)	0.54<br>(0.4 to 0.68)	<0.001
Nicaragua	26.13<br>(18.8-35.24)	1.31<br>(0.92-1.78)	39.79<br>(28.66-52.8)	1.45<br>(1.03-1.94)	0.93<br>(0.88 to 0.97)	<0.001
Niger	21.69<br>(14.13-31.34)	0.57<br>(0.36-0.83)	70.38<br>(45.87-101.62)	0.58<br>(0.38-0.85)	0.24<br>(0.19 to 0.3)	<0.001
Nigeria	262.21<br>(175.45-369.78)	0.61<br>(0.4-0.88)	781.32<br>(535.13-1106.05)	0.71<br>(0.47-1.01)	0.72<br>(0.67 to 0.77)	<0.001
Niue	0.01<br>(0-0.01)	0.62<br>(0.39-0.89)	0<br>(0-0.01)	0.65<br>(0.42-0.91)	0.65<br>(0.54 to 0.77)	<0.001

North Macedonia	85.09 (65.85-108.89)	11.33 (8.69-14.58)	60.51 (47.31-77.03)	11.60 (8.99-14.9)	0.24 (0.14 to 0.34)	<0.001
Northern Mariana Islands	0.12 (0.08-0.17)	0.72 (0.47-1.03)	0.11 (0.08-0.16)	0.71 (0.47-1.02)	1.03 (0.23 to 1.83)	0.012
Norway	178.8 (139.93-231.11)	13.52 (10.53-17.51)	262.95 (208.97-330.32)	18.9 (14.83-23.9)	0.86 (0.75 to 0.96)	<0.001
Oman	17.27 (12.7-22.91)	2.17 (1.57-2.94)	26.88 (19.81-35.77)	2.33 (1.69-3.15)	0.66 (0.48 to 0.83)	<0.001
Pakistan	453.23 (319.02-642.38)	0.83 (0.56-1.19)	1027.16 (719.06-1465.05)	0.93 (0.63-1.34)	0.82 (0.8 to 0.84)	<0.001
Palau	0.04 (0.03-0.06)	0.63 (0.4-0.89)	0.03 (0.02-0.05)	0.65 (0.42-0.92)	0.23 (0.13 to 0.33)	<0.001
Palestine	21.08 (15.63-28.61)	2.05 (1.5-2.78)	54.7 (39.93-73.7)	2.34 (1.7-3.16)	0.97 (0.85 to 1.1)	<0.001
Panama	19.09 (14.06-24.97)	1.72 (1.26-2.28)	25.8 (18.71-34.87)	1.68 (1.2-2.28)	-0.06 (-0.09 to -0.04)	<0.001
Papua New Guinea	8.58 (5.31-12.44)	0.44 (0.26-0.65)	19.14 (12.37-27.57)	0.45 (0.28-0.65)	0.04 (-0.03 to 0.11)	0.229
Paraguay	45.21 (33.16-59.04)	2.45 (1.79-3.28)	57.17 (41.46-75.26)	2.04 (1.45-2.73)	0.00 (-0.13 to 0.13)	0.978
Peru	102.67 (71.29-142.55)	0.98 (0.67-1.38)	121.19 (85.75-168.37)	0.97 (0.67-1.36)	0.18 (0.12 to 0.23)	<0.001
Philippines	159.84 (104.98-226.38)	0.52 (0.33-0.75)	275.2 (184.46-383.31)	0.59 (0.39-0.84)	0.63 (0.55 to 0.71)	<0.001
Poland	1940.84 (1507.1-2475.54)	15.09 (11.66-19.47)	1636.04 (1343.04-1995.41)	20.35 (16.66-25.04)	1.03 (0.95 to 1.12)	<0.001
Portugal	157.51 (122.27-200.7)	4.28 (3.31-5.52)	156.23 (125.48-195.26)	6.43 (5.09-8.12)	1.40 (1.18 to 1.63)	<0.001
Puerto Rico	45.81 (37.49-56.31)	3.14 (2.51-3.89)	25.51 (19.98-32.01)	2.64 (2.04-3.37)	-0.09 (-0.27 to 0.1)	0.374
Qatar	3.20 (2.39-4.35)	2.45 (1.8-3.33)	13.34 (10.02-17.68)	2.76 (2.05-3.71)	0.65 (0.48 to 0.81)	<0.001
Republic of Korea	783.46 (621.96-983.77)	4.08 (3.19-5.17)	1145.25 (1004.73-1299.32)	10.31 (8.91-11.87)	2.80 (2.35 to 3.25)	<0.001
Republic of Moldova	63.57 (47.94-83.32)	4.13 (3.09-5.43)	43.48 (34.68-54.12)	5.08 (4.03-6.38)	1.13 (1.08 to 1.18)	<0.001
Romania	660.35 (510.23-849.47)	7.84 (5.95-10.19)	409.51 (323.26-512.59)	9.09 (7.15-11.51)	0.48 (0.34 to 0.63)	<0.001
Russian Federation	1843.49 (1397.4-2372.72)	3.98 (2.99-5.2)	1321.62 (999.32-1698.48)	3.99 (2.99-5.21)	-0.17 (-0.41 to 0.06)	0.151

Rwanda	21.3 (14.06-30.26)	0.61 (0.39-0.87)	42.57 (28.4-59.96)	0.68 (0.44-0.98)	1.01 (0.9 to 1.12)	<0.001
Saint Kitts and Nevis	0.33 (0.24-0.44)	1.72 (1.25-2.36)	0.33 (0.24-0.44)	1.75 (1.26-2.39)	0.46 (0.42 to 0.5)	<0.001
Saint Lucia	1.07 (0.77-1.44)	1.58 (1.12-2.17)	0.94 (0.68-1.29)	1.68 (1.2-2.34)	0.95 (0.89 to 1)	<0.001
Saint Vincent and the Grenadines	0.85 (0.61-1.13)	1.52 (1.08-2.05)	0.60 (0.44-0.81)	1.54 (1.1-2.1)	0.44 (0.35 to 0.52)	<0.001
Samoa	0.53 (0.34-0.76)	0.59 (0.37-0.84)	0.61 (0.39-0.87)	0.58 (0.37-0.83)	0.02 (-0.04 to 0.08)	0.496
San Marino	0.53 (0.4-0.68)	6.61 (5.04-8.53)	0.56 (0.43-0.72)	6.42 (4.85-8.35)	-0.26 (-0.31 to -0.22)	<0.001
Sao Tome and Principe	0.48 (0.33-0.69)	0.77 (0.51-1.11)	0.87 (0.61-1.21)	0.85 (0.58-1.22)	0.85 (0.73 to 0.97)	<0.001
Saudi Arabia	187.86 (143.87-243.39)	2.48 (1.87-3.28)	303.85 (228.85-397.69)	2.75 (2.05-3.66)	1.21 (1.06 to 1.36)	<0.001
Senegal	24.4 (16.45-34.37)	0.65 (0.43-0.95)	51.91 (34.89-73.54)	0.69 (0.45-0.99)	0.65 (0.59 to 0.7)	<0.001
Serbia	343.45 (261.85-438.65)	11.12 (8.46-14.38)	322.34 (255.4-410.54)	14.2 (11.15-18.2)	0.89 (0.75 to 1.04)	<0.001
Seychelles	0.23 (0.15-0.32)	0.7 (0.45-1.01)	0.24 (0.17-0.34)	0.81 (0.54-1.14)	0.54 (0.47 to 0.6)	<0.001
Sierra Leone	9.92 (6.5-14.15)	0.61 (0.4-0.88)	27.32 (18.43-38.68)	0.66 (0.43-0.96)	0.77 (0.7 to 0.84)	<0.001
Singapore	44.31 (33.15-58.46)	3.93 (2.9-5.19)	41.37 (30.96-55.72)	3.86 (2.87-5.23)	-0.73 (-1.33 to -0.13)	0.018
Slovakia	336.93 (271.56-413.94)	17.73 (14.24-21.97)	212.81 (169.82-264.76)	18.34 (14.63-22.7)	-0.02 (-0.18 to 0.15)	0.833
Slovenia	107.48 (87.57-132.15)	17.16 (13.85-21.31)	78.33 (63.6-95.78)	19.18 (15.4-23.85)	-0.02 (-0.17 to 0.14)	0.836
Solomon Islands	0.79 (0.49-1.15)	0.44 (0.27-0.65)	1.39 (0.88-1.99)	0.45 (0.28-0.66)	0.18 (0.13 to 0.22)	<0.001
Somalia	18.63 (12.13-26.64)	0.49 (0.32-0.72)	49.98 (32.38-71.98)	0.47 (0.3-0.7)	-0.13 (-0.26 to 0.01)	0.073
South Africa	134.27 (91.67-188.98)	0.76 (0.51-1.08)	162.4 (110.95-229.73)	0.81 (0.55-1.15)	0.22 (0.15 to 0.29)	<0.001
South Sudan	18.26 (11.98-25.81)	0.60 (0.39-0.86)	32.11 (21.53-45.53)	0.63 (0.41-0.9)	0.31 (0.26 to 0.36)	<0.001
Spain	2034.29	14.47 (12.65-16.7)	1904.16 (1654.62-2191.64)	19.12 (16.33-22.28)	0.53 (0.36 to 0.7)	<0.001

	(1795.73-2308.76)					
Sri Lanka	59.77 (40.98-82.81)	0.79 (0.53-1.12)	105.19 (79.35-137.03)	1.40 (1.03-1.85)	2.20 (2 to 2.4)	<0.001
Sudan	170.77 (126.55-233.86)	1.75 (1.27-2.41)	410.12 (303.01-547.84)	2.06 (1.49-2.8)	0.99 (0.95 to 1.03)	<0.001
Suriname	2.65 (1.9-3.59)	1.52 (1.07-2.09)	3.17 (2.29-4.25)	1.53 (1.09-2.08)	0.21 (0.13 to 0.29)	<0.001
Sweden	341.24 (265.22-430.17)	14.36 (11.02-18.38)	319.58 (249.2-401.55)	12.9 (10.03-16.25)	-0.67 (-0.83 to -0.52)	<0.001
Switzerland	194.45 (154.31-246.55)	10.94 (8.57-13.96)	230.35 (183.88-290.56)	12.4 (9.79-15.68)	0.26 (0.2 to 0.32)	<0.001
Syrian Arab Republic	130.46 (96.29-175.03)	1.96 (1.43-2.67)	171.09 (125.91-231.59)	2.24 (1.62-3.07)	1.81 (1.77 to 1.85)	<0.001
Taiwan (Province of China)	96.57 (71.46-126.89)	1.21 (0.87-1.62)	126.67 (98.07-160.44)	2.50 (1.92-3.19)	2.79 (2.48 to 3.10)	<0.001
Tajikistan	88.61 (66.93-115.92)	3.56 (2.66-4.74)	141.03 (106.59-187.2)	3.65 (2.73-4.9)	0.33 (0.22 to 0.44)	<0.001
Thailand	121.71 (77.22-178.93)	0.48 (0.29-0.73)	116.27 (78.11-161.91)	0.65 (0.43-0.94)	1.37 (1.18 to 1.57)	<0.001
Timor-Leste	1.57 (0.99-2.29)	0.46 (0.28-0.68)	3.96 (2.59-5.69)	0.57 (0.37-0.83)	1.56 (1.49 to 1.63)	<0.001
Togo	13.28 (9.04-18.46)	0.72 (0.47-1.03)	26.32 (17.66-37.66)	0.71 (0.46-1.03)	0.28 (0.26 to 0.29)	<0.001
Tokelau	0.00 (0-0.01)	0.55 (0.35-0.8)	0 (0-0)	0.65 (0.42-0.92)	0.85 (0.37 to 1.33)	<0.001
Tonga	0.30 (0.19-0.43)	0.58 (0.37-0.84)	0.28 (0.19-0.4)	0.60 (0.39-0.86)	0.08 (0.05 to 0.11)	<0.001
Trinidad and Tobago	8.67 (6.29-11.77)	1.70 (1.21-2.33)	6.75 (4.88-9.17)	1.72 (1.22-2.37)	0.42 (0.26 to 0.59)	<0.001
Tunisia	89.02 (65.55-119.96)	2.25 (1.63-3.08)	89.94 (65.9-120.6)	2.53 (1.84-3.41)	0.63 (0.55 to 0.72)	<0.001
Turkey	1114.25 (863.57-1422.54)	3.93 (3.01-5.03)	1687.34 (1314-2150.68)	6.17 (4.75-7.94)	2.25 (2 to 2.49)	<0.001
Turkmenistan	64.26 (48.42-83.24)	3.74 (2.79-4.93)	73.00 (55.18-95.32)	4.08 (3.08-5.38)	0.38 (0.26 to 0.5)	<0.001
Tuvalu	0.02 (0.01-0.03)	0.52 (0.33-0.77)	0.03 (0.02-0.04)	0.54 (0.35-0.78)	0.81 (0.76 to 0.86)	<0.001
Uganda	48.77 (31.99-69.85)	0.56 (0.36-0.81)	139.24 (93.45-196.95)	0.63 (0.41-0.91)	0.76 (0.68 to 0.85)	<0.001

Ukraine	489.72 (367.81-643.18)	3.06 (2.27-4.07)	284.8 (215.1-372.37)	3.1 (2.3-4.1)	-0.08 (-0.28 to 0.13)	0.461
United Arab Emirates	13.78 (10.34-18.22)	2.47 (1.82-3.34)	44.42 (33.32-58.92)	2.86 (2.13-3.84)	1.48 (1.11 to 1.86)	<0.001
United Kingdom	2338.05 (1907-2892.27)	14 (11.32-17.42)	2369.19 (1942.85-2942.76)	14.56 (11.72-18.16)	-0.06 (-0.2 to 0.08)	0.421
United Republic of Tanzania	85.12 (57.32-120.63)	0.65 (0.43-0.93)	192.35 (128.1-272.47)	0.69 (0.45-0.99)	0.34 (0.31 to 0.38)	<0.001
United States of America	15612.41 (12329.2-19593.36)	19.92 (15.75-25.13)	20883.9 (17621.78-24674.02)	22.34 (18.58-26.77)	0.26 (0.22 to 0.3)	<0.001
United States Virgin Islands	0.8 (0.59-1.07)	1.85 (1.34-2.49)	0.55 (0.4-0.72)	1.93 (1.39-2.59)	0.69 (0.59 to 0.78)	<0.001
Uruguay	16.05 (11.61-21.73)	1.42 (1-1.94)	15.24 (11.17-20.5)	1.44 (1.03-1.94)	0.23 (0.18 to 0.28)	<0.001
Uzbekistan	356.21 (266.84-464.29)	3.74 (2.78-4.93)	500.74 (376.42-656.12)	3.94 (2.94-5.24)	0.4 (0.22 to 0.57)	<0.001
Vanuatu	0.35 (0.22-0.51)	0.49 (0.3-0.72)	0.67 (0.43-0.97)	0.50 (0.31-0.74)	0.37 (0.33 to 0.4)	<0.001
Venezuela (Bolivarian Republic of)	151.5 (110.93-200.27)	1.71 (1.24-2.27)	157.57 (114.44-209.75)	1.59 (1.15-2.15)	0.01 (-0.1 to 0.13)	0.832
Viet Nam	328.27 (218.48-465.24)	1.02 (0.66-1.45)	467.72 (325.16-652.96)	1.58 (1.08-2.24)	1.75 (1.47 to 2.04)	<0.001
Yemen	108.67 (79.48-147.03)	1.66 (1.19-2.3)	294.43 (215.1-398.07)	1.91 (1.37-2.62)	1.18 (1.13 to 1.23)	<0.001
Zambia	26.46 (17.79-37.41)	0.62 (0.41-0.89)	68.00 (46.01-94.23)	0.73 (0.49-1.03)	0.74 (0.67 to 0.81)	<0.001
Zimbabwe	40.80 (27.64-58.16)	0.73 (0.48-1.05)	54.57 (36.77-78.03)	0.74 (0.49-1.05)	0.21 (0.11 to 0.32)	<0.001

Supplementary table 2. The relative change in prevalence, incidence, DALYs, and mortality cases of IBD between 1990 and 2019.

location	prevalence number changes (%)	incidence number changes (%)	DALYs number changes (%)	mortality number changes (%)
Afghanistan	240.25	245.79	131.12	123.89
Albania	-42.86	-43.32	-92.26	-92.62
Algeria	7.42	10.94	-7.24	-12.77

American Samoa	24.07	31.46	-66.91	-66.87
Andorra	4.24	16.42	-40.18	-52.08
Angola	249.62	248.02	35.37	36.70
Antigua and Barbuda	15.82	21.63	-28.04	-29.94
Argentina	22.66	19.75	-17.12	-20.25
Armenia	-36.56	-34.08	-42.65	-46.68
Australia	90.22	113.02	20.29	-15.96
Austria	-23.57	-11.47	-60.37	-72.43
Azerbaijan	1.95	1.87	-27.36	-40.24
Bahamas	6.52	27.33	-43.41	-44.15
Bahrain	109.41	101.91	51.07	37.79
Bangladesh	46.91	39.60	-65.60	-64.90
Barbados	-16.41	-11.15	-55.46	-59.88
Belarus	-33.73	-29.19	-49.39	-55.01
Belgium	-16.14	-4.61	-33.10	-42.10
Belize	117.00	119.73	-50.44	-49.80
Benin	208.09	189.62	40.87	42.30
Bermuda	-19.65	-13.74	-73.27	-75.04
Bhutan	13.24	1.15	-59.96	-60.73
Bolivia (Plurinational State of)	56.91	54.99	-66.61	-66.50
Bosnia and Herzegovina	-44.44	-43.67	-56.18	-63.86
Botswana	61.07	49.01	74.35	73.09
Brazil	-9.46	-12.87	-35.25	-36.65
Brunei Darussalam	52.41	44.82	-27.92	-37.56
Bulgaria	-46.60	-42.49	-65.79	-68.86
Burkina Faso	162.85	147.53	74.06	75.16
Burundi	118.66	124.83	-13.06	-11.61
Cabo Verde	46.40	41.78	-43.27	-44.60
Cambodia	63.45	57.88	-59.22	-59.09
Cameroon	227.74	208.71	71.47	72.97
Canada	-6.61	5.49	-19.69	-51.89
Central African Republic	102.25	97.56	36.23	36.97
Chad	220.81	211.66	96.73	98.34
Chile	6.63	4.65	-50.50	-60.36
China	22.60	27.73	-86.72	-87.93
Colombia	14.16	9.26	-32.31	-33.21
Comoros	40.87	40.72	-31.53	-30.64
Congo	102.74	116.35	-14.83	-14.45
Cook Islands	-27.41	-18.62	-82.67	-82.36
Costa Rica	18.50	7.03	-12.47	-20.55
C 么 te d'Ivoire	122.18	124.31	30.42	31.40
Croatia	-34.59	-27.20	-41.76	-53.32

Cuba	-38.50	-31.86	-66.60	-75.70
Cyprus	60.31	91.66	-51.29	-61.00
Czechia	8.09	26.99	-37.91	-62.44
Democratic People's Republic of Korea	-3.51	6.97	-79.71	-80.16
Democratic Republic of the Congo	132.50	153.86	-13.37	-12.00
Denmark	0.00	5.18	-12.94	-42.92
Djibouti	121.29	104.38	38.00	38.11
Dominica	-30.72	-19.64	-53.08	-53.62
Dominican Republic	20.75	32.89	-43.96	-45.03
Ecuador	90.52	88.46	-56.87	-57.75
Egypt	52.97	46.87	7.05	-6.63
El Salvador	-2.06	-9.52	-91.15	-92.81
Equatorial Guinea	453.92	449.37	-12.85	-12.32
Eritrea	141.26	142.69	23.44	24.95
Estonia	-34.85	-26.51	-81.69	-85.51
Eswatini	39.05	36.73	-19.62	-18.56
Ethiopia	146.05	140.35	-33.69	-33.22
Fiji	4.34	15.22	7.01	6.23
Finland	-27.73	-17.61	-34.56	-53.17
France	0.93	14.80	-13.75	-25.50
Gabon	92.85	93.78	-31.31	-31.35
Gambia	154.41	178.98	-17.18	-15.92
Georgia	-52.60	-52.06	-68.03	-75.98
Germany	30.42	34.68	-10.10	-21.55
Ghana	125.05	121.57	-1.19	0.03
Greece	-31.12	-17.38	-47.81	-64.89
Greenland	7.95	14.55	-43.72	-63.88
Grenada	-4.89	0.24	-57.73	-56.79
Guam	16.11	32.97	-39.61	-42.02
Guatemala	132.63	113.77	-18.49	-16.70
Guinea	145.43	137.20	0.41	1.33
Guinea-Bissau	104.92	100.20	-34.58	-33.42
Guyana	-17.45	-12.51	-51.60	-50.81
Haiti	89.80	96.04	-33.71	-33.40
Honduras	100.77	80.98	-50.78	-50.83
Hungary	-12.33	-4.65	-45.52	-67.65
Iceland	35.71	61.97	-15.68	-44.57
India	38.13	32.95	-54.18	-54.86
Indonesia	31.90	32.25	-65.27	-65.00
Iran (Islamic Republic of)	-11.36	-15.59	-21.86	-21.32
Iraq	161.80	152.24	-29.70	-33.11

Ireland	-2.45	-3.10	-34.28	-55.77
Israel	44.43	66.07	-6.37	-20.56
Italy	-31.79	-27.07	-61.65	-72.60
Jamaica	-8.08	-1.05	-77.65	-80.32
Japan	67.40	98.54	5.59	-76.24
Jordan	238.01	233.43	92.20	43.09
Kazakhstan	-11.54	-5.95	-37.68	-39.36
Kenya	124.94	131.99	-16.98	-14.73
Kiribati	55.17	69.24	-44.35	-43.48
Kuwait	9.11	10.15	-2.88	-5.76
Kyrgyzstan	22.32	25.15	-70.56	-73.96
Lao People's Democratic Republic	84.46	83.07	-43.73	-43.77
Latvia	-34.02	-29.86	-65.60	-74.97
Lebanon	31.54	49.26	22.56	20.07
Lesotho	15.32	12.21	1.23	1.88
Liberia	152.22	158.58	-40.26	-38.76
Libya	34.69	44.78	-15.93	-26.62
Lithuania	-29.69	-21.54	-48.29	-63.94
Luxembourg	62.19	58.99	-5.52	-27.54
Madagascar	134.61	129.23	-25.25	-24.29
Malawi	134.92	133.82	-45.96	-45.08
Malaysia	120.12	122.33	-4.36	-16.85
Maldives	48.97	55.50	-54.71	-55.66
Mali	209.02	201.08	38.87	40.08
Malta	-20.71	-15.27	-32.43	-35.34
Marshall Islands	8.50	18.02	-36.54	-35.91
Mauritania	147.97	113.89	-40.96	-41.27
Mauritius	0.66	-2.93	57.25	73.23
Mexico	11.69	2.72	-35.11	-35.64
Micronesia (Federated States of)	-10.12	-0.24	-68.73	-68.18
Monaco	34.11	44.00	-18.45	-40.53
Mongolia	1.20	7.62	-79.95	-81.46
Montenegro	-24.69	-25.09	-60.89	-71.34
Morocco	23.35	20.97	-9.73	-14.98
Mozambique	154.59	160.40	-1.23	0.65
Myanmar	36.44	39.37	-75.12	-75.90
Namibia	69.88	58.65	7.46	7.66
Nauru	11.79	22.02	-52.09	-51.70
Nepal	76.68	80.61	-64.28	-64.73
Netherlands	-72.53	-71.75	-59.99	-55.98
New Zealand	24.93	37.05	-9.48	-69.90
Nicaragua	52.30	28.34	-9.35	-13.96

Niger	224.47	217.35	35.40	36.58
Nigeria	197.97	183.28	49.68	50.51
Niue	-37.42	-30.08	-63.78	-63.63
North Macedonia	-28.89	-29.30	-52.93	-69.79
Northern Mariana Islands	-5.68	2.79	-80.42	-81.08
Norway	47.06	77.19	-2.15	-60.57
Oman	55.58	52.59	32.93	26.96
Pakistan	126.63	119.33	38.42	40.80
Palau	-23.17	-11.01	-64.75	-64.55
Palestine	159.49	181.09	8.56	-21.20
Panama	35.13	20.70	48.33	50.17
Papua New Guinea	123.14	147.02	74.36	74.50
Paraguay	26.46	20.99	-52.26	-60.11
Peru	18.04	19.03	-91.90	-91.86
Philippines	72.18	80.84	-14.15	-16.03
Poland	-15.70	-24.84	-51.72	-71.16
Portugal	-0.82	23.08	-48.79	-62.25
Puerto Rico	-44.31	-41.96	-79.14	-82.66
Qatar	317.25	329.29	131.53	111.75
Republic of Korea	46.18	58.02	-78.00	-94.57
Republic of Moldova	-31.60	-26.75	-67.78	-72.85
Romania	-37.99	-36.22	-80.69	-84.53
Russian Federation	-28.31	-24.68	-54.71	-60.47
Rwanda	99.90	84.40	-34.78	-34.35
Saint Kitts and Nevis	1.28	9.81	-62.15	-61.60
Saint Lucia	-12.28	-13.81	-72.05	-72.34
Saint Vincent and the Grenadines	-29.22	-24.57	-69.32	-68.66
Samoa	14.01	31.50	-57.57	-57.28
San Marino	6.28	18.07	-17.68	-31.24
Sao Tome and Principe	79.23	80.40	-59.43	-58.82
Saudi Arabia	61.75	47.45	-1.01	-24.14
Senegal	112.71	111.13	-38.44	-36.93
Serbia	-6.15	-1.05	-59.65	-74.12
Seychelles	6.76	8.25	-25.33	-27.64
Sierra Leone	175.38	160.90	7.57	9.16
Singapore	-6.63	-5.98	-63.90	-80.40
Slovakia	-36.84	-38.33	-42.77	-44.49
Slovenia	-27.12	-18.94	-46.02	-70.59
Solomon Islands	75.63	95.27	19.57	19.58
Somalia	168.31	165.87	55.57	56.93
South Africa	20.95	18.14	-57.48	-57.96
South Sudan	75.80	71.49	-14.15	-13.62

Spain	-6.40	-1.86	-34.47	-70.93
Sri Lanka	75.98	82.41	-63.75	-70.72
Sudan	140.16	138.81	24.03	15.92
Suriname	19.53	27.74	-58.60	-58.59
Sweden	-6.35	5.29	-39.30	-59.23
Switzerland	18.46	22.27	-34.76	-62.17
Syrian Arab Republic	31.15	22.89	-38.45	-45.86
Taiwan (Province of China)	31.17	42.68	-77.42	-82.29
Tajikistan	59.17	59.79	-74.77	-76.11
Thailand	-4.47	-3.67	-73.57	-76.54
Timor-Leste	152.14	151.64	-29.56	-29.37
Togo	98.21	121.99	-15.93	-14.47
Tokelau	-10.78	-13.12	-73.23	-73.20
Tonga	-6.82	11.10	-44.70	-44.33
Trinidad and Tobago	-22.16	-17.53	-49.60	-52.32
Tunisia	1.04	0.40	-27.58	-36.72
Turkey	51.43	37.48	-60.22	-65.70
Turkmenistan	13.60	13.59	-61.07	-63.18
Tuvalu	41.53	61.05	-75.76	-75.12
Uganda	185.50	186.70	50.53	52.55
Ukraine	-41.84	-38.57	-40.12	-38.35
United Arab Emirates	222.35	215.15	102.29	34.21
United Kingdom	1.33	17.34	-23.52	-29.93
United Republic of Tanzania	125.98	129.12	39.20	39.80
United States of America	33.76	31.32	9.95	-2.47
United States Virgin Islands	-31.32	-26.29	-76.88	-79.20
Uruguay	-5.07	7.38	-2.26	0.47
Uzbekistan	40.57	48.40	-34.37	-35.78
Vanuatu	89.30	111.53	22.86	23.87
Venezuela (Bolivarian Republic of)	4.01	-3.03	-61.81	-66.67
Viet Nam	42.48	52.65	-59.36	-63.62
Yemen	170.94	161.72	85.69	77.34
Zambia	157.03	130.93	-27.32	-26.54
Zimbabwe	33.75	34.28	40.48	41.72

Supplementary table 3. Cases and age-standardized for IBD incidence and their average annual percentage changes (AAPCs) from 1990 to 2019 at the global and regional levels.

	1990		2019		AAPCs, 1990-2019 (95% CI)	P value
	cases (n)	ASIR (Per 100,000 population,	cases (n)	ASIR (Per 100,000 population,		

		95% CI)		95% CI)		
Global	20897.42 (17008.63-25520.15)	0.92 (0.74-1.15)	25658.55 (21268.45-31075.58)	0.95 (0.77-1.17)	0.26 (0.22 to 0.31)	<0.001
Sex						
Male	11087.65 (9019.73-13552.78)	0.96 (0.77-1.19)	13776.05 (11347.31-16776.89)	1.00 (0.8-1.23)	0.29 (0.25 to 0.34)	<0.001
Female	9809.77 (8020.99-12010.23)	0.88 (0.71-1.1)	11882.5 (9902.72-14308.03)	0.91 (0.74-1.11)	0.23 (0.18 to 0.28)	<0.001
Age group, years						
<5	143.36 (93.1-204.65)	0.02 (0.01-0.03)	157.3 (103.02-225.07)	0.02 (0.02-0.03)	0.15 (0.06 to 0.23)	0.001
5-9	1690.61 (1306.15-2157.09)	0.29 (0.22-0.37)	1896.22 (1450.02-2455.82)	0.29 (0.22-0.38)	0.03 (-0.02 to 0.08)	0.212
10-14	5578.13 (4491.67-7011.52)	1.04 (0.84-1.31)	6887.51 (5570.88-8606.81)	1.07 (0.87-1.34)	0.08 (0.01 to 0.15)	0.017
15-19	13485.33 (10855.69-16614.69)	2.60 (2.09-3.2)	16717.52 (13619.95-20285.89)	2.70 (2.2-3.27)	0.10 (0.02 to 0.19)	0.013
Sociodemographic index						
High	10935.98 (9197.71-12981.57)	4.25 (3.52-5.13)	13913.9 (11963.49-16167.92)	5.68 (4.77-6.77)	1.02 (0.97 to 1.08)	<0.001
High-middle	4885.82 (3932.28-6017.12)	1.13 (0.9-1.42)	4710.52 (3868.76-5723.47)	1.34 (1.07-1.66)	0.58 (0.5 to 0.66)	<0.001
Middle	2788.36 (2094.89-3662.48)	0.35 (0.26-0.48)	3385.45 (2549.53-4401.05)	0.43 (0.32-0.58)	0.77 (0.62 to 0.92)	<0.001
Low-middle	1690.07 (1243.63-2287.41)	0.32 (0.22-0.44)	2327.06 (1707.23-3149.61)	0.32 (0.22-0.44)	0.38 (0.23 to 0.53)	<0.001
Low	589.16 (422.05-809.91)	0.23 (0.16-0.32)	1311.56 (927.82-1800.27)	0.23 (0.16-0.32)	0.33 (0.25 to 0.41)	<0.001
Region						

Andean Latin America	49.54 (36.02-66.41)	0.27 (0.19-0.37)	71.17 (53.63-93.91)	0.30 (0.21-0.40)	0.52 (0.46 to 0.58)	<0.001
Australasia	184.56 (145.37-230.65)	2.64 (2.04-3.35)	363.18 (297.58-443.28)	4.81 (3.86-6.00)	1.87 (1.81 to 1.94)	<0.001
Caribbean	68.33 (52.49-88.69)	0.44 (0.32-0.59)	73.22 (54.77-96.5)	0.45 (0.32-0.61)	0.16 (0.11 to 0.21)	<0.001
Central Asia	277.12 (212.33-358.71)	0.94 (0.7-1.23)	323.12 (244.22-416.48)	1.00 (0.75-1.32)	0.28 (0.19 to 0.37)	<0.001
Central Europe	1289.42 (1034.02-1599.08)	3.07 (2.42-3.86)	999.53 (829.12-1195.86)	3.99 (3.24-4.89)	0.78 (0.65 to 0.91)	<0.001
Central Latin America	371.38 (276.06-481.65)	0.46 (0.33-0.61)	408.93 (307.58-531.68)	0.44 (0.32-0.59)	0.17 (0.07 to 0.27)	0.001
Central Sub-Saharan Africa	49.62 (34.05-68.71)	0.18 (0.12-0.26)	132.81 (91.84-187.36)	0.20 (0.13-0.29)	0.60 (0.57 to 0.63)	<0.001
East Asia	1720.33 (1255.8-2301.41)	0.33 (0.24-0.46)	2195.43 (1676.36-2810.77)	0.67 (0.50-0.88)	2.27 (2.08 to 2.45)	<0.001
Eastern Europe	637.17 (487.39-814.01)	0.92 (0.69-1.2)	462.14 (358.49-589.48)	0.99 (0.75-1.28)	0.05 (-0.07 to 0.18)	0.397
Eastern Sub-Saharan Africa	157.46 (109-218.79)	0.16 (0.11-0.23)	373.58 (258.75-517.68)	0.17 (0.11-0.25)	0.55 (0.52 to 0.57)	<0.001
High-income Asia Pacific	1024.53 (797.62-1303.35)	1.66 (1.27-2.15)	1935.62 (1580.81-2354.71)	5.15 (4.14-6.38)	3.71 (3.44 to 3.98)	<0.001
High-income North America	6134.08 (5150.83-7298.53)	7.18 (5.93-8.68)	7659.4 (6679.4-8784.7)	7.62 (6.47-8.93)	0.46 (0.39 to 0.53)	<0.001
North Africa and Middle East	1125.36 (878.88-1423.54)	0.67 (0.51-0.87)	1716.45 (1318.55-2235.9)	0.74 (0.55-0.99)	0.68 (0.52 to 0.84)	<0.001
Oceania	3.79 (2.52-5.27)	0.12 (0.08-0.17)	7.79 (5.24-10.86)	0.13 (0.09-0.19)	0.37 (0.33 to 0.41)	<0.001
South Asia	1521.15 (1073.06-2130.22)	0.3 (0.21-0.43)	2164.46 (1532.65-3011.97)	0.29 (0.20-0.41)	0.43 (0.25 to 0.61)	<0.001

Southeast Asia	322.12 (221.21-440.13)	0.15 (0.1-0.21)	476.07 (341.53-635.71)	0.20 (0.14-0.27)	1.30 (1.23 to 1.37)	<0.001
Southern Latin America	91.37 (69.22-118.94)	0.47 (0.34-0.62)	103.27 (77.65-135.06)	0.48 (0.35-0.65)	0.33 (0.24 to 0.42)	<0.001
Southern Sub-Saharan Africa	50.92 (35.73-70.11)	0.2 (0.13-0.28)	63.15 (44.49-86.7)	0.20 (0.14-0.29)	0.21 (0.18 to 0.25)	<0.001
Tropical Latin America	733.37 (582.7-916.87)	1.04 (0.8-1.33)	643.33 (506.4-812.32)	0.89 (0.67-1.15)	-0.29 (-0.34 to -0.24)	<0.001
Western Europe	4926.08 (4271.97-5729.68)	4.37 (3.71-5.15)	5057.24 (4335.00-5946.42)	5.01 (4.19-5.97)	0.29 (0.23 to 0.35)	<0.001
Western Sub-Saharan Africa	159.71 (110.47-223.73)	0.17 (0.11-0.24)	428.66 (299.24-591.37)	0.18 (0.12-0.26)	0.51 (0.47 to 0.55)	<0.001

Supplementary table 4. Cases and age-standardized for IBD incidence and their average annual percentage changes (AAPCs) from 1990 to 2019 at the national levels.

	1990		2019		AAPCs, 1990-2019 (95% CI)	P value
	cases (n)	ASIR (per 100,000 population, 95% CI)	cases (n)	ASIR (per 100,000 population, 95% CI)		
Afghanistan	29.3 (21.38-40.11)	0.48 (0.34-0.68)	101.33 (74.73-139.34)	0.51 (0.36-0.71)	0.04 (-0.14 to 0.23)	0.632
Albania	35.07 (27.21-44.9)	2.4 (1.83-3.13)	19.88 (15.16-25.37)	2.59 (1.95-3.35)	0.79 (0.67 to 0.9)	<0.001
Algeria	91.81 (70.92-116.9)	0.71 (0.53-0.93)	101.85 (76.4-136)	0.73 (0.53-1.01)	0.01 (-0.2 to 0.23)	0.896
American Samoa	0.03 (0.02-0.04)	0.14 (0.09-0.2)	0.04 (0.03-0.06)	0.16 (0.11-0.23)	1.13 (1.09 to 1.17)	<0.001
Andorra	0.32 (0.24-0.41)	1.94 (1.47-2.55)	0.37 (0.28-0.48)	2.06 (1.56-2.69)	0.20 (0.11 to 0.28)	<0.001
Angola	9.20 (6.32-12.87)	0.18 (0.12-0.26)	32.00 (22.19-44.35)	0.21 (0.14-0.3)	0.57 (0.53 to 0.61)	<0.001
Antigua and Barbuda	0.11 (0.08-0.14)	0.43 (0.31-0.58)	0.13 (0.09-0.17)	0.46 (0.33-0.63)	0.78 (0.73 to 0.82)	<0.001
Argentina	49.96 (36.34-66.32)	0.38 (0.27-0.52)	59.83 (43.62-79.21)	0.40 (0.28-0.54)	0.33 (0.27 to 0.39)	<0.001

Armenia	11.46<br>(8.71-14.71)	0.90<br>(0.66-1.2)	7.56<br>(5.78-9.75)	1.00<br>(0.75-1.32)	0.50<br>(0.2 to 0.8)	0.001
Australia	145.12<br>(112.91-181.77)	2.53<br>(1.93-3.21)	309.14<br>(252.03-378.58)	4.93<br>(3.93-6.17)	2.08<br>(2.01 to 2.14)	<0.001
Austria	159.46<br>(135.5-186.1)	7.47<br>(6.19-8.91)	141.17<br>(120.68-165.77)	7.36<br>(6.08-8.9)	-0.07<br>(-0.38 to 0.24)	0.645
Azerbaijan	30.36<br>(23.24-39.48)	0.99<br>(0.74-1.32)	30.93<br>(23.65-39.9)	1.03<br>(0.77-1.36)	0.25<br>(0.06 to 0.43)	0.008
Bahamas	0.53<br>(0.39-0.7)	0.46<br>(0.33-0.63)	0.68<br>(0.49-0.92)	0.52<br>(0.37-0.73)	0.70<br>(0.62 to 0.79)	<0.001
Bahrain	1.20<br>(0.92-1.52)	0.68<br>(0.51-0.89)	2.42<br>(1.85-3.22)	0.67<br>(0.49-0.92)	0.75<br>(0.49 to 1.01)	<0.001
Bangladesh	153.17<br>(109.73-213.8)	0.28<br>(0.19-0.41)	213.83<br>(152.24-301.89)	0.33<br>(0.23-0.47)	1.19<br>(1.15 to 1.22)	<0.001
Barbados	0.77<br>(0.58-1)	0.81<br>(0.59-1.09)	0.69<br>(0.52-0.9)	0.83<br>(0.61-1.12)	0.32<br>(0.26 to 0.38)	<0.001
Belarus	35.00<br>(26.97-44.97)	1.09<br>(0.82-1.42)	24.79<br>(19.08-31.81)	1.23<br>(0.92-1.61)	0.24<br>(0.07 to 0.42)	0.006
Belgium	94.16<br>(82.6-107.34)	3.43<br>(2.93-4.02)	89.82<br>(70.18-114.83)	3.29<br>(2.51-4.25)	-0.30<br>(-0.44 to -0.16)	<0.001
Belize	0.38<br>(0.28-0.51)	0.39<br>(0.28-0.55)	0.83<br>(0.61-1.11)	0.44<br>(0.32-0.61)	1.02<br>(0.96 to 1.08)	<0.001
Benin	4.23<br>(2.95-5.94)	0.18<br>(0.12-0.26)	12.27<br>(8.57-16.99)	0.19<br>(0.13-0.28)	0.55<br>(0.53 to 0.56)	<0.001
Bermuda	0.08<br>(0.06-0.1)	0.45<br>(0.33-0.62)	0.07<br>(0.05-0.09)	0.49<br>(0.36-0.67)	0.50<br>(0.47 to 0.53)	<0.001
Bhutan	0.88<br>(0.61-1.25)	0.27<br>(0.19-0.4)	0.89<br>(0.62-1.26)	0.30<br>(0.2-0.44)	0.76<br>(0.72 to 0.8)	<0.001
Bolivia (Plurinational State of)	7.91<br>(5.66-10.89)	0.26<br>(0.17-0.36)	12.27<br>(8.83-16.51)	0.26<br>(0.18-0.36)	0.16<br>(0.11 to 0.21)	<0.001
Bosnia and Herzegovina	36.56<br>(29.3-45.94)	2.22<br>(1.74-2.86)	20.59<br>(16.65-25.28)	2.69<br>(2.12-3.36)	0.81<br>(0.68 to 0.93)	<0.001
Botswana	1.48<br>(1.02-2.07)	0.21<br>(0.14-0.3)	2.21<br>(1.54-3.06)	0.24<br>(0.16-0.33)	0.56<br>(0.49 to 0.63)	<0.001
Brazil	720.55<br>(573.05-900.53)	1.05<br>(0.81-1.34)	627.82<br>(494.73-792.96)	0.90<br>(0.69-1.16)	-0.27<br>(-0.32 to -0.22)	<0.001
Brunei Darussalam	1.45<br>(1.08-1.91)	1.34<br>(0.98-1.82)	2.10<br>(1.6-2.8)	1.41<br>(1.04-1.91)	0.84<br>(0.8 to 0.87)	<0.001
Bulgaria	65.46<br>(50.5-83.75)	2.46<br>(1.87-3.21)	37.64<br>(28.93-48.18)	2.71<br>(2.05-3.52)	0.08<br>(0 to 0.16)	0.06
Burkina Faso	8.11<br>(5.39-11.48)	0.17<br>(0.11-0.24)	20.08<br>(13.54-28.18)	0.18<br>(0.11-0.25)	0.32<br>(0.3 to 0.34)	<0.001
Burundi	4.54<br>(3.08-6.34)	0.17<br>(0.11-0.25)	10.22<br>(6.97-14.32)	0.17<br>(0.11-0.25)	0.24<br>(0.18 to 0.3)	<0.001

Cabo Verde	0.32 (0.22-0.44)	0.18 (0.12-0.25)	0.45 (0.32-0.61)	0.21 (0.14-0.3)	0.92 (0.84 to 1)	<0.001
Cambodia	6.53 (4.37-9.1)	0.13 (0.08-0.19)	10.32 (7.03-14.38)	0.16 (0.1-0.23)	1.17 (1.12 to 1.22)	<0.001
Cameroon	10.13 (7-14.14)	0.20 (0.13-0.28)	31.28 (21.66-43.42)	0.21 (0.14-0.3)	0.58 (0.48 to 0.69)	<0.001
Canada	1532.41 (1441.65-1621.79)	18.98 (17.54-20.52)	1616.49 (1505.75-1719.01)	18.56 (16.91-20.21)	0.08 (-0.02 to 0.18)	0.133
Central African Republic	2.40 (1.64-3.36)	0.18 (0.12-0.26)	4.75 (3.22-6.61)	0.18 (0.12-0.26)	0.21 (0.14 to 0.28)	<0.001
Chad	4.78 (3.18-6.8)	0.16 (0.1-0.24)	14.91 (10.11-21.29)	0.17 (0.11-0.25)	0.31 (0.26 to 0.36)	<0.001
Chile	37.44 (29.07-48.54)	0.69 (0.52-0.91)	39.18 (30.37-50.87)	0.75 (0.56-0.99)	0.54 (0.43 to 0.65)	<0.001
China	1675.81 (1223.36-2243.34)	0.34 (0.24-0.46)	2140.47 (1633.94-2740.23)	0.68 (0.51-0.89)	2.28 (2.09 to 2.47)	<0.001
Colombia	61.21 (45.07-81.37)	0.41 (0.29-0.56)	66.88 (49.33-89.78)	0.41 (0.3-0.56)	0.37 (0.25 to 0.49)	<0.001
Comoros	0.43 (0.3-0.6)	0.18 (0.12-0.25)	0.61 (0.42-0.84)	0.19 (0.13-0.27)	0.69 (0.65 to 0.74)	<0.001
Congo	2.51 (1.75-3.44)	0.20 (0.13-0.28)	5.44 (3.84-7.52)	0.23 (0.15-0.32)	0.57 (0.53 to 0.62)	<0.001
Cook Islands	0.01 (0.01-0.02)	0.14 (0.09-0.21)	0.01 (0.01-0.01)	0.18 (0.12-0.25)	0.87 (0.8 to 0.94)	<0.001
Costa Rica	5.87 (4.43-7.74)	0.44 (0.32-0.59)	6.28 (4.68-8.25)	0.41 (0.3-0.55)	0.24 (0.18 to 0.31)	<0.001
Croatia	71.64 (62.81-80.65)	4.92 (4.21-5.7)	52.15 (44.44-60.62)	5.72 (4.74-6.8)	0.54 (0.42 to 0.67)	<0.001
Cuba	18.12 (13.46-24.37)	0.42 (0.3-0.59)	12.35 (9.05-16.46)	0.44 (0.32-0.61)	0.05 (-0.08 to 0.18)	0.42
Cyprus	2.81 (2.17-3.59)	1.05 (0.78-1.37)	5.38 (4.43-6.53)	1.89 (1.51-2.35)	1.96 (1.84 to 2.09)	<0.001
Czechia	60.85 (48.62-76.68)	1.72 (1.34-2.21)	77.27 (64.13-92.88)	3.56 (2.89-4.38)	1.98 (1.74 to 2.22)	<0.001
C 么 te d'Ivoire	11.14 (7.73-15.5)	0.19 (0.12-0.27)	25.00 (17.44-34.83)	0.20 (0.13-0.29)	0.52 (0.45 to 0.59)	<0.001
Democratic People's Republic of Korea	23.97 (17.49-32.31)	0.31 (0.21-0.42)	25.64 (18.67-34.27)	0.35 (0.24-0.48)	1.02 (0.94 to 1.09)	<0.001
Democratic Republic of the Congo	34.19 (23.21-47.34)	0.18 (0.12-0.26)	86.8 (59.22-124.12)	0.19 (0.13-0.28)	0.62 (0.57 to 0.67)	<0.001
Denmark	153.88 (135.25-172.98)	10.31 (8.87-11.91)	161.85 (140.65-184.54)	11.14 (9.42-13.06)	-0.01 (-0.18 to 0.17)	0.951

Djibouti	0.44 (0.31-0.62)	0.18 (0.12-0.26)	0.91 (0.63-1.25)	0.19 (0.12-0.27)	0.23 (0.2 to 0.26)	<0.001
Dominica	0.14 (0.11-0.19)	0.43 (0.3-0.58)	0.12 (0.09-0.15)	0.49 (0.35-0.66)	0.90 (0.84 to 0.96)	<0.001
Dominican Republic	13.86 (10.2-18.73)	0.4 (0.28-0.55)	18.42 (13.47-24.66)	0.45 (0.32-0.62)	0.56 (0.53 to 0.59)	<0.001
Ecuador	13.49 (10.23-17.64)	0.27 (0.2-0.37)	25.42 (19.88-32.02)	0.36 (0.27-0.48)	1.19 (1.08 to 1.3)	<0.001
Egypt	219.18 (170.38-276.7)	0.85 (0.63-1.1)	321.92 (236.46-441.71)	0.77 (0.55-1.08)	-0.10 (-0.19 to -0.01)	0.035
El Salvador	11.08 (8.12-14.78)	0.41 (0.29-0.56)	10.02 (7.37-13.5)	0.41 (0.29-0.57)	0.29 (0.2 to 0.37)	<0.001
Equatorial Guinea	0.35 (0.24-0.5)	0.17 (0.11-0.25)	1.95 (1.4-2.64)	0.25 (0.17-0.35)	2.12 (2.07 to 2.17)	<0.001
Eritrea	2.61 (1.77-3.6)	0.17 (0.11-0.24)	6.34 (4.3-8.78)	0.19 (0.12-0.27)	0.75 (0.65 to 0.86)	<0.001
Estonia	5.50 (4.44-6.85)	1.16 (0.9-1.47)	4.04 (3.12-5.12)	1.45 (1.09-1.89)	0.64 (0.54 to 0.75)	<0.001
Eswatini	0.90 (0.62-1.27)	0.21 (0.14-0.3)	1.23 (0.86-1.69)	0.23 (0.15-0.32)	0.70 (0.67 to 0.73)	<0.001
Ethiopia	37.22 (25.21-52.37)	0.14 (0.09-0.21)	89.45 (60.54-124.43)	0.16 (0.1-0.23)	0.71 (0.68 to 0.75)	<0.001
Fiji	0.50 (0.34-0.69)	0.14 (0.09-0.2)	0.57 (0.4-0.81)	0.17 (0.11-0.24)	0.65 (0.62 to 0.68)	<0.001
Finland	104.00 (94.26-114.23)	7.81 (6.95-8.7)	85.68 (69.42-106.29)	6.64 (5.23-8.4)	-0.37 (-0.76 to 0.03)	0.068
France	1024.12 (941.92-1105.18)	5.72 (5.16-6.32)	1175.72 (1000.71-1324.25)	6.62 (5.45-7.68)	0.46 (0.36 to 0.57)	<0.001
Gabon	0.97 (0.67-1.32)	0.21 (0.14-0.29)	1.87 (1.28-2.56)	0.24 (0.16-0.34)	0.97 (0.96 to 0.99)	<0.001
Gambia	0.82 (0.55-1.13)	0.17 (0.11-0.24)	2.29 (1.56-3.23)	0.20 (0.13-0.28)	1.01 (0.97 to 1.05)	<0.001
Georgia	18.61 (14.26-23.92)	1.00 (0.75-1.32)	8.92 (6.66-11.69)	1.00 (0.74-1.33)	-0.23 (-0.37 to -0.09)	0.001
Germany	565.16 (447.68-734.01)	3.04 (2.34-4.03)	761.18 (606.18-948.24)	4.38 (3.41-5.58)	1.38 (1.28 to 1.48)	<0.001
Ghana	13.97 (9.58-19.09)	0.19 (0.12-0.27)	30.95 (21.7-42.52)	0.21 (0.14-0.3)	0.80 (0.72 to 0.87)	<0.001
Greece	62.08 (49.36-78)	1.87 (1.44-2.4)	51.29 (42.23-61.54)	2.33 (1.88-2.87)	0.47 (0.32 to 0.62)	<0.001
Greenland	0.57 (0.43-0.74)	3.50 (2.62-4.6)	0.66 (0.5-0.84)	4.18 (3.15-5.49)	1.06 (0.88 to 1.24)	<0.001

Grenada	0.17 (0.12-0.22)	0.42 (0.29-0.58)	0.17 (0.12-0.22)	0.47 (0.33-0.65)	0.98 (0.93 to 1.03)	<0.001
Guam	0.08 (0.06-0.11)	0.15 (0.1-0.22)	0.11 (0.08-0.15)	0.18 (0.12-0.26)	0.68 (0.61 to 0.75)	<0.001
Guatemala	14.94 (11.07-20.04)	0.38 (0.27-0.52)	31.94 (23.56-42.77)	0.39 (0.28-0.54)	0.72 (0.67 to 0.77)	<0.001
Guinea	4.85 (3.22-6.87)	0.17 (0.11-0.25)	11.50 (7.93-15.83)	0.18 (0.12-0.25)	0.45 (0.41 to 0.49)	<0.001
Guinea-Bissau	0.90 (0.62-1.25)	0.18 (0.11-0.25)	1.80 (1.24-2.53)	0.19 (0.12-0.27)	0.53 (0.49 to 0.56)	<0.001
Guyana	1.60 (1.17-2.13)	0.42 (0.3-0.58)	1.40 (1.02-1.84)	0.46 (0.33-0.63)	0.51 (0.44 to 0.58)	<0.001
Haiti	10.99 (8.11-14.5)	0.37 (0.26-0.5)	21.55 (15.74-29.34)	0.40 (0.28-0.56)	0.59 (0.5 to 0.69)	<0.001
Honduras	9.38 (6.95-12.67)	0.39 (0.28-0.54)	16.98 (12.32-22.92)	0.37 (0.27-0.51)	0.42 (0.36 to 0.48)	<0.001
Hungary	167.56 (143.04-193.52)	4.98 (4.16-5.86)	159.77 (138.63-183.78)	7.67 (6.5-9.01)	1.29 (1.09 to 1.49)	<0.001
Iceland	2.37 (1.87-3.02)	2.64 (2.04-3.39)	3.84 (3.03-4.88)	4.09 (3.15-5.27)	1.48 (1.21 to 1.75)	<0.001
India	1203.02 (852.86-1685.94)	0.31 (0.21-0.45)	1599.45 (1131.06-2224.24)	0.28 (0.19-0.4)	0.30 (0.07 to 0.53)	0.01
Indonesia	107.33 (71.35-149.95)	0.12 (0.08-0.18)	141.94 (97.62-195.7)	0.15 (0.1-0.21)	0.95 (0.9 to 1.01)	<0.001
Iran (Islamic Republic of)	142.81 (103.08-197.39)	0.49 (0.34-0.7)	120.55 (87.66-167.04)	0.48 (0.34-0.68)	0.22 (-0.24 to 0.69)	0.343
Iraq	42.06 (31.74-55.79)	0.47 (0.34-0.63)	106.09 (79.56-144.57)	0.56 (0.41-0.78)	1.14 (1.11 to 1.18)	<0.001
Ireland	36.52 (28.89-45.91)	2.46 (1.9-3.17)	35.39 (28.02-44.5)	2.52 (1.94-3.22)	-0.14 (-0.38 to 0.1)	0.256
Israel	37.04 (29.63-46.02)	1.79 (1.4-2.28)	61.51 (50.5-75.18)	1.92 (1.53-2.39)	0.05 (-0.13 to 0.23)	0.558
Italy	828.87 (665.68-1026.29)	4.72 (3.72-5.93)	604.46 (490.34-741.6)	4.89 (3.87-6.12)	-0.30 (-0.38 to -0.22)	<0.001
Jamaica	4.52 (3.31-5.97)	0.40 (0.29-0.55)	4.48 (3.31-5.95)	0.45 (0.32-0.61)	0.78 (0.71 to 0.85)	<0.001
Japan	800.67 (610.25-1031.43)	1.92 (1.44-2.52)	1589.62 (1262.94-1983.16)	6.27 (4.91-7.96)	3.82 (3.57 to 4.07)	<0.001
Jordan	24.44 (19.42-30.46)	1.18 (0.91-1.51)	81.48 (63.06-105.17)	1.57 (1.18-2.06)	1.30 (1.13 to 1.47)	<0.001
Kazakhstan	63.06 (47.33-82.22)	0.97 (0.72-1.29)	59.31 (45.51-76.35)	1.06 (0.78-1.4)	-0.03 (-0.12 to 0.06)	0.524

Kenya	19.45 (13.35-27.08)	0.16 (0.1-0.22)	45.11 (31.35-62.15)	0.18 (0.12-0.25)	0.84 (0.8 to 0.88)	<0.001
Kiribati	0.04 (0.03-0.06)	0.13 (0.08-0.19)	0.07 (0.05-0.1)	0.14 (0.09-0.2)	0.54 (0.46 to 0.62)	<0.001
Kuwait	7.69 (6.6-8.9)	1.22 (1.01-1.44)	8.47 (6.36-11.2)	0.79 (0.58-1.08)	-1.11 (-1.72 to -0.49)	<0.001
Kyrgyzstan	18.28 (13.99-23.7)	0.93 (0.69-1.24)	22.88 (17.59-29.48)	0.95 (0.7-1.26)	0.02 (-0.1 to 0.14)	0.773
Lao People's Democratic Republic	2.66 (1.77-3.8)	0.13 (0.08-0.19)	4.88 (3.35-6.7)	0.16 (0.11-0.23)	1.14 (1.1 to 1.18)	<0.001
Latvia	11.93 (9.33-14.98)	1.54 (1.18-1.97)	8.37 (6.6-10.73)	2.12 (1.63-2.78)	1.00 (0.75 to 1.25)	<0.001
Lebanon	7.31 (5.44-9.89)	0.51 (0.36-0.71)	10.92 (8.04-14.54)	0.69 (0.49-0.95)	1.09 (0.82 to 1.36)	<0.001
Lesotho	1.78 (1.22-2.48)	0.19 (0.13-0.28)	1.99 (1.39-2.78)	0.21 (0.14-0.31)	0.69 (0.57 to 0.81)	<0.001
Liberia	1.79 (1.25-2.52)	0.18 (0.12-0.26)	4.63 (3.2-6.39)	0.19 (0.13-0.28)	0.60 (0.52 to 0.69)	<0.001
Libya	8.52 (6.3-11.74)	0.38 (0.27-0.54)	12.34 (9.1-16.65)	0.51 (0.37-0.71)	1.75 (1.6 to 1.9)	<0.001
Lithuania	19.47 (15.67-24.45)	1.67 (1.29-2.13)	15.28 (12.98-17.93)	2.60 (2.15-3.14)	1.57 (1.49 to 1.64)	<0.001
Luxembourg	3.45 (2.7-4.3)	3.66 (2.8-4.65)	5.48 (4.34-6.88)	3.82 (2.96-4.91)	0.13 (-0.13 to 0.39)	0.319
Madagascar	10.83 (7.51-15.06)	0.18 (0.12-0.26)	24.82 (17.3-34.36)	0.19 (0.12-0.26)	0.39 (0.36 to 0.42)	<0.001
Malawi	8.13 (5.47-11.49)	0.17 (0.11-0.25)	19.02 (12.99-26.58)	0.18 (0.12-0.26)	0.79 (0.73 to 0.85)	<0.001
Malaysia	13.26 (9.73-17.48)	0.16 (0.12-0.22)	29.49 (22.75-37.73)	0.26 (0.19-0.35)	2.00 (1.92 to 2.08)	<0.001
Maldives	0.14 (0.09-0.2)	0.13 (0.08-0.18)	0.22 (0.16-0.31)	0.17 (0.11-0.23)	1.15 (0.98 to 1.33)	<0.001
Mali	6.65 (4.41-9.51)	0.16 (0.1-0.24)	20.03 (13.87-28.13)	0.17 (0.11-0.25)	0.51 (0.44 to 0.57)	<0.001
Malta	2.64 (2.03-3.38)	2.16 (1.62-2.87)	2.23 (1.77-2.86)	2.54 (1.95-3.28)	0.51 (0.47 to 0.55)	<0.001
Marshall Islands	0.03 (0.02-0.04)	0.13 (0.08-0.18)	0.04 (0.02-0.05)	0.15 (0.1-0.21)	0.96 (0.87 to 1.04)	<0.001
Mauritania	1.93 (1.32-2.68)	0.19 (0.13-0.28)	4.12 (2.89-5.59)	0.20 (0.13-0.28)	0.59 (0.53 to 0.65)	<0.001
Mauritius	0.7 (0.47-0.97)	0.15 (0.1-0.22)	0.68 (0.48-0.93)	0.19 (0.13-0.26)	1.05 (1.02 to 1.09)	<0.001
Mexico	214.72 (160.8-279.56)	0.49 (0.36-0.65)	220.56 (167.51-285.08)	0.47 (0.35-0.63)	0.18 (0.04 to 0.32)	0.009

Micronesia (Federated States of)	0.07 (0.05-0.1)	0.13 (0.08-0.19)	0.07 (0.05-0.1)	0.16 (0.1-0.22)	1.09 (1.05 to 1.13)	<0.001
Monaco	0.11 (0.09-0.14)	2.04 (1.53-2.7)	0.16 (0.12-0.21)	2.10 (1.58-2.77)	0.14 (0.03 to 0.25)	0.016
Mongolia	9.87 (7.51-12.78)	0.93 (0.69-1.23)	10.62 (8.01-13.68)	1.02 (0.75-1.35)	-0.04 (-0.15 to 0.07)	0.424
Montenegro	5.99 (4.64-7.66)	2.63 (1.99-3.43)	4.49 (3.48-5.68)	2.65 (2.01-3.41)	0.25 (0.2 to 0.31)	<0.001
Morocco	44.32 (33.03-58.11)	0.37 (0.26-0.49)	53.61 (39.63-71.12)	0.40 (0.29-0.55)	0.64 (0.62 to 0.67)	<0.001
Mozambique	10.32 (6.95-14.42)	0.16 (0.1-0.22)	26.87 (17.86-37.94)	0.17 (0.11-0.25)	0.46 (0.42 to 0.51)	<0.001
Myanmar	25.82 (17.01-36.42)	0.13 (0.09-0.2)	35.98 (24.32-49.53)	0.17 (0.11-0.25)	1.14 (1.06 to 1.21)	<0.001
Namibia	1.51 (1.04-2.14)	0.21 (0.14-0.3)	2.4 (1.68-3.31)	0.22 (0.15-0.32)	0.36 (0.35 to 0.38)	<0.001
Nauru	0.01 (0-0.01)	0.14 (0.09-0.2)	0.01 (0.01-0.01)	0.16 (0.1-0.23)	0.94 (0.91 to 0.98)	<0.001
Nepal	24.74 (17.57-34.98)	0.27 (0.18-0.39)	44.68 (31.05-62.09)	0.32 (0.22-0.46)	1.42 (1.32 to 1.53)	<0.001
Netherlands	226.2 (201.61-253.36)	5.12 (4.45-5.92)	63.91 (49.94-81)	1.49 (1.14-1.93)	-4.41 (-4.79 to -4.04)	<0.001
New Zealand	39.43 (31.26-49.76)	3.16 (2.47-4.02)	54.05 (44.18-66.11)	4.25 (3.41-5.32)	0.87 (0.77 to 0.96)	<0.001
Nicaragua	7.94 (5.72-10.53)	0.39 (0.27-0.53)	10.19 (7.37-13.31)	0.37 (0.26-0.5)	0.35 (0.29 to 0.42)	<0.001
Niger	6.27 (4.19-8.87)	0.16 (0.1-0.23)	19.91 (13.44-27.98)	0.16 (0.1-0.23)	0.18 (0.09 to 0.26)	<0.001
Nigeria	70.28 (49.01-98.03)	0.16 (0.11-0.23)	199.07 (139.13-274.81)	0.18 (0.12-0.25)	0.54 (0.43 to 0.66)	<0.001
Niue	0.00 (0-0)	0.14 (0.09-0.2)	0.00 (0-0)	0.17 (0.11-0.25)	1.05 (0.96 to 1.14)	<0.001
North Macedonia	19.17 (14.84-24.37)	2.57 (1.94-3.33)	13.55 (10.52-17.27)	2.65 (2.01-3.44)	0.23 (0.15 to 0.32)	<0.001
Northern Mariana Islands	0.03 (0.02-0.03)	0.15 (0.1-0.22)	0.03 (0.02-0.04)	0.18 (0.12-0.25)	1.37 (1.1 to 1.64)	<0.001
Norway	52.05 (40.46-67.61)	4.03 (3.07-5.27)	92.24 (73.44-115.84)	6.66 (5.15-8.47)	1.5 (1.39 to 1.62)	<0.001
Oman	4.54 (3.4-6.07)	0.55 (0.39-0.75)	6.93 (5.13-9.17)	0.59 (0.43-0.81)	0.54 (0.38 to 0.7)	<0.001
Pakistan	139.34 (98.71-195.21)	0.25 (0.17-0.36)	305.61 (215.89-431.11)	0.28 (0.19-0.4)	0.72 (0.68 to 0.75)	<0.001
Palau	0.01 (0.01-0.01)	0.15 (0.1-0.21)	0.01 (0.01-0.01)	0.18 (0.12-0.25)	0.74 (0.68 to 0.81)	<0.001

Palestine	5.09<br>(3.82-6.75)	0.48<br>(0.35-0.67)	14.32<br>(10.57-19.48)	0.61<br>(0.43-0.84)	1.26<br>(1.15 to 1.38)	<0.001
Panama	5.23<br>(3.98-6.69)	0.47<br>(0.35-0.62)	6.32<br>(4.62-8.45)	0.41<br>(0.29-0.56)	-0.43<br>(-0.55 to -0.31)	<0.001
Papua New Guinea	2.27<br>(1.47-3.2)	0.11<br>(0.07-0.17)	5.62<br>(3.7-7.82)	0.13<br>(0.08-0.19)	0.38<br>(0.33 to 0.43)	<0.001
Paraguay	12.82<br>(9.77-16.52)	0.68<br>(0.5-0.9)	15.51<br>(11.42-20.54)	0.56<br>(0.4-0.76)	-0.11<br>(-0.3 to 0.07)	0.236
Peru	28.14<br>(19.9-38.19)	0.27<br>(0.18-0.37)	33.49<br>(24.19-45.67)	0.27<br>(0.19-0.38)	0.24<br>(0.17 to 0.32)	<0.001
Philippines	41.23<br>(28.26-56.76)	0.13<br>(0.09-0.19)	74.56<br>(52.02-101.91)	0.16<br>(0.11-0.23)	0.83<br>(0.71 to 0.96)	<0.001
Poland	502.63<br>(389.44-646.01)	3.9<br>(2.95-5.09)	377.79<br>(310.44-462.38)	4.71<br>(3.78-5.83)	0.63<br>(0.53 to 0.74)	<0.001
Portugal	48.00<br>(38.08-60.97)	1.33<br>(1.02-1.73)	59.07<br>(48.11-71.93)	2.47<br>(1.95-3.08)	2.21<br>(1.95 to 2.46)	<0.001
Puerto Rico	10.86<br>(9.07-13.15)	0.75<br>(0.61-0.94)	6.3<br>(5.08-7.78)	0.67<br>(0.52-0.86)	0.09<br>(-0.01 to 0.19)	0.07
Qatar	0.81<br>(0.6-1.1)	0.61<br>(0.44-0.85)	3.48<br>(2.65-4.61)	0.71<br>(0.52-0.97)	0.79<br>(0.72 to 0.87)	<0.001
Republic of Korea	210.62<br>(170.75-259.02)	1.13<br>(0.89-1.42)	332.82<br>(299.81-369.59)	3.08<br>(2.71-3.5)	3.23<br>(2.85 to 3.61)	<0.001
Republic of Moldova	14.82<br>(11.32-19.44)	0.96<br>(0.71-1.27)	10.85<br>(8.88-13.13)	1.28<br>(1.02-1.6)	1.35<br>(1.24 to 1.47)	<0.001
Romania	146.1<br>(112.11-188.89)	1.76<br>(1.31-2.3)	93.18<br>(74.85-115.06)	2.09<br>(1.64-2.62)	0.67<br>(0.4 to 0.94)	<0.001
Russian Federation	436.68<br>(334.29-561.32)	0.95<br>(0.71-1.24)	328.92<br>(251.92-423.43)	0.98<br>(0.73-1.29)	-0.06<br>(-0.22 to 0.1)	0.452
Rwanda	6.36<br>(4.28-8.94)	0.18<br>(0.11-0.26)	11.72<br>(8.04-16.18)	0.19<br>(0.12-0.27)	0.69<br>(0.58 to 0.81)	<0.001
Saint Kitts and Nevis	0.08<br>(0.06-0.11)	0.44<br>(0.31-0.61)	0.09<br>(0.07-0.12)	0.49<br>(0.35-0.67)	0.73<br>(0.7 to 0.77)	<0.001
Saint Lucia	0.29<br>(0.21-0.39)	0.43<br>(0.3-0.59)	0.25<br>(0.18-0.34)	0.46<br>(0.33-0.65)	0.9<br>(0.81 to 0.98)	<0.001
Saint Vincent and the Grenadines	0.23<br>(0.17-0.3)	0.41<br>(0.29-0.56)	0.17<br>(0.13-0.23)	0.45<br>(0.32-0.61)	0.65<br>(0.57 to 0.73)	<0.001
Samoa	0.12<br>(0.08-0.16)	0.13<br>(0.08-0.19)	0.16<br>(0.11-0.22)	0.15<br>(0.1-0.21)	0.51<br>(0.47 to 0.55)	<0.001
San Marino	0.15<br>(0.12-0.19)	1.92<br>(1.45-2.49)	0.18<br>(0.14-0.23)	2.05<br>(1.53-2.69)	0.11<br>(0.06 to 0.15)	<0.001
Sao Tome and Principe	0.11<br>(0.08-0.16)	0.18<br>(0.11-0.25)	0.2<br>(0.15-0.28)	0.2<br>(0.14-0.28)	0.89<br>(0.83 to 0.95)	<0.001
Saudi Arabia	48.4<br>(37.95-61.73)	0.63<br>(0.48-0.84)	71.36<br>(53.62-92.36)	0.66<br>(0.49-0.88)	0.86<br>(0.75 to 0.97)	<0.001

Senegal	7.03<br>(4.83-9.91)	0.18<br>(0.12-0.26)	14.84<br>(10.37-20.66)	0.2<br>(0.13-0.28)	0.62<br>(0.58 to 0.66)	<0.001
Serbia	76.63<br>(58.84-98.03)	2.50<br>(1.87-3.24)	75.83<br>(60.18-95.58)	3.40<br>(2.64-4.36)	1.14<br>(1.02 to 1.26)	<0.001
Seychelles	0.05<br>(0.04-0.07)	0.16<br>(0.1-0.23)	0.06<br>(0.04-0.08)	0.19<br>(0.13-0.27)	0.58<br>(0.51 to 0.65)	<0.001
Sierra Leone	2.98<br>(2.05-4.21)	0.18<br>(0.12-0.26)	7.77<br>(5.35-10.76)	0.19<br>(0.12-0.27)	0.58<br>(0.53 to 0.62)	<0.001
Singapore	11.79<br>(8.91-15.36)	1.08<br>(0.8-1.44)	11.08<br>(8.44-14.56)	1.05<br>(0.77-1.43)	-0.75<br>(-1.22 to -0.27)	0.002
Slovakia	77.98<br>(63.56-94.76)	4.12<br>(3.25-5.09)	48.09<br>(38.8-58.56)	4.16<br>(3.26-5.16)	-0.09<br>(-0.25 to 0.07)	0.291
Slovenia	23.79<br>(19.31-29.49)	3.84<br>(3.04-4.84)	19.28<br>(15.69-23.64)	4.71<br>(3.72-5.88)	0.33<br>(0.21 to 0.45)	<0.001
Solomon Islands	0.21<br>(0.13-0.3)	0.12<br>(0.07-0.17)	0.41<br>(0.27-0.59)	0.13<br>(0.08-0.19)	0.54<br>(0.5 to 0.59)	<0.001
Somalia	5.65<br>(3.8-7.9)	0.15<br>(0.09-0.21)	15.01<br>(9.97-21.47)	0.14<br>(0.09-0.2)	-0.14<br>(-0.25 to -0.03)	0.011
South Africa	33.78<br>(23.6-46.18)	0.19<br>(0.13-0.27)	39.9<br>(28.14-54.73)	0.2<br>(0.13-0.28)	0.14<br>(0.06 to 0.22)	<0.001
South Sudan	5.33<br>(3.61-7.4)	0.17<br>(0.11-0.25)	9.14<br>(6.39-12.61)	0.18<br>(0.12-0.26)	0.23<br>(0.15 to 0.3)	<0.001
Spain	627.53<br>(569.58-692.81)	4.58<br>(4.03-5.19)	615.84<br>(553.09-685.7)	6.21<br>(5.46-7.08)	0.64<br>(0.44 to 0.84)	<0.001
Sri Lanka	15.29<br>(10.82-20.65)	0.2<br>(0.14-0.28)	27.89<br>(21.56-35.56)	0.37<br>(0.28-0.49)	2.34<br>(2.17 to 2.51)	<0.001
Sudan	48.88<br>(36.14-66.55)	0.49<br>(0.35-0.69)	116.73<br>(86.96-154.42)	0.58<br>(0.42-0.79)	0.95<br>(0.91 to 1)	<0.001
Suriname	0.74<br>(0.55-0.97)	0.43<br>(0.3-0.58)	0.94<br>(0.69-1.25)	0.46<br>(0.33-0.63)	0.45<br>(0.36 to 0.54)	<0.001
Sweden	104.36<br>(82.67-130.75)	4.48<br>(3.47-5.71)	109.88<br>(87.41-136.88)	4.44<br>(3.43-5.64)	-0.29<br>(-0.49 to -0.09)	0.004
Switzerland	61.46<br>(49.16-77.53)	3.52<br>(2.78-4.53)	75.15<br>(61.28-93.77)	4.08<br>(3.22-5.17)	0.34<br>(0.29 to 0.39)	<0.001
Syrian Arab Republic	38.13<br>(28.38-50.88)	0.56<br>(0.4-0.77)	46.86<br>(34.79-62.86)	0.64<br>(0.46-0.88)	1.57<br>(1.48 to 1.66)	<0.001
Taiwan (Province of China)	20.55<br>(15.51-26.49)	0.26<br>(0.19-0.34)	29.33<br>(23.32-36.35)	0.6<br>(0.46-0.76)	3.13<br>(2.82 to 3.44)	<0.001
Tajikistan	22.57<br>(17.33-29.18)	0.89<br>(0.66-1.19)	36.06<br>(27.44-47.19)	0.93<br>(0.69-1.23)	0.33<br>(0.23 to 0.43)	<0.001
Thailand	30.02<br>(19.97-42.42)	0.12<br>(0.07-0.18)	28.92<br>(19.98-39.15)	0.17<br>(0.11-0.24)	1.38<br>(1.26 to 1.49)	<0.001
Timor-Leste	0.44<br>(0.3-0.61)	0.13<br>(0.08-0.18)	1.11<br>(0.76-1.54)	0.16<br>(0.1-0.23)	1.54<br>(1.5 to 1.57)	<0.001

Togo	3.40 (2.37-4.71)	0.18 (0.12-0.26)	7.56 (5.19-10.58)	0.20 (0.13-0.29)	0.66 (0.58 to 0.75)	<0.001
Tokelau	0 (0-0)	0.14 (0.09-0.2)	0 (0-0)	0.16 (0.11-0.23)	0.83 (0.53 to 1.12)	<0.001
Tonga	0.07 (0.04-0.09)	0.13 (0.08-0.19)	0.07 (0.05-0.1)	0.16 (0.1-0.23)	0.67 (0.62 to 0.73)	<0.001
Trinidad and Tobago	2.39 (1.78-3.23)	0.47 (0.33-0.64)	1.97 (1.47-2.66)	0.51 (0.36-0.7)	0.55 (0.42 to 0.68)	<0.001
Tunisia	21.75 (16.28-29.31)	0.55 (0.4-0.76)	21.84 (16.19-29.23)	0.61 (0.44-0.85)	0.61 (0.53 to 0.68)	<0.001
Turkey	302.1 (241.72-373.44)	1.07 (0.82-1.35)	415.33 (323.21-523.72)	1.57 (1.19-2.01)	1.87 (1.64 to 2.1)	<0.001
Turkmenistan	16.88 (12.88-21.87)	0.98 (0.72-1.29)	19.18 (14.68-24.63)	1.06 (0.79-1.4)	0.40 (0.3 to 0.51)	<0.001
Tuvalu	0 (0-0.01)	0.13 (0.08-0.19)	0.01 (0.01-0.01)	0.16 (0.1-0.23)	1.28 (1.22 to 1.34)	<0.001
Uganda	14.66 (9.87-20.61)	0.16 (0.11-0.24)	42.02 (29.16-57.7)	0.19 (0.12-0.27)	0.73 (0.65 to 0.81)	<0.001
Ukraine	113.77 (85.47-148.66)	0.72 (0.53-0.96)	69.89 (53.29-90.55)	0.76 (0.56-1)	0.12 (-0.04 to 0.27)	0.136
United Arab Emirates	3.74 (2.78-4.93)	0.65 (0.47-0.87)	11.80 (9.02-15.58)	0.75 (0.55-1.02)	1.38 (0.78 to 1.99)	<0.001
United Kingdom	725.27 (590.37-891.43)	4.42 (3.52-5.53)	851.01 (690.96-1042.7)	5.24 (4.17-6.54)	0.48 (0.32 to 0.64)	<0.001
United Republic of Tanzania	23.24 (16.09-32.25)	0.17 (0.11-0.25)	53.25 (36.71-73.76)	0.19 (0.12-0.27)	0.38 (0.36 to 0.4)	<0.001
United States of America	4600.96 (3638.27-5736.17)	5.93 (4.62-7.5)	6042.14 (5148.39-7114.53)	6.55 (5.4-7.88)	0.53 (0.44 to 0.62)	<0.001
United States Virgin Islands	0.19 (0.15-0.26)	0.45 (0.33-0.62)	0.14 (0.11-0.19)	0.51 (0.36-0.69)	0.61 (0.52 to 0.7)	<0.001
Uruguay	3.97 (2.88-5.2)	0.35 (0.25-0.48)	4.27 (3.12-5.61)	0.41 (0.29-0.56)	0.65 (0.61 to 0.69)	<0.001
Uzbekistan	86.03 (65.45-111.73)	0.89 (0.66-1.18)	127.67 (96.78-166.26)	1 (0.75-1.33)	0.61 (0.45 to 0.77)	<0.001
Vanuatu	0.09 (0.06-0.13)	0.12 (0.08-0.18)	0.19 (0.13-0.27)	0.14 (0.09-0.21)	0.73 (0.66 to 0.81)	<0.001
Venezuela (Bolivarian Republic of)	41 (30.8-52.98)	0.46 (0.34-0.62)	39.76 (29.36-52.83)	0.41 (0.29-0.55)	-0.23 (-0.45 to -0.01)	0.037
Viet Nam	78.22 (53.27-107.53)	0.24 (0.16-0.34)	119.4 (85.47-161.19)	0.41 (0.28-0.56)	1.99 (1.71 to 2.27)	<0.001
Yemen	32.52 (24.28-43.7)	0.47 (0.34-0.65)	85.1 (62.23-113.5)	0.54 (0.39-0.75)	1.05 (1 to 1.1)	<0.001

Zambia	8.14 (5.6-11.27)	0.19 (0.13-0.27)	18.79 (13-25.86)	0.20 (0.13-0.28)	0.35 (0.32 to 0.38)	<0.001
Zimbabwe	11.48 (8.02-16.02)	0.2 (0.13-0.29)	15.41 (10.71-21.57)	0.21 (0.14-0.3)	0.18 (0.13 to 0.23)	<0.001

Supplementary table 5. Cases and age-standardized for IBD DALYs and their average annual percentage changes (AAPCs) from 1990 to 2019 at the national levels.

	1990		2019		AAPCs, 1990-2019 (95% CI)	P value
	cases (n)	ASDR (per 100,000 population, 95% CI)	cases (n)	ASDR (per 100,000 population, 95% CI)		
Afghanistan	170.63 (31.14-804.84)	2.5 (0.43-11.62)	394.35 (156.76-1014.05)	1.81 (0.61-4.9)	-1.05 (-1.99 to -0.09)	0.033
Albania	3052.61 (464.31-4368.86)	207.48 (30.59-305.05)	236.23 (118.78-416.57)	38.26 (16.47-71.49)	-5.65 (-6.78 to -4.5)	<0.001
Algeria	190.13 (85.51-601.42)	1.44 (0.56-4.76)	176.37 (106.76-314.2)	1.22 (0.62-2.35)	-0.54 (-0.77 to -0.31)	<0.001
American Samoa	2.13 (1.1-3.84)	8.21 (3.47-16.23)	0.71 (0.38-1.27)	3.27 (1.32-6.78)	-3.53 (-4.01 to -3.06)	<0.001
Andorra	0.76 (0.41-1.35)	6.19 (2.44-13.03)	0.45 (0.3-0.67)	3.13 (1.65-5.46)	-2.25 (-2.74 to -1.77)	<0.001
Angola	980.56 (118.09-3381.22)	14.22 (1.57-49.43)	1327.39 (520.82-2821.83)	7.36 (2.31-16.8)	-2.71 (-2.93 to -2.48)	<0.001
Antigua and Barbuda	1.89 (1.17-2.69)	7.75 (4.47-12.36)	1.36 (0.9-2.08)	6.28 (3.64-10.42)	-0.98 (-1.5 to -0.45)	<0.001
Argentina	211.99 (166.67-257.64)	1.64 (1.13-2.32)	175.69 (139.6-218.83)	1.20 (0.83-1.69)	-0.87 (-1.21 to -0.52)	<0.001
Armenia	20.43 (12.35-46.87)	1.54 (0.81-3.64)	11.71 (6.39-27.56)	1.54 (0.75-3.87)	-0.02 (-0.26 to 0.21)	0.843
Australia	230.63 (171.59-297.3)	4.4 (2.74-6.36)	277.42 (182.49-385.35)	4.54 (2.45-7.13)	0.15 (-0.04 to 0.33)	0.114
Austria	315.49 (212.08-419.33)	17.19 (10.11-25.02)	125.04 (91.18-168.52)	6.90 (4.32-10.22)	-2.88 (-3.22 to -2.54)	<0.001
Azerbaijan	61.13 (38.32-123.43)	1.93 (0.97-4.3)	44.4 (27.98-66.22)	1.49 (0.76-2.72)	-0.99 (-1.3 to -0.68)	<0.001
Bahamas	6.77 (4.71-9.71)	6.70 (3.98-10.66)	3.83 (2.74-5.38)	3.64 (2.23-5.58)	-2.05 (-2.89 to -1.21)	<0.001
Bahrain	3.45 (2.5-4.69)	1.84 (1.09-2.93)	5.22 (3.33-7.89)	1.50 (0.82-2.52)	-0.18 (-0.77 to 0.42)	0.556
Bangladesh	7610.19 (1315.03-17775.73)	11.88 (1.8-30.45)	2617.94 (1168.23-4561.52)	4.42 (1.48-10.17)	-3.57 (-3.88 to -3.25)	<0.001

Barbados	3.81 (2.76-5.02)	4.56 (2.76-7.09)	1.70 (1.27-2.21)	2.33 (1.48-3.48)	-2.26 (-2.78 to -1.73)	<0.001
Belarus	100.87 (71.43-142.08)	3.24 (1.91-5.16)	51.05 (33.17-75.09)	2.49 (1.44-4.04)	-0.96 (-1.14 to -0.78)	<0.001
Belgium	122.35 (93.54-157.08)	4.81 (3.23-7)	81.86 (59.36-112.18)	3.15 (1.97-4.83)	-1.53 (-2.07 to -0.99)	<0.001
Belize	17.65 (10.64-28.32)	16.94 (9.15-28.89)	8.75 (6.31-11.78)	5.44 (3.34-8.25)	-4.3 (-4.55 to -4.04)	<0.001
Benin	339.18 (37.49-924.16)	10 (1.04-28.21)	477.8 (121.16-1147.37)	6.24 (1.31-16.07)	-1.92 (-2.27 to -1.56)	<0.001
Bermuda	1.33 (0.79-2.05)	8.24 (4.36-13.68)	0.35 (0.24-0.54)	3.2 (1.9-5.15)	-3.51 (-4.18 to -2.84)	<0.001
Bhutan	22.65 (2.8-66.52)	6.6 (0.65-21.43)	9.07 (3.93-18.62)	3.48 (1.07-8.3)	-2.89 (-3.22 to -2.55)	<0.001
Bolivia (Plurinational State of)	1114.69 (223.44-2863.47)	29.31 (4.95-77.94)	372.14 (172.66-680.37)	6.96 (2.54-14.12)	-4.97 (-5.26 to -4.69)	<0.001
Bosnia and Herzegovina	61.65 (38.13-131.07)	3.97 (2-9.89)	27.01 (17.12-39.15)	3.63 (2.01-6.68)	-0.08 (-0.53 to 0.37)	0.714
Botswana	62.08 (21.17-138.67)	8.63 (2.19-20.96)	108.24 (24.38-294.91)	12.19 (2.13-35.07)	1.13 (-0.78 to 3.07)	0.248
Brazil	3414.55 (2803.81-4279.46)	5.24 (4-7.01)	2211.07 (1828.53-2626.72)	3.44 (2.67-4.35)	-1.25 (-1.5 to -0.99)	<0.001
Brunei Darussalam	6.45 (3.9-9.86)	5.56 (2.74-9.98)	4.65 (3.44-6.15)	3.35 (1.99-5.34)	-1.57 (-1.78 to -1.36)	<0.001
Bulgaria	308.56 (164.49-441.34)	14.3 (6.34-22.52)	105.55 (54.19-144.64)	8.36 (3.59-13.05)	-2.00 (-2.88 to -1.11)	<0.001
Burkina Faso	910.57 (83.36-2517.46)	13.78 (1.15-39.11)	1584.92 (257.06-3918.37)	11.14 (1.53-29.3)	-0.77 (-1.35 to -0.19)	0.01
Burundi	606.94 (73.7-1794.7)	15.88 (1.81-48.22)	527.65 (198.49-1277.55)	7.37 (2.16-18.76)	-3.04 (-4.21 to -1.86)	<0.001
Cabo Verde	6.15 (2.12-12.71)	3.15 (0.95-7.26)	3.49 (2.06-5.79)	1.66 (0.73-3.46)	-2.14 (-3.12 to -1.15)	<0.001
Cambodia	542.09 (115.27-1418.99)	8.53 (1.61-23.33)	221.05 (110.88-415.83)	3.38 (1.31-7.58)	-3.38 (-3.73 to -3.03)	<0.001
Cameroon	418.64 (71.86-1008.77)	6.42 (1.01-16.31)	717.84 (223.32-1590.21)	4.70 (1.06-11.67)	-1.29 (-1.59 to -0.99)	<0.001
Canada	1300.21 (938.12-1796.68)	16.31 (10.39-24.03)	1044.16 (718.41-1458.04)	12.14 (7.46-18.29)	-0.96 (-1.15 to -0.77)	<0.001
Central African Republic	185.41 (48.38-484.56)	10.83 (2.34-29.9)	252.58 (74.08-648.54)	8.48 (1.93-23.11)	-1.05 (-1.32 to -0.79)	<0.001
Chad	394.91 (38.79-1070.9)	9.38 (0.91-26.25)	776.91 (142.77-2009.93)	6.95 (1.11-18.95)	-1.23 (-1.55 to -0.92)	<0.001

Chile	146.53 (101.56-183.82)	2.68 (1.64-3.86)	72.54 (55.6-93.36)	1.42 (0.95-2.04)	-2.00 (-2.42 to -1.58)	<0.001
China	92808.85 (39116.79-142824.35)	21.58 (8.8-34.37)	12328.45 (8911.49-15755.03)	4.09 (2.83-5.47)	-5.65 (-6.07 to -5.23)	<0.001
Colombia	713.91 (466.16-963.28)	4.63 (2.86-6.55)	483.27 (331.68-675.38)	3.22 (1.96-4.96)	-1.24 (-1.53 to -0.95)	<0.001
Comoros	27.06 (3.53-71.97)	9.33 (0.98-26.29)	18.52 (8.26-36.22)	6.22 (2.02-14.46)	-1.68 (-2.11 to -1.25)	<0.001
Congo	135.73 (45.94-311.47)	9.06 (2.54-21.79)	115.61 (49.63-257.65)	4.6 (1.52-11.57)	-2.53 (-2.81 to -2.24)	<0.001
Cook Islands	1.06 (0.52-1.88)	12.01 (4.47-26.01)	0.18 (0.07-0.38)	3.26 (0.91-8.27)	-4.38 (-4.71 to -4.04)	<0.001
Costa Rica	12.76 (8.91-20.9)	0.91 (0.56-1.58)	11.17 (7.23-18.52)	0.75 (0.45-1.4)	-0.32 (-0.59 to -0.04)	0.024
Croatia	86.59 (61.64-120.54)	6.19 (3.92-9.61)	50.43 (33.71-70.1)	5.67 (3.42-8.44)	-0.19 (-0.44 to 0.06)	0.133
Cuba	51.52 (35.72-78.47)	1.29 (0.8-2.13)	17.21 (11.3-27.66)	0.65 (0.38-1.14)	-2.42 (-2.88 to -1.96)	<0.001
Cyprus	17.15 (9.71-28.64)	6.91 (3.01-12.92)	8.35 (5.61-12.1)	2.97 (1.72-4.95)	-2.87 (-3.07 to -2.66)	<0.001
Czechia	138.56 (108.97-187.45)	4.39 (3.03-6.85)	86.03 (58.96-120.6)	4.01 (2.5-6.11)	-0.70 (-1.73 to 0.34)	0.188
C 么 te d'Ivoire	480.32 (76.92-1202.87)	6.18 (0.89-16.69)	626.41 (188.02-1344.54)	4.52 (1.08-10.84)	-1.26 (-1.93 to -0.59)	<0.001
Democratic People's Republic of Korea	1598.94 (409.54-3973.03)	15.93 (3.55-40.94)	324.40 (160-669.43)	5.09 (2.02-11.77)	-4.62 (-4.99 to -4.25)	<0.001
Democratic Republic of the Congo	3093.78 (594.22-8916.84)	11.79 (2.08-35.06)	2680.22 (980.06-6528.69)	5.52 (1.59-14.69)	-3.08 (-3.33 to -2.82)	<0.001
Denmark	101.12 (66.86-143.95)	6.82 (4.16-10.73)	88.03 (55.4-128.8)	6.02 (3.48-9.56)	-0.66 (-0.92 to -0.4)	<0.001
Djibouti	30.2 (4.85-89.07)	9.42 (1.32-28.23)	41.68 (12.26-93.69)	7.34 (1.87-17.33)	-1.16 (-1.85 to -0.47)	0.001
Dominica	2.63 (1.67-4.27)	8.18 (3.98-15.58)	1.23 (0.75-1.99)	6.45 (2.87-12.64)	-0.95 (-1.61 to -0.29)	0.005
Dominican Republic	285.28 (114.49-591.77)	7.59 (2.58-16.53)	159.86 (77.91-318.61)	3.96 (1.48-8.96)	-2.49 (-3.48 to -1.48)	<0.001
Ecuador	499.23 (284.97-760.58)	9.81 (5.04-16.34)	215.34 (126.67-325.23)	3.26 (1.7-5.8)	-3.68 (-4.2 to -3.16)	<0.001
Egypt	449.67 (209.82-806.18)	1.59 (0.64-3.27)	481.36 (298.08-763.49)	1.16 (0.59-2.17)	-1.14 (-1.5 to -0.78)	<0.001
El Salvador	267.88 (129.14-461.75)	9.77 (4.28-18.19)	23.70 (14.28-38.12)	1.03 (0.47-2.01)	-7.53 (-8.07 to -6.99)	<0.001

Equatorial Guinea	26.53 (6.21-75.48)	9.01 (1.85-25.57)	23.13 (10.47-50.06)	3.15 (1.07-7.99)	-4.27 (-5.02 to -3.5)	<0.001
Eritrea	268.59 (29.08-859.89)	13.47 (1.39-43.55)	331.54 (117.83-781.57)	9.67 (2.73-24.21)	-1.54 (-1.98 to -1.09)	<0.001
Estonia	52.23 (28.46-76.4)	11.50 (5.65-17.86)	9.56 (5.76-12.88)	3.54 (1.81-5.35)	-4.03 (-4.2 to -3.86)	<0.001
Eswatini	55.15 (10.48-155.29)	10.6 (1.67-30.53)	44.33 (20.59-86.6)	8.44 (2.94-18.17)	-1.32 (-2.08 to -0.55)	0.001
Ethiopia	6407.55 (869.49-18074.85)	18.31 (2.51-52.46)	4248.58 (1886.29-8424.55)	7.01 (2.81-14.52)	-3.63 (-4.45 to -2.8)	<0.001
Fiji	7.9 (4.51-13.96)	2.24 (1.03-4.47)	8.45 (4.54-14.4)	2.54 (1.16-4.7)	0.49 (0.07 to 0.92)	0.023
Finland	65.74 (42.59-94.88)	5.00 (2.95-7.72)	43.02 (25.86-67.3)	3.40 (1.87-5.52)	-1.10 (-1.37 to -0.83)	<0.001
France	1043.78 (790.48-1340.05)	6.32 (4.18-9.05)	900.24 (648.15-1227.04)	5.57 (3.34-8.2)	-0.47 (-0.9 to -0.04)	0.033
Gabon	46.72 (15.45-97.25)	8.1 (2.27-18.44)	32.09 (12.55-75.45)	4.24 (1.37-11.25)	-2.63 (-3.12 to -2.14)	<0.001
Gambia	47.15 (8.04-125)	7.18 (1.18-20.02)	39.05 (16.99-74.46)	3.27 (1.06-7.21)	-3.08 (-4.03 to -2.13)	<0.001
Georgia	37.93 (24.54-65.69)	2.11 (1.17-3.95)	12.13 (8-20.87)	1.35 (0.8-2.58)	-1.67 (-2.3 to -1.03)	<0.001
Germany	1077.93 (806.99-1446.02)	6.13 (4.07-8.96)	969.05 (731.05-1248.09)	5.93 (3.92-8.47)	0.03 (-0.2 to 0.26)	0.798
Ghana	670.33 (120.4-1545.73)	7.51 (1.2-19.24)	662.36 (254.33-1309.45)	4.53 (1.25-10.72)	-2.01 (-2.59 to -1.43)	<0.001
Greece	66.9 (50.04-88.48)	2.31 (1.47-3.4)	34.91 (24.9-48.04)	1.67 (1.06-2.56)	-0.97 (-1.16 to -0.78)	<0.001
Greenland	1 (0.6-1.94)	5.49 (2.69-11.81)	0.56 (0.35-0.86)	3.64 (1.83-6.6)	-1.35 (-1.8 to -0.9)	<0.001
Grenada	5.63 (3.98-7.96)	14.07 (8.63-21.64)	2.38 (1.74-3.18)	7.81 (4.86-11.84)	-1.89 (-2.14 to -1.65)	<0.001
Guam	1.31 (0.79-2.24)	2.27 (1.1-4.35)	0.79 (0.43-1.44)	1.34 (0.61-2.7)	-1.74 (-2.52 to -0.95)	<0.001
Guatemala	496.28 (206.18-990.26)	9.84 (3.96-19.99)	404.51 (250.87-568.5)	5.26 (2.89-8.2)	-2.74 (-3.51 to -1.96)	<0.001
Guinea	520.8 (43.61-1490.96)	12.41 (1.01-36.8)	522.93 (92.8-1375.97)	6.96 (1.07-19.62)	-2.43 (-3.12 to -1.74)	<0.001
Guinea-Bissau	76.51 (8.46-225.78)	11.76 (1.2-34.89)	50.05 (16.61-102.02)	4.93 (1.28-11.77)	-3.16 (-3.63 to -2.69)	<0.001
Guyana	50.78 (32.8-72.17)	13.5 (7.57-20.97)	24.58 (17.28-34.12)	8.77 (5.4-13.47)	-1.37 (-2.08 to -0.65)	<0.001

Haiti	1572.87 (100.4-5125.94)	41.88 (2.32-140.14)	1042.7 (176.33-2907.87)	18.63 (2.49-54.04)	-3.08 (-3.33 to -2.82)	<0.001
Honduras	727.34 (283-1378.29)	24.87 (8.24-52.73)	358.02 (181.28-778.76)	8.36 (3.05-20.4)	-4.04 (-4.39 to -3.68)	<0.001
Hungary	310.01 (245.48-397.97)	10.59 (7.25-14.75)	168.89 (119.03-228.65)	8.41 (5.21-12.35)	-0.68 (-0.81 to -0.56)	<0.001
Iceland	3.48 (2.62-4.64)	4.08 (2.6-6.07)	2.94 (2.01-4.14)	3.24 (1.99-5)	-0.76 (-0.91 to -0.6)	<0.001
India	26485.37 (4458.08-57102.2)	6.22 (1.07-13.7)	12136.29 (5652.3-18683.81)	2.43 (1.03-4.09)	-3.34 (-3.71 to -2.96)	<0.001
Indonesia	7891.07 (2952.51-15410.81)	9.19 (3.03-18.89)	2740.71 (1934.53-3770.93)	3.24 (2.05-4.85)	-3.56 (-3.83 to -3.29)	<0.001
Iran (Islamic Republic of)	526.88 (260.56-1049.95)	1.65 (0.73-3.44)	411.7 (249.48-561.59)	1.61 (0.91-2.46)	0.03 (-0.19 to 0.25)	0.793
Iraq	1060.41 (365.94-2308)	9.91 (2.86-24.77)	745.43 (347.32-1335.36)	4.16 (1.57-8.85)	-3.18 (-3.77 to -2.59)	<0.001
Ireland	46.62 (36.45-59.4)	3.36 (2.25-5.19)	30.64 (22.01-41.13)	2.21 (1.45-3.31)	-1.29 (-1.94 to -0.64)	<0.001
Israel	81.87 (60.75-110.08)	4.09 (2.54-6.41)	76.66 (59.45-99.51)	2.33 (1.52-3.48)	-1.88 (-2.38 to -1.38)	<0.001
Italy	1493.28 (1034.71-1939.34)	12.16 (6.67-17.31)	572.68 (398.09-734.59)	5.15 (3-6.9)	-2.59 (-2.97 to -2.22)	<0.001
Jamaica	70.03 (37.58-138.99)	6.66 (2.99-14.14)	15.65 (10.62-22.92)	1.90 (1.04-3.39)	-4.67 (-5.59 to -3.74)	<0.001
Japan	842.03 (634.38-1100.51)	2.21 (1.61-3)	889.06 (573.54-1292.93)	3.52 (2.22-5.39)	1.66 (1.59 to 1.74)	<0.001
Jordan	60.44 (38.39-86)	2.87 (1.5-4.98)	116.17 (84.41-156.79)	2.29 (1.39-3.7)	-0.50 (-0.81 to -0.19)	0.001
Kazakhstan	562.71 (332.25-906.59)	8.34 (4.26-14.38)	350.67 (242.94-527.39)	5.43 (3.19-9.16)	-1.44 (-1.68 to -1.21)	<0.001
Kenya	1132.75 (355.22-2411.53)	7.42 (2.25-16.37)	940.45 (566.61-1409.25)	3.81 (2.02-6.17)	-2.63 (-3.08 to -2.17)	<0.001
Kiribati	5.84 (0.84-17.06)	14.57 (1.92-43.68)	3.25 (1.08-6.85)	6.13 (1.6-14.3)	-3.42 (-4.39 to -2.45)	<0.001
Kuwait	16.43 (12.51-21)	2.46 (1.65-3.56)	15.95 (11.98-20.54)	1.49 (1.01-2.12)	-1.64 (-3.03 to -0.22)	0.023
Kyrgyzstan	324.6 (152.44-512.78)	14.57 (6.66-24.66)	95.55 (57.32-138.17)	3.6 (1.94-5.78)	-4.74 (-5.2 to -4.27)	<0.001
Lao People's Democratic Republic	108.38 (18.24-287.62)	4.57 (0.64-13.53)	60.99 (31.86-113.92)	2.05 (0.85-4.73)	-2.79 (-3.25 to -2.32)	<0.001

Latvia	37.41 (25.9-51.91)	4.95 (2.9-7.71)	12.87 (8.38-18.12)	3.27 (1.71-5.27)	-1.63 (-2.33 to -0.91)	<0.001
Lebanon	12.99 (7.59-25.21)	0.87 (0.44-1.81)	15.92 (9.56-25.72)	0.97 (0.51-1.81)	0.38 (0.05 to 0.7)	0.023
Lesotho	66.89 (21.65-145.76)	6.81 (1.67-16.48)	67.71 (27.3-147.08)	8.05 (2.34-19.42)	-0.03 (-1.59 to 1.56)	0.974
Liberia	187.03 (17.98-480.51)	15.67 (1.38-42.53)	111.74 (35.43-289.91)	4.7 (1.25-13.53)	-4.41 (-4.8 to -4.02)	<0.001
Libya	29.52 (13.54-86.77)	1.25 (0.45-3.67)	24.82 (15.62-44.46)	1.18 (0.53-2.56)	-0.16 (-0.41 to 0.09)	0.217
Lithuania	30.81 (23.16-40.35)	2.72 (1.79-4.01)	15.94 (10.79-22.42)	2.78 (1.53-4.39)	0.15 (-0.2 to 0.51)	0.398
Luxembourg	5.71 (4.42-7.11)	6.38 (4.38-8.82)	5.4 (3.8-7.38)	3.94 (2.41-6.13)	-1.60 (-2.14 to -1.06)	<0.001
Madagascar	1040.3 (125.79-2902.46)	13.55 (1.39-39.26)	777.65 (322.94-1623.83)	5.49 (1.76-13)	-3.42 (-3.77 to -3.06)	<0.001
Malawi	1512.04 (128.99-4790.29)	23.4 (1.72-75.24)	817.12 (316.72-1748.95)	8.34 (2.33-19.7)	-4.43 (-5.26 to -3.6)	<0.001
Malaysia	79.96 (45.98-132.9)	0.95 (0.42-1.85)	76.47 (50.93-114.93)	0.71 (0.37-1.31)	-0.99 (-2.11 to 0.14)	0.085
Maldives	6.6 (1.81-18.63)	4.70 (1-14.18)	2.99 (1.54-6.02)	2.07 (0.8-5.01)	-2.82 (-3.68 to -1.95)	<0.001
Mali	820.91 (56.91-2413.83)	13.71 (0.9-41.74)	1140.01 (172.55-3174.96)	8.08 (1.01-24.09)	-2.13 (-3.14 to -1.1)	<0.001
Malta	4.22 (3.18-5.44)	3.73 (2.43-5.38)	2.85 (2.08-3.82)	3.34 (2.12-4.96)	-0.21 (-0.44 to 0.02)	0.077
Marshall Islands	1.57 (0.62-3.22)	5.83 (1.85-12.93)	1 (0.46-1.92)	4.29 (1.53-9.24)	-1.17 (-1.36 to -0.98)	<0.001
Mauritania	77.57 (16.23-175.46)	6.23 (1.2-15.58)	45.8 (21.38-84.82)	2.24 (0.81-5.12)	-3.73 (-4.57 to -2.88)	<0.001
Mauritius	5.38 (3.96-7.55)	1.25 (0.75-2.09)	8.46 (4.87-12.6)	2.14 (1.14-3.33)	3.09 (2.11 to 4.09)	<0.001
Mexico	2115.07 (1626.09-2645.83)	4.87 (3.52-6.36)	1372.51 (1116.01-1641.1)	3.22 (2.36-4.11)	-1.41 (-1.8 to -1.03)	<0.001
Micronesia (Federated States of)	4.7 (1.72-10.73)	7.84 (2.36-18.98)	1.47 (0.59-2.63)	3.6 (0.85-7.66)	-2.83 (-3.14 to -2.53)	<0.001
Monaco	0.19 (0.11-0.32)	3.85 (1.73-7.27)	0.15 (0.1-0.22)	2.12 (1.24-3.65)	-1.76 (-1.91 to -1.61)	<0.001
Mongolia	281.37 (55.54-715.84)	22.89 (3.96-59.53)	56.41 (30.4-113.12)	4.31 (1.9-9.23)	-6.03 (-6.43 to -5.64)	<0.001
Montenegro	18.62 (11.83-29.06)	8.93 (4.63-15.7)	7.28 (4.6-11.04)	4.66 (2.42-8.32)	-1.82 (-2.79 to -0.84)	<0.001

Morocco	148.88 (52.56-530.6)	1.19 (0.36-4.28)	134.4 (76.23-263.35)	1.06 (0.46-2.27)	-0.39 (-0.72 to -0.06)	0.021
Mozambique	1459.83 (147.46-4530.22)	17.36 (1.59-55.95)	1441.91 (535.94-3781.05)	8.05 (2.45-22.07)	-3.19 (-4.45 to -1.92)	<0.001
Myanmar	993.42 (212.37-2672.33)	4.81 (0.8-13.81)	247.15 (132.79-432.54)	1.24 (0.51-2.59)	-4.77 (-5.87 to -3.65)	<0.001
Namibia	72.2 (15.34-207.39)	9.03 (1.47-26.75)	77.59 (30.14-151.12)	7.11 (2.17-15.56)	-1.12 (-2.31 to 0.09)	0.069
Nauru	0.87 (0.18-2.34)	13.43 (2.6-36.68)	0.42 (0.15-0.85)	8.08 (2.4-17.66)	-2.21 (-2.46 to -1.95)	<0.001
Nepal	817.8 (151.42-2224.6)	7.27 (1.2-20.62)	292.09 (165.3-553.47)	2.28 (0.92-5.51)	-4.15 (-4.74 to -3.56)	<0.001
Netherlands	584.5 (422.62-767.32)	15.67 (9.42-22.58)	233.89 (148.93-298.14)	6.44 (3.61-9.05)	-2.99 (-3.43 to -2.55)	<0.001
New Zealand	31.67 (21.69-46.02)	2.6 (1.66-4.03)	28.67 (16.65-44.39)	2.26 (1.22-3.77)	-0.50 (-0.65 to -0.36)	<0.001
Nicaragua	40.14 (22.42-81.15)	1.73 (0.84-3.67)	36.39 (24.5-53.93)	1.4 (0.75-2.48)	-0.89 (-1.33 to -0.46)	<0.001
Niger	926.07 (49.88-2965.34)	15.7 (0.84-51.56)	1253.86 (175.29-3592.7)	7.58 (0.95-22.41)	-2.74 (-3.22 to -2.25)	<0.001
Nigeria	6548.24 (996.53-13727.58)	11.6 (1.65-25.04)	9801.7 (3211.62-18102.84)	8.07 (2.23-15.52)	-1.56 (-2.01 to -1.11)	<0.001
Niue	0.09 (0.03-0.24)	9 (2.45-24.26)	0.03 (0.01-0.08)	6.73 (1.57-17.74)	-1.24 (-1.39 to -1.09)	<0.001
North Macedonia	33.05 (20.96-57.22)	4.71 (2.54-9.05)	15.56 (9.91-22.94)	3.17 (1.73-5.5)	-1.27 (-1.6 to -0.95)	<0.001
Northern Mariana Islands	1.12 (0.55-2.07)	6.19 (2.51-12.59)	0.22 (0.11-0.54)	2.02 (0.69-5.76)	-4.50 (-5.2 to -3.8)	<0.001
Norway	49.57 (36.98-67.32)	4.04 (2.75-6.18)	48.5 (31.91-70.05)	3.55 (2.24-5.32)	-0.58 (-0.91 to -0.25)	0.001
Oman	10.25 (5.07-28.36)	1.11 (0.49-3.02)	13.63 (9.02-22.14)	1.11 (0.59-2.02)	0.28 (0.08 to 0.47)	0.005
Pakistan	3500.87 (646.59-8309.33)	5.16 (0.93-13.42)	4845.75 (1934.21-9293)	4.33 (1.52-9.2)	-0.83 (-1.35 to -0.31)	0.002
Palau	0.48 (0.12-1.32)	7.71 (1.43-22.09)	0.17 (0.08-0.35)	4.2 (1.47-10.05)	-2.34 (-2.46 to -2.22)	<0.001
Palestine	19.84 (6.82-57.34)	1.53 (0.47-4.33)	21.54 (13.44-40.05)	0.92 (0.47-1.83)	-2.10 (-2.89 to -1.31)	<0.001
Panama	223.47 (135.07-335.68)	20.9 (11.65-33.09)	331.47 (127.99-514.4)	22.82 (8.41-37.95)	0.07 (-2.2 to 2.4)	0.951
Papua New Guinea	272.27 (63.17-701.45)	11.66 (2.44-30.9)	474.73 (166.54-1053.9)	9.06 (2.92-20.92)	-0.73 (-1.01 to -0.45)	<0.001

Paraguay	65.94 (43.03-98.23)	3.06 (1.6-5.35)	31.48 (21.85-45.53)	1.18 (0.61-2.12)	-3.23 (-3.65 to -2.82)	<0.001
Peru	6025 (2136.79-11557.88)	54.38 (17.49-108.72)	487.83 (250.25-925.19)	4.12 (1.67-9.13)	-8.89 (-9.49 to -8.29)	<0.001
Philippines	855.61 (305.01-1586.57)	2.58 (0.88-4.84)	734.55 (547.35-955.15)	1.58 (1.02-2.25)	-1.65 (-2.63 to -0.65)	0.001
Poland	857.4 (700.11-1050.28)	7.21 (5.37-9.47)	413.96 (308.36-549.3)	5.27 (3.56-7.27)	-0.84 (-1.04 to -0.64)	<0.001
Portugal	107.3 (80.91-182.73)	3.53 (2.21-7.53)	54.94 (40.73-73.42)	2.39 (1.58-3.74)	-0.95 (-1.23 to -0.67)	<0.001
Puerto Rico	69.49 (42.22-97.46)	5.52 (2.87-8.59)	14.5 (10.59-18.78)	2.01 (1.23-3.03)	-3.18 (-3.62 to -2.74)	<0.001
Qatar	4.69 (2.44-8.2)	3.1 (1.32-6.66)	10.87 (6.41-18.33)	2.16 (1.02-4.42)	-1.38 (-1.94 to -0.82)	<0.001
Republic of Korea	1059.5 (668.07-1427.71)	6.65 (3.57-10.65)	233.12 (161.48-316.25)	2.18 (1.42-3.2)	-3.46 (-3.76 to -3.15)	<0.001
Republic of Moldova	76.45 (49.92-119.67)	4.89 (2.6-8.68)	24.63 (19.05-31.36)	3.22 (1.92-4.81)	-1.64 (-2.08 to -1.19)	<0.001
Romania	1180.18 (748-1816.33)	17.08 (9.94-27.81)	227.87 (145.66-299.1)	5.74 (3.21-8.37)	-3.51 (-4.15 to -2.88)	<0.001
Russian Federation	1572.38 (1290.59-2246.24)	3.51 (2.73-5.34)	712.16 (546.29-1077.1)	2.12 (1.52-3.45)	-1.94 (-2.12 to -1.75)	<0.001
Rwanda	1015.05 (161.92-2833.51)	21.31 (2.91-58.97)	662.05 (255.78-1539.8)	10.75 (3.13-26.49)	-2.78 (-4.35 to -1.17)	0.001
Saint Kitts and Nevis	3.53 (2.3-4.82)	19.92 (11.59-30.5)	1.33 (0.75-2.04)	8.46 (3.65-14.47)	-2.61 (-3.56 to -1.66)	<0.001
Saint Lucia	8.38 (5.57-11.55)	12.31 (7.53-18.2)	2.34 (1.62-3.35)	5.61 (3.35-8.83)	-2.92 (-3.21 to -2.64)	<0.001
Saint Vincent and the Grenadines	8.7 (5.87-12.44)	16.84 (10.08-26.61)	2.67 (1.93-3.62)	7.90 (4.9-12.11)	-2.35 (-2.68 to -2.02)	<0.001
Samoa	5.23 (2.27-10.25)	6.62 (2.25-14.49)	2.22 (1.21-3.92)	2.47 (0.94-5.45)	-2.99 (-3.65 to -2.33)	<0.001
San Marino	0.21 (0.12-0.35)	3.25 (1.38-6.72)	0.17 (0.12-0.25)	2.25 (1.22-3.89)	-1.15 (-1.29 to -1.01)	<0.001
Sao Tome and Principe	5.7 (1.21-14.25)	7.69 (1.23-20.58)	2.31 (1.27-4.55)	2.41 (0.96-5.83)	-4.71 (-7.61 to -1.72)	0.002
Saudi Arabia	105.73 (63.62-175.43)	1.32 (0.63-2.7)	104.66 (58.46-162.6)	0.98 (0.51-1.73)	-0.65 (-1.12 to -0.17)	0.008
Senegal	438.62 (55.53-1089.01)	8.75 (1-23.13)	270.03 (102.31-548.11)	3.47 (1-8.07)	-3.59 (-5.1 to -2.05)	<0.001
Serbia	245.8 (143.71-492.83)	8.99 (4.14-20.5)	99.17 (67.26-139.06)	4.61 (2.72-7.31)	-2.03 (-2.26 to -1.8)	<0.001

Seychelles	0.54<br>(0.23-0.94)	1.71<br>(0.66-3.64)	0.4<br>(0.2-0.76)	1.39<br>(0.6-3.13)	-0.55<br>(-1.01 to -0.09)	0.02
Sierra Leone	292.75<br>(22.94-855.29)	12.78<br>(0.92-38.37)	314.91<br>(64.84-787.9)	7.11<br>(1.17-19.81)	-2.57<br>(-3.35 to -1.77)	<0.001
Singapore	31.25<br>(18.41-42.7)	3.52<br>(1.76-5.38)	11.28<br>(8.11-15.42)	1.07<br>(0.69-1.59)	-4.06<br>(-4.58 to -3.53)	<0.001
Slovakia	197.93<br>(142.57-260.53)	11.66<br>(7.07-17.71)	113.28<br>(68.08-162.58)	10.16<br>(5.5-16.38)	-0.39<br>(-0.67 to -0.11)	0.007
Slovenia	28.97<br>(20.68-40.24)	5<br>(3.19-7.66)	15.64<br>(10.02-22.39)	3.85<br>(2.29-5.86)	-1.03<br>(-1.34 to -0.71)	<0.001
Solomon Islands	10.2<br>(3.22-22.44)	4.78<br>(1.29-11.4)	12.19<br>(5.92-23.86)	3.58<br>(1.4-7.69)	-0.99<br>(-1.97 to 0)	0.05
Somalia	578.34<br>(38.17-1961.21)	12.31<br>(0.75-42.46)	899.71<br>(199.93-2352.11)	6.86<br>(1.26-18.93)	-1.76<br>(-2.4 to -1.12)	<0.001
South Africa	1949.71<br>(638.22-4346.19)	11.45<br>(3.24-26.24)	829.11<br>(540.07-1256.82)	4.33<br>(2.39-7.19)	-3.43<br>(-3.99 to -2.87)	<0.001
South Sudan	719.21<br>(47.47-2395.08)	19.55<br>(1.2-65.91)	617.47<br>(133.33-1554.44)	11.15<br>(1.98-30.08)	-2.22<br>(-2.74 to -1.7)	<0.001
Spain	562.05<br>(417.37-746.63)	4.46<br>(2.98-6.74)	368.33<br>(232.69-521.92)	3.77<br>(2.27-5.81)	-0.72<br>(-0.91 to -0.52)	<0.001
Sri Lanka	193.82<br>(79.76-325.57)	2.72<br>(0.93-5.55)	70.26<br>(39.44-114.1)	1.04<br>(0.44-2.14)	-3.06<br>(-4.72 to -1.38)	<0.001
Sudan	291.24<br>(41.69-1723.13)	2.32<br>(0.36-13.29)	361.23<br>(195.92-832.92)	1.79<br>(0.66-4.55)	-1.12<br>(-1.98 to -0.26)	0.011
Suriname	27.58<br>(12.11-46.82)	16.56<br>(6.4-31.48)	11.42<br>(7.04-18.46)	6.1<br>(2.79-12.07)	-3.49<br>(-4.74 to -2.22)	<0.001
Sweden	137.21<br>(100.72-180.08)	6.16<br>(3.98-8.87)	83.29<br>(57.87-115.86)	3.46<br>(2.13-5.26)	-2.30<br>(-2.87 to -1.72)	<0.001
Switzerland	88.35<br>(63.59-118.08)	5.48<br>(3.36-8.2)	57.64<br>(38.56-84.1)	3.21<br>(1.84-5.15)	-1.83<br>(-2.18 to -1.47)	<0.001
Syrian Arab Republic	196.82<br>(74.28-396.65)	2.52<br>(0.83-6.01)	121.15<br>(77.75-178.07)	2.24<br>(1.02-4.37)	-0.83<br>(-1.31 to -0.36)	0.001
Taiwan (Province of China)	362.91<br>(256.23-467.6)	5.43<br>(3.52-7.74)	81.96<br>(59.28-107.13)	1.95<br>(1.24-2.87)	-3.30<br>(-3.87 to -2.73)	<0.001
Tajikistan	1028.61<br>(238.75-2211.34)	30.18<br>(6.9-66.01)	259.54<br>(143.16-473.82)	6.05<br>(2.77-12.64)	-5.70<br>(-6.12 to -5.29)	<0.001
Thailand	451.16<br>(220.04-749.23)	2.02<br>(0.76-4.36)	119.26<br>(74.67-175.87)	0.8<br>(0.36-1.53)	-2.86<br>(-3.23 to -2.5)	<0.001
Timor-Leste	17.45<br>(2.51-51.79)	3.69<br>(0.49-11.55)	12.29<br>(5.43-21.62)	1.85<br>(0.61-4.02)	-2.97<br>(-3.69 to -2.25)	<0.001
Togo	176.99<br>(27.35-442.18)	7.4<br>(1.01-20.15)	148.79<br>(53.33-300.05)	3.75<br>(1-9)	-2.64<br>(-3.04 to -2.23)	<0.001
Tokelau	0.07<br>(0.02-0.15)	6.49<br>(1.64-15.03)	0.02<br>(0.01-0.03)	2.88<br>(1.2-5.85)	-3.16<br>(-3.96 to -2.35)	<0.001

Tonga	4.59 (2.01-9.8)	9.26 (3.42-21.57)	2.54 (1.26-5.01)	5.58 (2.27-12.27)	-1.70 (-2.29 to -1.11)	<0.001
Trinidad and Tobago	11.37 (8.3-15.47)	2.16 (1.16-3.84)	5.73 (3.98-8.17)	1.55 (0.84-3.1)	-1.38 (-2.41 to -0.34)	0.009
Tunisia	53.49 (25.46-160.29)	1.31 (0.5-4.13)	38.74 (22.43-61.83)	1.12 (0.55-2.1)	-0.56 (-0.82 to -0.29)	<0.001
Turkey	3787.55 (1499.25-7679.1)	13.5 (4.02-33.24)	1506.59 (1006.96-2296.44)	7.09 (3.74-13.01)	-2.10 (-2.49 to -1.71)	<0.001
Turkmenistan	265.11 (76.51-532.74)	12.59 (3.51-25.83)	103.21 (51.2-183.36)	5.27 (2.42-10.08)	-3.3 (-3.59 to -3.01)	<0.001
Tuvalu	0.6 (0.1-1.74)	12.61 (1.81-37.07)	0.14 (0.08-0.25)	3.29 (1.32-6.92)	-5.01 (-5.8 to -4.21)	<0.001
Uganda	1207.66 (166.32-3678.95)	9.52 (1.14-29.39)	1817.85 (687.87-3996.68)	7.2 (2.12-17.47)	-1.35 (-2.44 to -0.25)	0.016
Ukraine	546.53 (361.87-802.1)	3.73 (2.07-6.24)	327.25 (216.66-444.81)	3.77 (2.09-5.86)	0.07 (-0.93 to 1.09)	0.89
United Arab Emirates	5.87 (3.11-10.77)	0.92 (0.45-1.75)	11.87 (7.75-17.24)	0.78 (0.47-1.29)	-0.05 (-0.52 to 0.42)	0.837
United Kingdom	1412.71 (1124.44-1700.15)	9.33 (6.62-12.13)	1080.43 (819.61-1304.31)	6.97 (4.77-8.73)	-1.13 (-1.51 to -0.75)	<0.001
United Republic of Tanzania	2931.53 (460.12-8308.8)	16.81 (2.28-47.83)	4080.64 (1259.12-9222.67)	12.2 (3.13-29.07)	-1.09 (-1.81 to -0.36)	0.003
United States of America	6493.31 (5417.3-7908.5)	8.65 (6.83-11.01)	7139.65 (5737.7-8780.62)	8.46 (6.46-10.66)	-3.37 (-4.01 to -2.74)	<0.001
United States Virgin Islands	2.58 (1.34-4.14)	6.25 (2.65-12.26)	0.6 (0.35-1.03)	2.29 (1.06-4.84)	0.02 (-0.18 to 0.22)	0.86
Uruguay	22.35 (17.52-28.25)	2.02 (1.34-2.97)	21.85 (15.13-29.48)	2.09 (1.17-3.21)	0.30 (-0.44 to 1.05)	0.424
Uzbekistan	1693.79 (758.05-2638.92)	14.3 (6.52-23.64)	1111.69 (473.77-1665.52)	8.38 (3.49-13.73)	-2.17 (-2.4 to -1.95)	<0.001
Vanuatu	4.91 (1.65-10.33)	5.51 (1.49-12.75)	6.03 (2.76-11.85)	4.39 (1.61-9.5)	-1.07 (-2.62 to 0.51)	0.185
Venezuela (Bolivarian Republic of)	283.75 (208.8-383.42)	3.07 (1.97-4.55)	108.36 (76.93-149.87)	1.16 (0.7-1.85)	-2.80 (-3.69 to -1.91)	<0.001
Viet Nam	1173.86 (491.64-2337.56)	3.52 (1.19-7.98)	477.03 (286.02-820.55)	1.72 (0.79-3.62)	-2.41 (-2.58 to -2.25)	<0.001
Yemen	123.03 (30.33-617.64)	1.37 (0.32-6.51)	228.45 (123.87-542.1)	1.42 (0.59-3.55)	0.04 (-0.28 to 0.35)	0.819
Zambia	1261.28 (129.33-3757.87)	23.13 (2.21-69.9)	916.75 (335.89-2012.28)	9.01 (2.8-21.56)	-3.69 (-4.17 to -3.2)	<0.001

Zimbabwe	432.44 (113.7-1156.12)	6.78 (1.45-18.6)	607.47 (194.96-1399.09)	7.85 (1.91-19.51)	0.25 (-1.02 to 1.54)	0.703

Supplementary table 6. Cases and age-standardized for IBD mortality and their average annual percentage changes (AAPCs) from 1990 to 2019 at the global and regional levels.

	1990		2019			
	cases (n)	ASMR (Per 100,000 population, 95% CI)	cases (n)	ASMR (Per 100,000 population, 95% CI)	AAPCs, 1990-2019 (95% CI)	P value
Global	2756.46 (1162.64-4484.91)	0.12 (0.05-0.2)	1208.04 (802.41-1651.44)	0.05 (0.03-0.07)	-3.23 (-3.32 to -3.14)	<0.001
Sex						
Male	1124.1 (433.47-2157.02)	0.09 (0.04-0.18)	569.52 (373.76-797.33)	0.04 (0.03-0.06)	-2.76 (-2.92 to -2.61)	<0.001
Female	1632.36 (527.39-2473.59)	0.14 (0.05-0.22)	638.53 (350.17-1037.79)	0.05 (0.03-0.09)	-3.58 (-3.7 to -3.47)	<0.001
Age group, years						
<5	2094.34 (739.77-3640.22)	0.33 (0.12-0.58)	716.65 (403.38-1055.11)	0.11 (0.06-0.16)	-3.78 (-4.01 to -3.56)	<0.001
5-9	258.06 (139.05-379.58)	0.04 (0.02-0.06)	141.28 (100.96-188.97)	0.02 (0.02-0.03)	-2.47 (-2.7 to -2.25)	<0.001
10-14	153.69 (97.62-200.65)	0.03 (0.02-0.04)	120.25 (91.62-153.37)	0.02 (0.01-0.02)	-1.43 (-1.67 to -1.19)	<0.001
15-19	250.37 (161.98-333.18)	0.05 (0.03-0.06)	229.87 (181.43-291.28)	0.04 (0.03-0.05)	-0.91 (-1.03 to -0.78)	<0.001
Sociodemographic index						
High	124.24 (107.76-138.86)	0.05 (0.05-0.06)	82.5 (66.46-91.80)	0.04 (0.03-0.04)	-1.17 (-1.38 to -0.95)	<0.001

High-middle	398.67 (235.84-563.93)	0.10 (0.06-0.15)	96.91 (77.59-117.44)	0.03 (0.02-0.04)	-4.07 (-4.26 to -3.87)	<0.001
Middle	971.68 (462.23-1424.98)	0.13 (0.06-0.19)	226.32 (183.77-268.87)	0.03 (0.02-0.04)	-4.83 (-5.25 to -4.4)	<0.001
Low-middle	783.67 (218.53-1436.33)	0.13 (0.04-0.24)	320.3 (196.17-460.94)	0.05 (0.03-0.07)	-3.75 (-3.87 to -3.63)	<0.001
Low	476.62 (75.14-1061.84)	0.14 (0.02-0.31)	480.85 (206.17-795.66)	0.08 (0.03-0.13)	-2.38 (-2.58 to -2.18)	<0.001
Region						
Andean Latin America	89.60 (34.5-163.42)	0.45 (0.16-0.85)	12.60 (8.12-18.99)	0.05 (0.03-0.09)	-7.37 (-7.75 to -6.99)	<0.001
Australasia	1.93 (1.41-2.48)	0.03 (0.02-0.04)	1.55 (0.8-2.1)	0.02 (0.01-0.03)	-1.20 (-1.58 to -0.81)	<0.001
Caribbean	26.43 (6.68-69.38)	0.17 (0.04-0.47)	15.83 (4.86-38.19)	0.11 (0.03-0.27)	-1.89 (-2.12 to -1.67)	<0.001
Central Asia	48.4 (28.85-75.35)	0.14 (0.08-0.22)	22.19 (14.31-30.78)	0.06 (0.04-0.09)	-2.72 (-3.01 to -2.42)	<0.001
Central Europe	66.75 (35.49-87.27)	0.19 (0.1-0.26)	10.70 (7.66-13.34)	0.05 (0.03-0.06)	-4.49 (-4.9 to -4.08)	<0.001
Central Latin America	56.09 (43.52-69.41)	0.07 (0.05-0.09)	35.69 (26.12-46.41)	0.04 (0.03-0.06)	-1.59 (-1.84 to -1.34)	<0.001
Central Sub-Saharan Africa	52.64 (11.43-141.88)	0.14 (0.03-0.39)	52.87 (21.03-113.57)	0.07 (0.03-0.16)	-2.74 (-2.87 to -2.6)	<0.001
East Asia	1106.98 (459.45-1710.05)	0.25 (0.1-0.39)	135.26 (95.14-176.28)	0.04 (0.03-0.06)	-5.97 (-6.43 to -5.50)	<0.001
Eastern Europe	24.21 (19.75-34.06)	0.04 (0.03-0.05)	10.58 (8.46-14.84)	0.02 (0.02-0.03)	-1.60 (-1.96 to -1.23)	<0.001
Eastern Sub-Saharan Africa	237.33 (38.36-592.88)	0.19 (0.03-0.47)	215.24 (102.42-378.64)	0.09 (0.04-0.17)	-2.75 (-2.91 to -2.59)	<0.001
High-income Asia Pacific	16.63 (11.42-21.34)	0.03 (0.02-0.05)	1.79 (1.22-3.14)	0.01 (0-0.01)	-6.01 (-6.38 to -5.64)	<0.001

High-income North America	52.33 (46.85-62.02)	0.06 (0.06-0.08)	48.91 (40.93-54.57)	0.06 (0.05-0.07)	-0.56 (-0.96 to -0.15)	0.007
North Africa and Middle East	80.72 (33.46-146.97)	0.04 (0.02-0.09)	48.43 (35.88-66.25)	0.02 (0.01-0.03)	-2.50 (-2.95 to -2.06)	<0.001
Oceania	4.03 (1.24-9.54)	0.11 (0.03-0.27)	6.33 (2.52-13.43)	0.09 (0.03-0.21)	-0.58 (-1.37 to 0.22)	0.154
South Asia	455.98 (78.25-937.99)	0.08 (0.01-0.17)	235.4 (113.01-364.22)	0.03 (0.02-0.06)	-3.14 (-3.41 to -2.88)	<0.001
Southeast Asia	147.89 (58.19-276.53)	0.07 (0.02-0.13)	56.12 (40.5-72.26)	0.03 (0.02-0.04)	-3.32 (-3.56 to -3.08)	<0.001
Southern Latin America	4.14 (3.18-4.91)	0.02 (0.01-0.03)	2.73 (2.24-3.31)	0.01 (0.01-0.02)	-1.40 (-1.64 to -1.16)	<0.001
Southern Sub-Saharan Africa	30.92 (10.54-64.86)	0.12 (0.04-0.25)	20.29 (11.76-34.21)	0.07 (0.03-0.12)	-1.96 (-2.52 to -1.4)	<0.001
Tropical Latin America	36.89 (29.78-47.17)	0.06 (0.04-0.07)	23.21 (19.33-27.74)	0.04 (0.03-0.04)	-1.45 (-1.66 to -1.24)	<0.001
Western Europe	59.34 (48.85-70.44)	0.06 (0.05-0.08)	32.12 (23.11-36.73)	0.04 (0.02-0.04)	-1.98 (-2.21 to -1.75)	<0.001
Western Sub-Saharan Africa	157.22 (23.07-322.37)	0.13 (0.02-0.27)	220.21 (71.17-380.38)	0.09 (0.03-0.15)	-1.71 (-2.05 to -1.38)	<0.001

Supplementary table 7. Cases and age-standardized for IBD mortality and their average annual percentage changes (AAPCs) from 1990 to 2019 at the national levels.

	1990		2019			
	Cases (n)	ASMR (per 100,000 population, 95% CI)	Cases (n)	ASMR (per 100,000 population, 95% CI)	AAPCs, 1990-2019 (95% CI)	P value
Afghanistan	1.88 (0.21-9.35)	0.03 (0-0.14)	4.21 (1.32-11.68)	0.02 (0-0.06)	-1.15 (-2.16 to -0.13)	0.027

Albania	35.42 (5.28-50.7)	2.41 (0.34-3.55)	2.61 (1.26-4.71)	0.43 (0.18-0.81)	-5.78 (-6.93 to -4.6)	<0.001
Algeria	1.74 (0.5-6.8)	0.01 (0-0.05)	1.52 (0.76-3.15)	0.01 (0-0.02)	-0.73 (-0.93 to -0.53)	<0.001
American Samoa	0.03 (0.01-0.05)	0.1 (0.04-0.2)	0.01 (0-0.02)	0.04 (0.02-0.08)	-3.53 (-4.02 to -3.04)	<0.001
Andorra	0.01 (0-0.01)	0.06 (0.02-0.14)	0.00 (0-0.01)	0.03 (0.01-0.05)	-3.04 (-3.63 to -2.45)	<0.001
Angola	11.54 (1.4-39.6)	0.17 (0.02-0.58)	15.77 (6.19-33.29)	0.09 (0.03-0.2)	-2.67 (-2.89 to -2.45)	<0.001
Antigua and Barbuda	0.02 (0.01-0.03)	0.09 (0.05-0.15)	0.02 (0.01-0.02)	0.07 (0.04-0.12)	-1.07 (-1.65 to -0.48)	<0.001
Argentina	2.31 (1.79-2.87)	0.02 (0.01-0.03)	1.84 (1.41-2.36)	0.01 (0.01-0.02)	-0.97 (-1.38 to -0.57)	<0.001
Armenia	0.15 (0.07-0.46)	0.01 (0-0.03)	0.08 (0.03-0.27)	0.01 (0-0.04)	-0.23 (-0.85 to 0.39)	0.462
Australia	1.79 (1.26-2.34)	0.04 (0.02-0.05)	1.51 (0.71-2.06)	0.03 (0.01-0.04)	-1.10 (-1.45 to -0.76)	<0.001
Austria	2.78 (1.68-3.79)	0.16 (0.09-0.23)	0.77 (0.55-1)	0.04 (0.03-0.07)	-4.21 (-4.7 to -3.72)	<0.001
Azerbaijan	0.51 (0.26-1.26)	0.02 (0.01-0.04)	0.30 (0.16-0.56)	0.01 (0-0.02)	-1.68 (-2.23 to -1.13)	<0.001
Bahamas	0.08 (0.05-0.11)	0.08 (0.04-0.13)	0.04 (0.03-0.06)	0.04 (0.02-0.07)	-2.08 (-3.04 to -1.11)	<0.001
Bahrain	0.03 (0.02-0.05)	0.02 (0.01-0.03)	0.05 (0.02-0.08)	0.01 (0.01-0.03)	-0.55 (-1.29 to 0.19)	0.147
Bangladesh	90.7 (15.47-210.63)	0.14 (0.02-0.37)	31.83 (13.64-55.74)	0.05 (0.02-0.13)	-3.50 (-3.82 to -3.18)	<0.001
Barbados	0.04 (0.03-0.06)	0.05 (0.03-0.08)	0.02 (0.01-0.02)	0.02 (0.01-0.04)	-2.64 (-3.26 to -2.03)	<0.001
Belarus	0.9 (0.58-1.4)	0.03 (0.02-0.05)	0.41 (0.23-0.67)	0.02 (0.01-0.04)	-1.36 (-1.73 to -0.99)	<0.001
Belgium	0.93 (0.71-1.22)	0.04 (0.03-0.06)	0.54 (0.39-0.74)	0.02 (0.01-0.03)	-2.01 (-2.86 to -1.14)	<0.001
Belize	0.21 (0.13-0.33)	0.20 (0.11-0.34)	0.10 (0.07-0.14)	0.06 (0.04-0.1)	-4.29 (-4.57 to -4.01)	<0.001
Benin	3.99 (0.43-10.83)	0.12 (0.01-0.33)	5.68 (1.44-13.6)	0.07 (0.02-0.19)	-1.88 (-2.22 to -1.53)	<0.001
Bermuda	0.02 (0.01-0.02)	0.10 (0.05-0.16)	0 (0-0.01)	0.04 (0.02-0.06)	-3.76 (-4.16 to -3.36)	<0.001
Bhutan	0.27 (0.03-0.78)	0.08 (0.01-0.26)	0.10 (0.04-0.22)	0.04 (0.01-0.1)	-2.96 (-3.3 to -2.63)	<0.001
Bolivia (Plurinational State of)	13.06 (2.65-33.41)	0.34 (0.06-0.91)	4.37 (2.02-7.89)	0.08 (0.03-0.17)	-4.96 (-5.25 to -4.67)	<0.001

Bosnia and Herzegovina	0.42 (0.19-1.24)	0.03 (0.01-0.1)	0.15 (0.08-0.28)	0.02 (0.01-0.05)	-1.01 (-1.87 to -0.14)	0.023
Botswana	0.74 (0.26-1.63)	0.10 (0.03-0.25)	1.27 (0.29-3.46)	0.14 (0.02-0.41)	1.11 (-0.8 to 3.05)	0.258
Brazil	36.19 (29.22-46.38)	0.06 (0.04-0.07)	22.93 (19.07-27.34)	0.04 (0.03-0.05)	-1.39 (-1.61 to -1.18)	<0.001
Brunei Darussalam	0.07 (0.04-0.11)	0.06 (0.03-0.12)	0.04 (0.03-0.06)	0.03 (0.02-0.06)	-2.04 (-2.36 to -1.73)	<0.001
Bulgaria	3.09 (1.41-4.61)	0.15 (0.06-0.24)	0.96 (0.38-1.42)	0.08 (0.03-0.13)	-2.40 (-3.41 to -1.38)	<0.001
Burkina Faso	10.69 (0.97-29.47)	0.16 (0.01-0.46)	18.73 (3.06-46.02)	0.13 (0.02-0.35)	-0.75 (-1.33 to -0.16)	0.012
Burundi	7.14 (0.88-20.99)	0.19 (0.02-0.57)	6.31 (2.39-15.07)	0.09 (0.03-0.22)	-2.99 (-4.14 to -1.83)	<0.001
Cabo Verde	0.07 (0.02-0.15)	0.04 (0.01-0.09)	0.04 (0.02-0.07)	0.02 (0.01-0.04)	-2.22 (-3.26 to -1.18)	<0.001
Cambodia	6.49 (1.38-16.85)	0.1 (0.02-0.28)	2.65 (1.32-4.92)	0.04 (0.02-0.09)	-3.35 (-3.66 to -3.04)	<0.001
Cameroon	4.99 (0.84-11.97)	0.08 (0.01-0.2)	8.62 (2.56-19.17)	0.06 (0.01-0.14)	-1.28 (-1.57 to -0.99)	<0.001
Canada	4.28 (3.4-5.39)	0.06 (0.04-0.08)	2.06 (1.46-3.12)	0.03 (0.02-0.04)	-2.60 (-3 to -2.19)	<0.001
Central African Republic	2.2 (0.59-5.72)	0.13 (0.03-0.36)	3.02 (0.9-7.67)	0.10 (0.02-0.28)	-1.04 (-1.3 to -0.77)	<0.001
Chad	4.66 (0.45-12.52)	0.11 (0.01-0.31)	9.24 (1.67-23.63)	0.08 (0.01-0.23)	-1.20 (-1.52 to -0.88)	<0.001
Chile	1.58 (1.03-2.03)	0.03 (0.02-0.04)	0.62 (0.45-0.86)	0.01 (0.01-0.02)	-2.92 (-3.48 to -2.37)	<0.001
China	1084.14 (448.44-1668.65)	0.25 (0.1-0.4)	130.82 (91.53-171.05)	0.04 (0.03-0.06)	-6.01 (-6.49 to -5.53)	<0.001
Colombia	8.19 (5.31-11.06)	0.05 (0.03-0.08)	5.47 (3.63-7.83)	0.04 (0.02-0.06)	-1.30 (-1.6 to -0.99)	<0.001
Comoros	0.32 (0.04-0.85)	0.11 (0.01-0.31)	0.22 (0.1-0.43)	0.07 (0.02-0.17)	-1.66 (-2.1 to -1.22)	<0.001
Congo	1.61 (0.55-3.65)	0.11 (0.03-0.26)	1.38 (0.6-3.04)	0.05 (0.02-0.14)	-2.51 (-2.81 to -2.22)	<0.001
Cook Islands	0.01 (0.01-0.02)	0.15 (0.05-0.32)	0 (0-0)	0.04 (0.01-0.1)	-4.32 (-4.63 to -4.01)	<0.001
Costa Rica	0.11 (0.07-0.21)	0.01 (0-0.02)	0.09 (0.05-0.18)	0.01 (0-0.01)	-0.7 (-1.82 to 0.43)	0.222
Croatia	0.39 (0.29-0.53)	0.03 (0.02-0.05)	0.18 (0.11-0.26)	0.02 (0.01-0.04)	-0.98 (-1.62 to -0.33)	0.003
Cuba	0.49 (0.3-0.83)	0.01 (0.01-0.02)	0.12 (0.07-0.26)	0 (0-0.01)	-3.49 (-4.1 to -2.88)	<0.001

Cyprus	0.19<br>(0.1-0.32)	0.08<br>(0.03-0.15)	0.07<br>(0.04-0.12)	0.03<br>(0.01-0.05)	-3.57<br>(-3.83 to -3.3)	<0.001
Czechia	1.13<br>(0.85-1.74)	0.04<br>(0.02-0.06)	0.42<br>(0.28-0.6)	0.02<br>(0.01-0.03)	-2.47<br>(-2.99 to -1.93)	<0.001
C 么 te d'Ivoire	5.7<br>(0.88-14.23)	0.07<br>(0.01-0.2)	7.48<br>(2.24-16.03)	0.05<br>(0.01-0.13)	-1.25<br>(-1.9 to -0.59)	<0.001
Democratic People's Republic of Korea	18.6<br>(4.67-46.2)	0.19<br>(0.04-0.48)	3.69<br>(1.78-7.69)	0.06<br>(0.02-0.14)	-4.70<br>(-5.06 to -4.34)	<0.001
Democratic Republic of the Congo	36.42<br>(7.06-104.3)	0.14<br>(0.02-0.41)	32.05<br>(11.65-78.17)	0.07<br>(0.02-0.18)	-3.02<br>(-3.28 to -2.77)	<0.001
Denmark	0.28<br>(0.2-0.4)	0.02<br>(0.01-0.04)	0.16<br>(0.1-0.24)	0.01<br>(0.01-0.02)	-2.20<br>(-2.92 to -1.48)	<0.001
Djibouti	0.35<br>(0.06-1.04)	0.11<br>(0.02-0.33)	0.49<br>(0.14-1.1)	0.09<br>(0.02-0.21)	-1.16<br>(-1.85 to -0.46)	0.001
Dominica	0.03<br>(0.02-0.05)	0.1<br>(0.05-0.19)	0.01<br>(0.01-0.02)	0.08<br>(0.03-0.15)	-0.98<br>(-1.74 to -0.21)	0.013
Dominican Republic	3.28<br>(1.3-6.83)	0.09<br>(0.03-0.19)	1.80<br>(0.84-3.63)	0.04<br>(0.02-0.1)	-2.57<br>(-3.64 to -1.49)	<0.001
Ecuador	5.85<br>(3.31-8.88)	0.11<br>(0.06-0.19)	2.47<br>(1.37-3.8)	0.04<br>(0.02-0.07)	-3.73<br>(-4.28 to -3.18)	<0.001
Egypt	4.02<br>(1.25-8.28)	0.01<br>(0-0.03)	3.75<br>(1.81-7.17)	0.01<br>(0-0.02)	-1.60<br>(-1.98 to -1.22)	<0.001
El Salvador	3.11<br>(1.46-5.37)	0.11<br>(0.05-0.21)	0.22<br>(0.12-0.4)	0.01<br>(0-0.02)	-8.22<br>(-8.65 to -7.79)	<0.001
Equatorial Guinea	0.31<br>(0.07-0.89)	0.11<br>(0.02-0.3)	0.28<br>(0.12-0.59)	0.04<br>(0.01-0.1)	-4.24<br>(-5 to -3.48)	<0.001
Eritrea	3.15<br>(0.34-10.04)	0.16<br>(0.02-0.51)	3.93<br>(1.41-9.23)	0.11<br>(0.03-0.29)	-1.50<br>(-1.94 to -1.05)	<0.001
Estonia	0.58<br>(0.3-0.85)	0.13<br>(0.06-0.2)	0.08<br>(0.04-0.12)	0.03<br>(0.01-0.05)	-4.80<br>(-5.01 to -4.59)	<0.001
Eswatini	0.65<br>(0.12-1.81)	0.12<br>(0.02-0.36)	0.53<br>(0.25-1.02)	0.1<br>(0.03-0.22)	-1.25<br>(-1.99 to -0.5)	0.001
Ethiopia	75.29<br>(10.38-213.5)	0.22<br>(0.03-0.62)	50.28<br>(22.6-100.1)	0.08<br>(0.03-0.17)	-3.61<br>(-4.43 to -2.78)	<0.001
Fiji	0.09<br>(0.05-0.17)	0.03<br>(0.01-0.05)	0.10<br>(0.05-0.17)	0.03<br>(0.01-0.06)	0.47<br>(0.04 to 0.9)	0.033
Finland	0.16<br>(0.11-0.23)	0.01<br>(0.01-0.02)	0.08<br>(0.05-0.11)	0.01<br>(0-0.01)	-2.21<br>(-3.24 to -1.16)	<0.001
France	6.76<br>(5.15-8.89)	0.04<br>(0.03-0.06)	5.03<br>(3.23-6.7)	0.03<br>(0.02-0.05)	-1.07<br>(-1.91 to -0.22)	0.014
Gabon	0.55<br>(0.18-1.15)	0.10<br>(0.03-0.22)	0.38<br>(0.14-0.89)	0.05<br>(0.02-0.13)	-2.64<br>(-3.13 to -2.14)	<0.001
Gambia	0.56<br>(0.1-1.48)	0.09<br>(0.01-0.24)	0.47<br>(0.2-0.9)	0.04<br>(0.01-0.09)	-3.03<br>(-3.99 to -2.07)	<0.001

Georgia	0.3<br>(0.15-0.63)	0.02<br>(0.01-0.04)	0.07<br>(0.04-0.18)	0.01<br>(0-0.02)	-2.55<br>(-3.94 to -1.14)	<0.001
Germany	9.76<br>(7.02-14.2)	0.06<br>(0.04-0.09)	7.66<br>(5.69-9.7)	0.05<br>(0.03-0.07)	-0.49<br>(-0.88 to -0.1)	0.014
Ghana	8.01<br>(1.43-18.19)	0.09<br>(0.01-0.23)	8.02<br>(3.03-15.69)	0.05<br>(0.01-0.13)	-1.98<br>(-2.53 to -1.42)	<0.001
Greece	0.39<br>(0.28-0.51)	0.02<br>(0.01-0.02)	0.14<br>(0.1-0.2)	0.01<br>(0-0.01)	-2.36<br>(-3.12 to -1.59)	<0.001
Greenland	0.01<br>(0-0.02)	0.04<br>(0.02-0.11)	0<br>(0-0.01)	0.02<br>(0.01-0.04)	-2.93<br>(-3.68 to -2.18)	<0.001
Grenada	0.07<br>(0.05-0.1)	0.17<br>(0.1-0.26)	0.03<br>(0.02-0.04)	0.09<br>(0.06-0.14)	-1.82<br>(-2.08 to -1.55)	<0.001
Guam	0.02<br>(0.01-0.03)	0.03<br>(0.01-0.05)	0.01<br>(0-0.02)	0.01<br>(0.01-0.03)	-1.88<br>(-2.71 to -1.04)	<0.001
Guatemala	5.81<br>(2.41-11.6)	0.12<br>(0.05-0.24)	4.84<br>(2.85-6.85)	0.06<br>(0.03-0.1)	-2.66<br>(-3.48 to -1.84)	<0.001
Guinea	6.12<br>(0.51-17.41)	0.15<br>(0.01-0.44)	6.21<br>(1.08-16.17)	0.08<br>(0.01-0.23)	-2.40<br>(-3.09 to -1.71)	<0.001
Guinea-Bissau	0.91<br>(0.1-2.67)	0.14<br>(0.01-0.42)	0.61<br>(0.2-1.22)	0.06<br>(0.02-0.14)	-3.11<br>(-3.57 to -2.65)	<0.001
Guyana	0.61<br>(0.39-0.86)	0.16<br>(0.09-0.25)	0.3<br>(0.21-0.42)	0.11<br>(0.06-0.16)	-1.32<br>(-2.14 to -0.49)	0.002
Haiti	18.43<br>(1.2-59.77)	0.49<br>(0.03-1.64)	12.28<br>(2.06-33.97)	0.22<br>(0.03-0.64)	-3.06<br>(-3.31 to -2.8)	<0.001
Honduras	8.62<br>(3.39-16.16)	0.3<br>(0.1-0.63)	4.24<br>(2.13-9.12)	0.10<br>(0.03-0.24)	-4.04<br>(-4.39 to -3.68)	<0.001
Hungary	2.31<br>(1.8-2.91)	0.09<br>(0.06-0.12)	0.75<br>(0.49-1.02)	0.04<br>(0.02-0.06)	-2.36<br>(-2.8 to -1.92)	<0.001
Iceland	0.03<br>(0.02-0.03)	0.03<br>(0.02-0.05)	0.01<br>(0.01-0.02)	0.02<br>(0.01-0.03)	-2.13<br>(-2.39 to -1.88)	<0.001
India	313.8<br>(49.18-674.98)	0.07<br>(0.01-0.16)	141.65<br>(61.25-224.94)	0.03<br>(0.01-0.05)	-3.37<br>(-3.76 to -2.99)	<0.001
Indonesia	95.37<br>(36.26-184.25)	0.11<br>(0.04-0.23)	33.38<br>(23.4-46.09)	0.04<br>(0.02-0.06)	-3.53<br>(-3.79 to -3.28)	<0.001
Iran (Islamic Republic of)	5.44<br>(2.3-11.65)	0.02<br>(0.01-0.04)	4.28<br>(2.31-6.06)	0.02<br>(0.01-0.03)	0.04<br>(-0.2 to 0.29)	0.731
Iraq	12.56<br>(4.32-27.31)	0.12<br>(0.03-0.3)	8.40<br>(3.7-15.18)	0.05<br>(0.02-0.1)	-3.35<br>(-3.97 to -2.73)	<0.001
Ireland	0.35<br>(0.27-0.46)	0.03<br>(0.02-0.04)	0.15<br>(0.11-0.23)	0.01<br>(0.01-0.02)	-2.40<br>(-4.12 to -0.65)	0.007
Israel	0.77<br>(0.53-1.09)	0.04<br>(0.02-0.07)	0.61<br>(0.44-0.83)	0.02<br>(0.01-0.03)	-2.42<br>(-3.11 to -1.72)	<0.001
Italy	12.51<br>(7.68-17.17)	0.11<br>(0.06-0.16)	3.43<br>(1.6-4.1)	0.03<br>(0.02-0.04)	-3.76<br>(-4.3 to -3.21)	<0.001

Jamaica	0.8 (0.42-1.6)	0.08 (0.03-0.16)	0.16 (0.1-0.24)	0.02 (0.01-0.04)	-5.18 (-6.27 to -4.07)	<0.001
Japan	4.41 (3.86-5.18)	0.01 (0.01-0.02)	1.05 (0.72-2.01)	0 (0-0.01)	-3.41 (-3.74 to -3.07)	<0.001
Jordan	0.58 (0.31-0.89)	0.03 (0.01-0.05)	0.83 (0.54-1.2)	0.02 (0.01-0.03)	-1.55 (-1.88 to -1.22)	<0.001
Kazakhstan	6.21 (3.46-10.29)	0.09 (0.04-0.16)	3.76 (2.52-5.96)	0.06 (0.03-0.1)	-1.45 (-1.7 to -1.2)	<0.001
Kenya	13.37 (4.26-28.26)	0.09 (0.03-0.19)	11.40 (6.88-17.1)	0.05 (0.02-0.08)	-2.53 (-2.98 to -2.08)	<0.001
Kiribati	0.07 (0.01-0.2)	0.18 (0.02-0.52)	0.04 (0.01-0.08)	0.07 (0.02-0.17)	-3.37 (-4.32 to -2.42)	<0.001
Kuwait	0.14 (0.1-0.19)	0.02 (0.01-0.03)	0.13 (0.1-0.17)	0.01 (0.01-0.02)	-1.32 (-4.04 to 1.48)	0.351
Kyrgyzstan	3.74 (1.65-5.98)	0.17 (0.07-0.29)	0.97 (0.52-1.46)	0.04 (0.02-0.06)	-5.14 (-5.65 to -4.63)	<0.001
Lao People's Democratic Republic	1.30 (0.21-3.43)	0.06 (0.01-0.16)	0.73 (0.37-1.35)	0.02 (0.01-0.06)	-2.79 (-3.26 to -2.33)	<0.001
Latvia	0.35 (0.22-0.52)	0.05 (0.02-0.08)	0.09 (0.05-0.13)	0.02 (0.01-0.04)	-2.66 (-3.62 to -1.69)	<0.001
Lebanon	0.10 (0.05-0.25)	0.01 (0-0.02)	0.12 (0.06-0.24)	0.01 (0-0.02)	0.55 (-0.45 to 1.56)	0.28
Lesotho	0.79 (0.26-1.73)	0.08 (0.02-0.2)	0.81 (0.33-1.74)	0.10 (0.03-0.23)	0.02 (-0.7 to 0.74)	0.965
Liberia	2.20 (0.21-5.62)	0.18 (0.02-0.5)	1.35 (0.43-3.47)	0.06 (0.01-0.16)	-4.33 (-4.71 to -3.95)	<0.001
Libya	0.3 (0.11-0.99)	0.01 (0-0.04)	0.22 (0.11-0.47)	0.01 (0-0.03)	-0.63 (-1.07 to -0.19)	0.005
Lithuania	0.2 (0.14-0.28)	0.02 (0.01-0.03)	0.07 (0.04-0.11)	0.01 (0.01-0.02)	-1.12 (-2.48 to 0.26)	0.11
Luxembourg	0.05 (0.04-0.06)	0.06 (0.04-0.08)	0.04 (0.02-0.05)	0.03 (0.02-0.04)	-2.22 (-3.15 to -1.28)	<0.001
Madagascar	12.30 (1.49-34.17)	0.16 (0.02-0.47)	9.31 (3.91-19.42)	0.07 (0.02-0.16)	-3.37 (-3.76 to -2.97)	<0.001
Malawi	17.72 (1.54-55.8)	0.28 (0.02-0.88)	9.73 (3.77-20.5)	0.1 (0.03-0.23)	-4.38 (-5.19 to -3.55)	<0.001
Malaysia	0.88 (0.48-1.49)	0.01 (0-0.02)	0.73 (0.44-1.2)	0.01 (0-0.01)	-1.3 (-2.03 to -0.56)	0.001
Maldives	0.08 (0.02-0.22)	0.06 (0.01-0.17)	0.03 (0.02-0.07)	0.02 (0.01-0.06)	-2.88 (-3.76 to -1.99)	<0.001
Mali	9.65 (0.68-28.12)	0.16 (0.01-0.49)	13.52 (2-37.3)	0.1 (0.01-0.29)	-2.10 (-3.09 to -1.11)	<0.001
Malta	0.04 (0.03-0.05)	0.03 (0.02-0.05)	0.02 (0.02-0.03)	0.03 (0.02-0.04)	-0.41 (-0.65 to -0.17)	0.001

Marshall Islands	0.02 (0.01-0.04)	0.07 (0.02-0.16)	0.01 (0.01-0.02)	0.05 (0.02-0.11)	-1.15 (-1.34 to -0.96)	<0.001
Mauritania	0.93 (0.19-2.08)	0.08 (0.01-0.19)	0.55 (0.24-1.03)	0.03 (0.01-0.06)	-3.74 (-4.56 to -2.91)	<0.001
Mauritius	0.06 (0.05-0.09)	0.01 (0.01-0.03)	0.11 (0.06-0.17)	0.03 (0.01-0.04)	3.55 (2.4 to 4.72)	<0.001
Mexico	24.06 (18.35-30.25)	0.06 (0.04-0.07)	15.49 (12.31-18.56)	0.04 (0.03-0.05)	-1.44 (-1.86 to -1.01)	<0.001
Micronesia (Federated States of)	0.06 (0.02-0.13)	0.09 (0.03-0.23)	0.02 (0.01-0.03)	0.04 (0.01-0.09)	-2.78 (-3.07 to -2.48)	<0.001
Monaco	0 (0-0)	0.03 (0.01-0.07)	0 (0-0)	0.01 (0.01-0.03)	-2.95 (-3.37 to -2.52)	<0.001
Mongolia	3.24 (0.59-8.31)	0.26 (0.04-0.69)	0.6 (0.29-1.26)	0.04 (0.02-0.1)	-6.27 (-6.69 to -5.85)	<0.001
Montenegro	0.17 (0.1-0.28)	0.08 (0.04-0.16)	0.05 (0.02-0.09)	0.03 (0.01-0.07)	-2.77 (-4.12 to -1.41)	<0.001
Morocco	1.50 (0.36-6.05)	0.01 (0-0.05)	1.28 (0.58-2.84)	0.01 (0-0.03)	-0.58 (-1.01 to -0.14)	0.01
Mozambique	17.11 (1.74-52.85)	0.20 (0.02-0.66)	17.22 (6.46-44.5)	0.10 (0.03-0.26)	-3.12 (-4.36 to -1.86)	<0.001
Myanmar	11.84 (2.46-31.54)	0.06 (0.01-0.17)	2.85 (1.48-5.07)	0.01 (0.01-0.03)	-4.87 (-5.98 to -3.74)	<0.001
Namibia	0.85 (0.18-2.42)	0.11 (0.02-0.31)	0.91 (0.36-1.79)	0.08 (0.02-0.18)	-1.10 (-2.28 to 0.1)	0.072
Nauru	0.01 (0-0.03)	0.16 (0.03-0.43)	0 (0-0.01)	0.10 (0.03-0.21)	-2.18 (-2.43 to -1.92)	<0.001
Nepal	9.75 (1.73-26.12)	0.09 (0.01-0.25)	3.44 (1.85-6.7)	0.03 (0.01-0.07)	-4.21 (-4.8 to -3.62)	<0.001
Netherlands	5.65 (3.82-7.46)	0.16 (0.09-0.23)	2.49 (1.5-3.22)	0.07 (0.04-0.1)	-2.70 (-3.4 to -1.99)	<0.001
New Zealand	0.14 (0.11-0.19)	0.01 (0.01-0.02)	0.04 (0.03-0.09)	0 (0-0.01)	-4.22 (-4.9 to -3.52)	<0.001
Nicaragua	0.43 (0.23-0.92)	0.02 (0.01-0.04)	0.37 (0.24-0.59)	0.01 (0.01-0.03)	-1.10 (-1.68 to -0.52)	<0.001
Niger	10.87 (0.58-34.61)	0.19 (0.01-0.61)	14.84 (2.1-42.39)	0.09 (0.01-0.27)	-2.71 (-3.19 to -2.23)	<0.001
Nigeria	77.09 (11.88-160.52)	0.14 (0.02-0.3)	116.02 (38.55-214.25)	0.10 (0.03-0.18)	-1.54 (-1.99 to -1.09)	<0.001
Niue	0 (0-0)	0.11 (0.03-0.29)	0 (0-0)	0.08 (0.02-0.21)	-1.22 (-1.37 to -1.07)	<0.001
North Macedonia	0.23 (0.12-0.52)	0.03 (0.02-0.08)	0.07 (0.04-0.13)	0.02 (0.01-0.04)	-2.86 (-3.79 to -1.92)	<0.001
Northern Mariana Islands	0.01 (0.01-0.02)	0.07 (0.03-0.15)	0 (0-0.01)	0.02 (0.01-0.07)	-4.62 (-5.36 to -3.87)	<0.001

Norway	0.26 (0.21-0.43)	0.02 (0.02-0.04)	0.1 (0.07-0.17)	0.01 (0.01-0.01)	-3.82 (-4.36 to -3.28)	<0.001
Oman	0.09 (0.03-0.32)	0.01 (0-0.03)	0.12 (0.06-0.22)	0.01 (0-0.02)	0.20 (-0.1 to 0.51)	0.191
Pakistan	41.46 (7.14-97.75)	0.06 (0.01-0.16)	58.38 (23.26-111.27)	0.05 (0.02-0.11)	-0.81 (-1.37 to -0.24)	0.005
Palau	0.01 (0-0.02)	0.09 (0.02-0.26)	0 (0-0)	0.05 (0.02-0.12)	-2.32 (-2.45 to -2.19)	<0.001
Palestine	0.2 (0.04-0.64)	0.01 (0-0.05)	0.16 (0.08-0.39)	0.01 (0-0.02)	-3.23 (-4.29 to -2.16)	<0.001
Panama	2.61 (1.58-3.93)	0.24 (0.14-0.39)	3.92 (1.52-6.07)	0.27 (0.1-0.45)	0.11 (-2.23 to 2.51)	0.925
Papua New Guinea	3.19 (0.75-8.19)	0.14 (0.03-0.36)	5.56 (1.99-12.33)	0.11 (0.03-0.25)	-0.73 (-1 to -0.45)	<0.001
Paraguay	0.7 (0.43-1.08)	0.03 (0.02-0.06)	0.28 (0.18-0.44)	0.01 (0-0.02)	-3.92 (-4.35 to -3.48)	<0.001
Peru	70.69 (25.29-135.06)	0.64 (0.21-1.28)	5.76 (2.89-11.02)	0.05 (0.02-0.11)	-8.88 (-9.49 to -8.26)	<0.001
Philippines	10.12 (3.59-18.68)	0.03 (0.01-0.06)	8.49 (6.4-11)	0.02 (0.01-0.03)	-1.73 (-2.73 to -0.72)	0.001
Poland	6.61 (5.57-8.18)	0.06 (0.05-0.08)	1.91 (1.37-2.34)	0.03 (0.02-0.03)	-2.48 (-2.89 to -2.07)	<0.001
Portugal	1.05 (0.75-1.98)	0.04 (0.02-0.08)	0.4 (0.28-0.56)	0.02 (0.01-0.03)	-2.03 (-2.29 to -1.78)	<0.001
Puerto Rico	0.75 (0.44-1.08)	0.06 (0.03-0.1)	0.13 (0.09-0.18)	0.02 (0.01-0.03)	-3.95 (-4.74 to -3.15)	<0.001
Qatar	0.05 (0.02-0.1)	0.03 (0.01-0.08)	0.11 (0.05-0.2)	0.02 (0.01-0.05)	-1.68 (-2.35 to -1)	<0.001
Republic of Korea	11.86 (7.03-16.55)	0.08 (0.04-0.12)	0.64 (0.37-1.2)	0.01 (0-0.01)	-7.94 (-8.26 to -7.61)	<0.001
Republic of Moldova	0.82 (0.5-1.31)	0.05 (0.02-0.1)	0.22 (0.16-0.29)	0.03 (0.01-0.05)	-2.10 (-3.21 to -0.97)	<0.001
Romania	12.74 (7.71-20.34)	0.19 (0.11-0.31)	1.97 (1.01-2.69)	0.05 (0.02-0.08)	-4.15 (-4.99 to -3.3)	<0.001
Russian Federation	15.68 (13.01-23.76)	0.04 (0.03-0.05)	6.2 (4.69-10.76)	0.02 (0.01-0.03)	-2.14 (-3.01 to -1.25)	<0.001
Rwanda	11.98 (1.96-33.32)	0.25 (0.04-0.7)	7.86 (3.05-18.09)	0.13 (0.04-0.31)	-2.76 (-4.35 to -1.15)	0.001
Saint Kitts and Nevis	0.04 (0.03-0.06)	0.24 (0.14-0.37)	0.02 (0.01-0.02)	0.1 (0.04-0.18)	-2.49 (-3.49 to -1.48)	<0.001
Saint Lucia	0.1 (0.07-0.14)	0.15 (0.09-0.22)	0.03 (0.02-0.04)	0.07 (0.04-0.1)	-2.96 (-3.27 to -2.64)	<0.001
Saint Vincent and the Grenadines	0.1 (0.07-0.15)	0.2 (0.12-0.32)	0.03 (0.02-0.05)	0.10 (0.06-0.15)	-2.29 (-2.94 to -1.65)	<0.001

Samoa	0.06<br>(0.03-0.12)	0.08<br>(0.03-0.17)	0.03<br>(0.01-0.05)	0.03<br>(0.01-0.07)	-2.98<br>(-3.63 to -2.32)	<0.001
San Marino	0<br>(0-0)	0.03<br>(0.01-0.06)	0<br>(0-0)	0.01<br>(0.01-0.03)	-1.77<br>(-2.1 to -1.43)	<0.001
Sao Tome and Principe	0.07<br>(0.01-0.17)	0.09<br>(0.01-0.24)	0.03<br>(0.01-0.06)	0.03<br>(0.01-0.07)	-4.66<br>(-7.58 to -1.65)	0.003
Saudi Arabia	0.96<br>(0.45-1.84)	0.01<br>(0-0.03)	0.73<br>(0.21-1.42)	0.01<br>(0-0.02)	-1.32<br>(-1.94 to -0.69)	<0.001
Senegal	5.18<br>(0.64-12.8)	0.1<br>(0.01-0.28)	3.27<br>(1.2-6.6)	0.04<br>(0.01-0.1)	-3.51<br>(-5.03 to -1.96)	<0.001
Serbia	2.34<br>(1.16-5.26)	0.09<br>(0.03-0.22)	0.61<br>(0.36-0.92)	0.03<br>(0.01-0.05)	-3.39<br>(-3.8 to -2.98)	<0.001
Seychelles	0.01<br>(0-0.01)	0.02<br>(0.01-0.04)	0<br>(0-0.01)	0.02<br>(0.01-0.04)	-0.67<br>(-1.17 to -0.17)	0.009
Sierra Leone	3.43<br>(0.27-9.98)	0.15<br>(0.01-0.45)	3.74<br>(0.77-9.3)	0.08<br>(0.01-0.24)	-2.51<br>(-3.29 to -1.73)	<0.001
Singapore	0.29<br>(0.14-0.42)	0.03<br>(0.02-0.06)	0.06<br>(0.04-0.08)	0.01<br>(0-0.01)	-5.97<br>(-6.85 to -5.1)	<0.001
Slovakia	1.76<br>(1.17-2.4)	0.11<br>(0.06-0.17)	0.98<br>(0.46-1.5)	0.09<br>(0.04-0.16)	-0.53<br>(-0.98 to -0.08)	0.021
Slovenia	0.15<br>(0.11-0.21)	0.03<br>(0.02-0.05)	0.04<br>(0.03-0.06)	0.01<br>(0.01-0.02)	-3.01<br>(-3.67 to -2.35)	<0.001
Solomon Islands	0.12<br>(0.04-0.27)	0.06<br>(0.01-0.14)	0.14<br>(0.07-0.28)	0.04<br>(0.02-0.09)	-0.99<br>(-1.95 to -0.02)	0.044
Somalia	6.77<br>(0.43-22.89)	0.14<br>(0.01-0.5)	10.62<br>(2.35-27.63)	0.08<br>(0.01-0.23)	-1.74<br>(-2.36 to -1.12)	<0.001
South Africa	22.84<br>(7.62-50.77)	0.13<br>(0.04-0.31)	9.6<br>(6.25-14.53)	0.05<br>(0.03-0.08)	-3.45<br>(-4.05 to -2.85)	<0.001
South Sudan	8.39<br>(0.55-27.87)	0.23<br>(0.01-0.77)	7.25<br>(1.55-18.25)	0.13<br>(0.02-0.35)	-2.20<br>(-2.72 to -1.68)	<0.001
Spain	2.98<br>(2.31-3.74)	0.03<br>(0.02-0.04)	0.87<br>(0.59-1.35)	0.01<br>(0.01-0.02)	-3.56<br>(-4.09 to -3.03)	<0.001
Sri Lanka	2.26<br>(0.89-3.79)	0.03<br>(0.01-0.07)	0.66<br>(0.32-1.21)	0.01<br>(0-0.02)	-3.82<br>(-5.65 to -1.96)	<0.001
Sudan	3.13<br>(0.22-20.01)	0.02<br>(0-0.15)	3.63<br>(1.66-9.33)	0.02<br>(0-0.05)	-1.33<br>(-2.33 to -0.33)	0.009
Suriname	0.33<br>(0.14-0.56)	0.2<br>(0.08-0.38)	0.14<br>(0.08-0.22)	0.07<br>(0.03-0.15)	-3.49<br>(-4.78 to -2.19)	<0.001
Sweden	1.02<br>(0.73-1.31)	0.05<br>(0.03-0.07)	0.42<br>(0.32-0.57)	0.02<br>(0.01-0.03)	-3.68<br>(-4.63 to -2.73)	<0.001
Switzerland	0.69<br>(0.44-0.99)	0.04<br>(0.03-0.07)	0.26<br>(0.18-0.36)	0.02<br>(0.01-0.02)	-3.69<br>(-3.94 to -3.43)	<0.001
Syrian Arab Republic	2.12<br>(0.69-4.43)	0.03<br>(0.01-0.07)	1.15<br>(0.64-1.84)	0.02<br>(0.01-0.05)	-1.26<br>(-1.81 to -0.71)	<0.001

Taiwan (Province of China)	4.24 (2.95-5.47)	0.06 (0.04-0.09)	0.75 (0.51-1.06)	0.02 (0.01-0.03)	-3.97 (-4.81 to -3.12)	<0.001
Tajikistan	11.85 (2.68-25.59)	0.35 (0.08-0.76)	2.83 (1.44-5.36)	0.07 (0.03-0.14)	-5.86 (-6.26 to -5.47)	<0.001
Thailand	5.41 (2.56-9.09)	0.02 (0.01-0.05)	1.27 (0.73-1.95)	0.01 (0-0.02)	-3.47 (-3.82 to -3.11)	<0.001
Timor-Leste	0.21 (0.03-0.61)	0.04 (0.01-0.14)	0.15 (0.06-0.26)	0.02 (0.01-0.05)	-2.86 (-3.58 to -2.14)	<0.001
Togo	2.09 (0.32-5.18)	0.09 (0.01-0.24)	1.79 (0.62-3.63)	0.05 (0.01-0.11)	-2.57 (-2.98 to -2.17)	<0.001
Tokelau	0 (0-0)	0.08 (0.02-0.18)	0 (0-0)	0.03 (0.01-0.07)	-3.15 (-3.95 to -2.35)	<0.001
Tonga	0.05 (0.02-0.11)	0.11 (0.04-0.26)	0.03 (0.02-0.06)	0.07 (0.03-0.15)	-1.67 (-2.26 to -1.09)	<0.001
Trinidad and Tobago	0.12 (0.09-0.17)	0.02 (0.01-0.04)	0.06 (0.04-0.09)	0.02 (0.01-0.04)	-1.60 (-2.83 to -0.35)	0.013
Tunisia	0.48 (0.16-1.73)	0.01 (0-0.05)	0.3 (0.13-0.57)	0.01 (0-0.02)	-1.01 (-1.26 to -0.75)	<0.001
Turkey	44.04 (16.82-89.34)	0.16 (0.04-0.4)	15.11 (9.57-24.06)	0.07 (0.04-0.14)	-2.84 (-3.57 to -2.1)	<0.001
Turkmenistan	3.00 (0.79-6.13)	0.14 (0.04-0.3)	1.10 (0.49-2.08)	0.06 (0.02-0.11)	-3.47 (-3.8 to -3.14)	<0.001
Tuvalu	0.01 (0-0.02)	0.15 (0.02-0.44)	0 (0-0)	0.04 (0.02-0.08)	-4.92 (-5.69 to -4.15)	<0.001
Uganda	14.13 (1.98-42.98)	0.11 (0.01-0.35)	21.56 (8.24-46.89)	0.09 (0.02-0.21)	-1.32 (-2.39 to -0.24)	0.017
Ukraine	5.69 (3.53-8.83)	0.04 (0.02-0.07)	3.51 (2.12-4.98)	0.04 (0.02-0.07)	0.24 (-0.96 to 1.45)	0.696
United Arab Emirates	0.05 (0.01-0.11)	0.01 (0-0.02)	0.06 (0.03-0.11)	0 (0-0.01)	-1.56 (-1.88 to -1.23)	<0.001
United Kingdom	12.62 (9.77-15.37)	0.09 (0.06-0.11)	8.84 (5.98-10.05)	0.06 (0.04-0.07)	-1.34 (-1.67 to -1.01)	<0.001
United Republic of Tanzania	34.34 (5.39-97.23)	0.2 (0.03-0.56)	48 (15.24-107.88)	0.14 (0.04-0.34)	-1.07 (-1.79 to -0.35)	0.004
United States of America	48.04 (42.94-57.48)	0.07 (0.06-0.08)	46.85 (38.93-51.93)	0.06 (0.05-0.07)	-3.76 (-4.48 to -3.03)	<0.001
United States Virgin Islands	0.03 (0.02-0.05)	0.07 (0.03-0.15)	0.01 (0-0.01)	0.02 (0.01-0.06)	-0.43 (-0.84 to -0.02)	0.039
Uruguay	0.26 (0.2-0.34)	0.02 (0.02-0.04)	0.26 (0.17-0.37)	0.03 (0.01-0.04)	0.41 (-0.42 to 1.25)	0.339
Uzbekistan	19.41 (8.35-30.51)	0.16 (0.07-0.27)	12.47 (4.81-19.05)	0.09 (0.04-0.16)	-2.22 (-2.46 to -1.99)	<0.001
Vanuatu	0.06 (0.02-0.12)	0.07 (0.02-0.15)	0.07 (0.03-0.14)	0.05 (0.02-0.11)	-1.05 (-2.55 to 0.47)	0.175

Venezuela (Bolivarian Republic of)	3.14<br>(2.27-4.31)	0.03<br>(0.02-0.05)	1.05<br>(0.69-1.59)	0.01<br>(0.01-0.02)	-3.13<br>(-4.36 to -1.88)	<0.001
Viet Nam	13.66<br>(5.57-27.28)	0.04<br>(0.01-0.09)	4.97<br>(2.67-9.06)	0.02<br>(0.01-0.04)	-2.83<br>(-3.04 to -2.62)	<0.001
Yemen	1.26<br>(0.17-7.13)	0.01<br>(0-0.07)	2.24<br>(1.01-6.08)	0.01<br>(0-0.04)	-0.09<br>(-0.47 to 0.28)	0.622
Zambia	14.79<br>(1.55-44)	0.27<br>(0.03-0.82)	10.87<br>(3.97-23.82)	0.11<br>(0.03-0.26)	-3.65<br>(-4.13 to -3.16)	<0.001
Zimbabwe	5.06<br>(1.32-13.49)	0.08<br>(0.02-0.22)	7.17<br>(2.28-16.39)	0.09<br>(0.02-0.23)	0.26<br>(-1.04 to 1.59)	0.695
